# Efficacy and Safety of *Astragalus*-Containing Traditional Chinese Medicine Combined With Platinum-Based Chemotherapy in Advanced Gastric Cancer: A Systematic Review and Meta-Analysis

**DOI:** 10.3389/fonc.2021.632168

**Published:** 2021-08-04

**Authors:** Mengqi Cheng, Jiaqi Hu, Yuwei Zhao, Juling Jiang, Runzhi Qi, Shuntai Chen, Yaoyuan Li, Honggang Zheng, Rui Liu, Qiujun Guo, Xing Zhang, Yinggang Qin, Baojin Hua

**Affiliations:** ^1^Department of Oncology, Guang’anmen Hospital, China Academy of Chinese Medical Sciences, Beijing, China; ^2^Graduate School, Beijing University of Chinese Medicine, Beijing, China

**Keywords:** *Astragalus*, platinum, advanced gastric cancer, traditional Chinese medicine, meta-analysis

## Abstract

**Background:**

*Astragalus*-containing traditional Chinese medicine (TCM) is widely used as adjunctive treatment to platinum-based chemotherapy (PBC) in patients with advanced gastric cancer (AGC) in China. However, evidence regarding its efficacy remains limited. This study aimed to evaluate the efficacy and safety of *Astragalus*-containing TCM combined with PBC in AGC treatment.

**Methods:**

We searched for literature (up to July 19, 2020) in eight electronic databases. The included studies were reviewed by two researchers. The main outcomes were the objective response rate (ORR), disease control rate (DCR), survival rate, quality of life (QOL), adverse drug reactions (ADRs), and peripheral blood lymphocyte levels. The effect estimate of interest was the risk ratio (RR) or mean difference (MD) with 95% confidence intervals (CIs). Trial sequential analysis (TSA) was used to detect the robustness of the primary outcome and to calculate the required information size (RIS). Certainty of the evidence was assessed using the GRADE profiler.

**Results:**

Results based on available literature showed that, compared with patients treated with PBC alone, those treated with *Astragalus*-containing TCM had a better ORR (RR: 1.24, 95% CI: 1.15–1.34, P < 0.00001), DCR (RR: 1.10, 95% CI: 1.06–1.14, P < 0.00001), 1-year survival rate (RR: 1.41, 95% CI: 1.09–1.82, P = 0.009), 2-year survival rate (RR: 3.13, 95% CI: 1.80–5.46, P < 0.0001), and QOL (RR: 2.03, 95% CI: 1.70–2.43, P < 0.00001 and MD: 12.39, 95% CI: 5.48–19.30, P = 0.0004); higher proportions of CD3^+^ T cells and CD3^+^ CD4^+^ T cells; higher ratio of CD4^+^/CD8^+^ T cells; nature killer cells; and lower incidence of ADRs. Subgroup analysis showed that both oral and injection administration of *Astragalus*-containing TCM increased tumor response. Whether treatment duration was ≥8 weeks or <8 weeks, *Astragalus*-containing TCM could increase tumor response in AGC patients. Furthermore, *Astragalus*-containing TCM combined with oxaliplatin-based chemotherapy could increase the ORR and DCR; when with cisplatin, it could only increase the ORR.

**Conclusion:**

Current low to moderate evidence revealed that *Astragalus*-containing TCM combined with PBC had better efficacy and less side effects in the treatment of AGC; however, more high-quality randomized studies are warranted.

**Systematic Review Registration:**

PROSPERO, identifier CRD42020203486.

## Introduction

As a global health problem, gastric cancer remains the third most common cause of cancer-related death worldwide accounting for 8.2% of cancer-related deaths in 2018, equating to 1 in every 12 deaths (1). Unfortunately, a considerable number of patients with gastric cancer are diagnosed at advanced stage, meaning that the opportunity for surgical treatment has been lost (2). Platinum-based chemotherapy (PBC) is widely used in the treatment of advanced gastric cancer (AGC), and platinum (cisplatin or oxaliplatin) plus fluoropyrimidine regimen is suggested as the first-line chemotherapy regimen according to the National Comprehensive Cancer Network (NCCN) guidelines (version 2.2020) (3, 4). However, in clinical practice, the adverse events of and chemotherapy resistance to PBC have exposed the limitations of PBC, prompting researchers and clinicians to pay more attention to the study of alternative and complementary therapies.

In China, Chinese herb medicine used in Traditional Chinese Medicine (TCM) is frequently combined with chemotherapy in the treatment of AGC; specifically, it has been shown to improve the efficacy of chemotherapy and reduce its side effects (5–7). *Astragalus* (also called *Huangqi* in Chinese) is sourced from the leguminous plant *Astragalus membranaceus (Fisch.)*, which has been recognized as one of the primary tonic herbs in TCM over 2,000 years and is widely used in the treatment of malignant tumors in China.

Many researchers have examined the effectiveness and safety of *Astragalus*-containing TCM combined with PBC in the treatment of colorectal cancer and non-small-cell lung cancer (8–11). However, the effects of *Astragalus*-containing Chinese medicines plus PBC in AGC treatment have not been systematically assessed.

In recent decades, a large number of trials on the use of *Astragalus*-containing Chinese herbal therapies combined with PBC for AGC treatment have been published. This study aimed to provide a systematic analysis of the results obtained in these studies in order to understand the safety and efficacy of *Astragalus*-containing TCM in combination with PBC during AGC treatment. To do so, we performed a meta-analysis of the available studies on *Astragalus*-containing TCM combined with PBC in order to further clinically investigate the effects on safety and efficacy.

## Methods

This study was conducted following the Preferred Reporting Items for Systematic Reviews and Meta-Analyses (PRISMA) guidelines. The protocol has been registered on PROSPERO, under the number CRD42020203486.

### Search Strategy

PubMed, EMBASE, Cochrane Central Register of Controlled Trials (CENTRAL), clinicaltrials.gov, Chinese Biomedical Literature database (CBM), China Academic Journals (CNKI), Chinese Science and Technology Journals (CQVIP), and Wanfang Database were searched systematically for all articles published from inception to July 19, 2020. The following search terms were used: ([gastr* OR stomach* OR digest* OR epigastr*] AND [carcin* OR cancer* OR neoplas* OR tumour* OR tumor* OR growth* OR adenocarcin* OR malig*]) AND (*Astragalus* OR radix astragali OR huang qi OR huangqi). All retrievals were implemented using MeSH and free words (the detailed search strategy is available in **Supplementary 1**). The languages were restricted to Chinese and English.

### Inclusion and Exclusion Criteria

Inclusion criteria: (1) All studies were randomized controlled trials (RCTs) or quasi-RCTs. (2) Patients who had TNM stage III-IV AGC and were diagnosed using the histopathological and cytological diagnostic criteria. (3) The experimental group of patients was given *Astragalus*-based herbal therapy combined with PBC. Any form of *Astragalus* (Huang qi) preparation, including water decoction, extracts, granules, or injection, among other forms, regardless of administration route, were included. The control group of patients was given PBC alone. (4) Outcomes were identified as the tumor response, survival rate, QOL, ADRs, and peripheral blood lymphocytes levels, and at least one of these outcomes was reported; and (5) for repeated publication studies, we selected the data with the most comprehensive report and the longest follow-up.

Exclusion Criteria: (1) Patients who underwent radiotherapy, chemotherapy, or other antitumor therapy within 1 month before treatment; (2) patients with severe infection, other malignant tumors, and severe medical diseases; (3) the prescription of *Astragalus*-based herbal therapy was not fixed; (4) studies for which the data could not be extracted; and (5) the baseline data of patients in two groups were not comparable.

### Study Selection and Data Extraction

Studies were selected independently by two reviewers according to the above inclusion and exclusion criteria. Two reviewers independently extracted the data. Disagreements were discussed with and resolved by the third reviewer. The following data were extracted: first author (year of publication), sample sizes, gender, age, study arm, drug delivery, treatment duration, follow-up time, outcomes, and criteria. Data presented graphically were extracted using WebPlotDigitizer (https://automeris.io/WebPlotDigitizer).

### Risk of Bias Assessment

Two reviewers independently assessed the quality of the selected studies according to the Cochrane Collaboration’s tool for RCTs. Items were sorted into three categories: low risk of bias, unclear bias, and high risk of bias. The following characteristics were evaluated: Random sequence generation (selection bias), allocation concealment (selection bias), blinding of participants and personnel (performance bias), incomplete outcome data (attrition bias), selective reporting (reporting bias), and other biases. Results from these evaluations were graphed and assessed using Review Manager 5.3.

### Outcome Definition

Tumor response and the survival rate were the main outcomes. Tumor response, containing the objective response rate (ORR) and disease control rate (DCR), was based on the World Health Organization (WHO) criteria (12) or Response Evaluation Criteria in Solid Tumors (RECIST) (13). Complete response (CR), partial response (PR), stable disease (SD), and progressive disease (PD) were used as indicators. The CR plus PR rates were as equal to the ORR; the CR plus PR, and SD rates were equal to the DCR.

Quality of life (QOL), adverse drug reactions (ADRs), and peripheral blood lymphocytes levels were the second outcomes. QOL was considered improved when the Karnofsky Performance Status (KPS) score was 10 points higher after treatment than before treatment. ADRs were accessed by measuring hematotoxicity (neutropenia, anemia, and thrombocytopenia), gastrointestinal toxicity (nausea and vomiting, diarrhea), hepatic and renal dysfunction, neurotoxicity, alopecia, and stomatitis, based on WHO criteria (12) or NCI Common Terminology Criteria for Adverse Events (CTCAE) (14). The peripheral blood lymphocyte levels were evaluated by measuring the T-lymphocyte subsets such as the percentage of CD3^+^, CD3^+^CD4^+^, and CD3^+^CD8^+^ T cells, as well as the CD4^+^/CD8^+^ T cells ratio and the percentage of natural killer (NK) cells.

### Data Analysis

The Review Manager 5.3 and Stata V16.0 software were used in this study. The risk ratio (RR) and 95% confidence interval (CI) were used to assess dichotomous variables, while mean difference (MD) with a 95% CI was used to assess continuous variables. Statistical heterogeneity was measured by the I^2^ statistic and the Chi-squared test. The summary RR, MD, and 95% CI were estimated by a random-effect model. P values were used to calculate outcomes, and P < 0.05 was considered statistically significant. The funnel plots and Egger’s tests were applied to examine potential publication bias when studies ≥10. In accordance with the drug delivery of *Astragalus*-containing TCM, PBC regimen, and treatment duration, subgroup analyses were performed to reveal the clinical heterogeneity and its influence on tumor response.

Trial sequential analysis software (TSA, version 0.9.5.10 beta) was used to determine the robustness of main outcomes and to calculate the required information size (RIS) in the meta-analysis (15). If the cumulative Z-curve stretched across the monitoring boundaries, a sufficient level of evidence for the intervention effect may have been reached and no further studies were needed. Type I and II errors were needed to perform the TSA. In this review, the RIS was estimated using α = 0.05 (two sided) with power equal to 80%.

### Evidence Quality Assessment

Two reviewers independently assessed the quality of the evidence for each outcome using the GRADE approach (16). Disagreements were discussed with and resolved by the third reviewer. The quality of evidence was classified as being high, moderate, low, and very low. The quality was downgraded according to the following five domains: (1) risk of bias (if most trials showed an unclear risk, with or without high risk, but the results had good robustness, the evidence was rated down by only one level; if the results showed poor robustness, the evidence was downgraded by two levels); (2) inconsistency (statistical heterogeneity was present and the results of the sensitivity analysis had poor robustness); (3) indirectness (the participants, intervention, outcomes, or comparison of the study did not meet the objectives of this study); (4) imprecision (the sample size for each outcome was fewer than 300 cases); and (5) reporting bias (publication bias). Except for the risk of bias, evidence was downgraded by one level.

## Results

### Search Results

**Figure 1:**
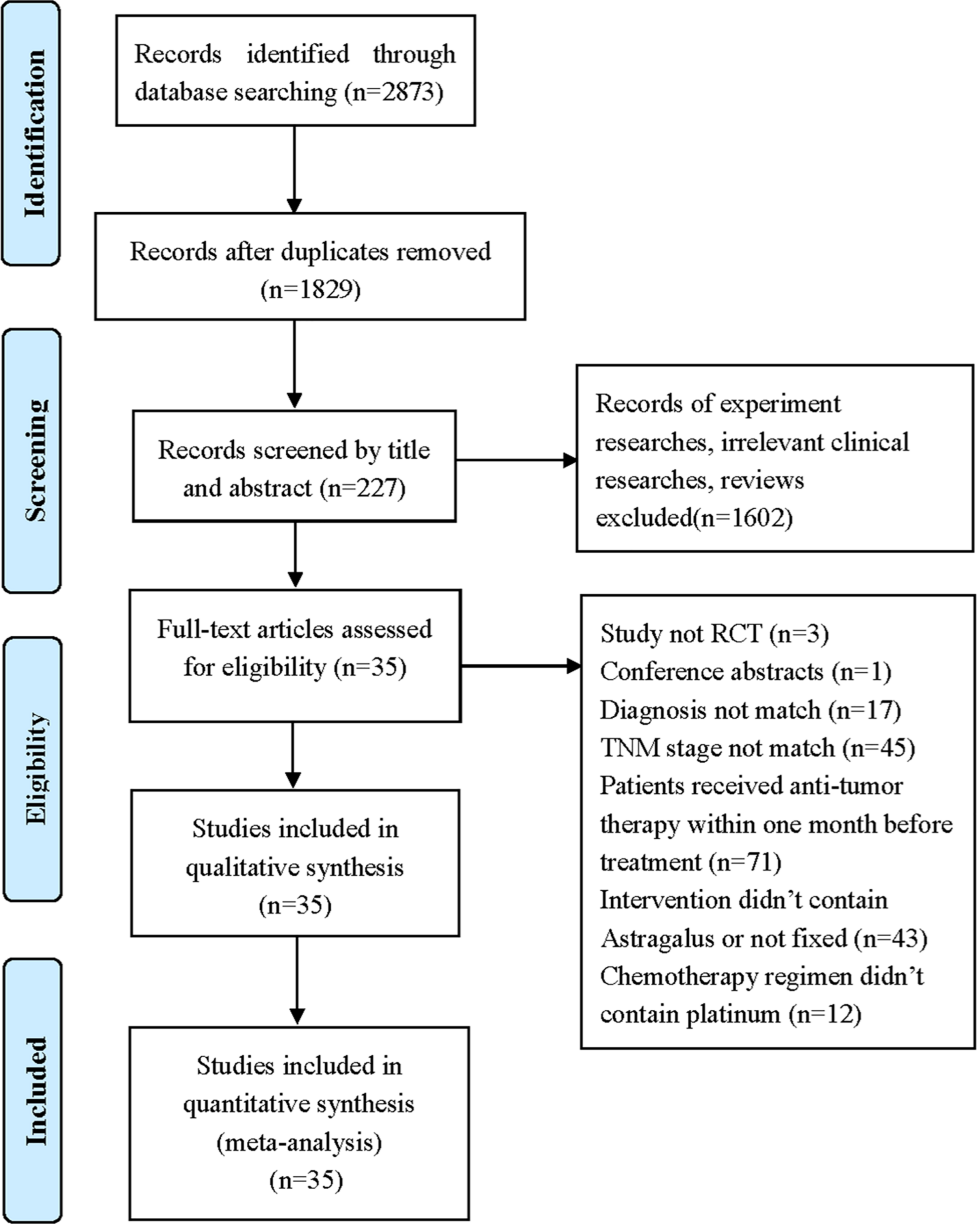
Flow diagram of selection process (17).

A total of 2,873 records were identified through database searching. All records were screened by two reviewers with a three-step process. Firstly, we screened the titles and removed duplicates, after which 1,829 records remained. Secondly, we included 227 full texts by reading the title or abstract. Thirdly, we evaluated the full texts and excluded 192 trials by following the inclusion and exclusion criteria. In the end, 35 eligible studies involving 2,670 stage III/IV AGC patients were included in our study (**Figure 1**).

### Characteristics of the Included Studies

The characteristic information of the included studies is shown in **Table 1**. All 35 studies were single-centered and conducted in China. There were 1,572 male and 1,098 female patients, and sample sizes ranged from 30 to 240. Eighteen studies used the oral TCM administration, and 17 studies used commercial TCM injections. All clinical trials were designed to use *Astragalus*-containing TCM as the principal drug together with PBC. Follow-up lasted from 4 weeks to 3 years. Twenty trials reported the tumor responses according to the WHO guidelines, and 12 reported them according to RECIST guidelines. Eighteen trials reported the ADRs using WHO criteria, three using NCI CTCAE criteria, and eight using unknown criteria.

**Table 1:** Characteristics of the included studies.

Study	Advanced gastric cancer (AGC)			Interventions			Fellow up	Criteria	Outcome
	E/C	M/F	TNM stage	*Astragalus*-containing TCM	Drug delivery	Platinum-based regimen			
Yang, J. 2005 (18)	16/16	22/10	IV	*Astragalus*-based formulae: Astragalus, Codonopsis, Atractylodis macrocephala, Poria cocos, Pinellia ternate, Citrus reticulata, Fructus aurantii, Coix seed, Dioscoreae opposita, Hedyotis diffusa, Nightshade, Curcuma zedoary, Rice sprout, Malt, Radix scutellariae, Glycyrrhiza, 300 ml/d, 8w	Orally	5-Fu: 250 mg/m^2^, d1–14; OXA: 130 mg/m^2^, d1, d8, d15, 28d/C; 2 cycles	8w	WHO, WHO	O1,2,4,5
Gong, L.Y. 2006 (19)	26/30	31/25	IIIB: 17, IV: 9/IIIB: 19, IV: 11	Aidi: Astragalus, Ginseng, Cantharides, Eleutherococcus senticosus, 50 ml/d, 21d/C, 4 cycles	Injection	PTX: 135 mg/m^2^, d1; 5-Fu: 500 mg/m^2^, d1–5; CF: 100 mg/m^2^, d1–5; DDP: 30 mg/m^2^, d1–3, 3w/C, 4 cycles	>12m	RECIST, WHO	O1,2,3,5,6
Chen, P. 2007 (20)	64/64	90/38	IV	Cidan Capsule: Astragalus, Curcuma zedoary, Brucea javanica, Pleione bulbocodioides, Semen strychni, Nidus vespae, 5.4 g/d, 28d/C, 3 cycles	Orally	OXA: 100 mg, d1, d8; CF: 50 mg, d1–5; 5-Fu: 0.5–0.75 g, d1–5; 4w/C, 3 cycles	12w	WHO, WHO	O1,2,5
Li, A.M. 2007 (21)	64/64	86/42	III, IV	*Astragalus*-based formulae: Astragalus, Codonopsis, Atractylodis macrocephala, Poria cocos, Radix ophiopogonis, Spatholobus suberctu, Radix salviae miltiorrhizae, Ligustrum lucidum ait, Hedyotis diffusa, 400 ml/d, d1–21, 28d/C, 3 cycles	Orally	DDP: 20 mg/m^2^, d1–5; LV: 200 mg/m^2^, d1–5; 5-FU: 500 mg/m^2^, d1–5; 4w/C, 3 cycles	12w	WHO, WHO	O1,2,5
Wang, L. 2007 (22)	15/15	23/7	IIIB, IV	*Astragalus*-based formulae: Astragalus, Atractylodis macrocephala, Radix pseudostellariae, Noria cocos, Dioscoreae opposita, Pinellia ternate, Citrus reticulata, Coix seed, Galli gigerii endothelium corneum, Hedyotis diffusa, Scutellariae barbatae, Rice sprout, Malt, Glycyrrhiza, 300 ml/d, 8w	Orally	PTX: 135 mg/m^2^, d1, d8; OXA: 130 mg/m^2^, d1, d8; 5-Fu: 500 mg/m^2^, d1–5; 4w/C, 2 cycles	>8w	WHO, WHO	O1,2,4,5
He, Z.Q. 2008 (23)	65/58	68/55	IV	Delisheng: Astragalus, Red ginseng, Arenobufagin, Cantharides, 40 ml/d, 20 d/C, 4–6 cycles	Injection	OXA: 85 mg/m^2^, d1; CF: 200 mg/m^2^, d1–2; 5-Fu: 400 mg/m^2^, d1–2; 5-Fu: 600 mg/m^2^, d1–2; 2w/C, 4–6 cycles	Unknow	RECIST, WHO	O1,2,5,6
Liu, L.H. 2009 (24)	30/30	34/26	IIIB: 17, IV: 13/IIIB: 14, IV: 16	Aidi: Astragalus, Ginseng, Cantharides, Eleutherococcus senticosus, 50 ml/d, d1–10, 28d/C, 2 cycles	Injection	PTX: 175 mg/m^2^, d1; DDP: 20 mg/m^2^, d1–5; 5-Fu: 600 mg/m^2^, d1–5; 28d/C, 2 cycles	8w	WHO, WHO	O1,2,4,5
Liu, X.Q. 2009 (25)	30/30	42/18	IV	Kangai: Astragalus, Ginseng, Matrine, 60 ml/d, 10d/C, 2 cycles	Injection	CF: 200 mg/m^2^, d1–2; 5-Fu: 2.0 g/m^2^,48 h; DOC: 50 mg/m^2^, d1; DDP: 25 mg/m^2^, d2–3; 14d/C; 2 cycles	4w	RECIST, NCI	O1,2,4,5
Zhu, Y. 2010 (26)	20/20	23/17	III, IV	*Astragalus*-based formulae: Astragalus, Codonopsis, Angelica sinensis, Paeonia lactiflora, Costusroot, Bupleurum, Coix seed, Notoginseng radix, Fructus aurantii, Rhizoma bletillae, Curcuma zedoary, Glycyrrhiza, Radix platycodi, Fritillary bulb, Crotonis fructus, 400 ml/d, 6w	Orally	OXA: 130 mg/m^2^, d1; CF: 300 mg, d1–5; 5-Fu: 500 mg, d1–5; 3w/C, 2 cycles	6w–1y	RECIST, WHO	O1,2,3,4,5
Chen, Q.S. 2011 (27)	30/30	37/23	III: 17, IV: 13/III: 19, IV: 11	*Astragalus*-based formulae: Astragalus, Codonopsis, Atractylodis macrocephala, Noria cocos, Ninellia ternate, Ganoderma, Agrimonia pilosa, Prunellae spica, Hedyotis diffusa, Glycyrrhiza, 400 ml/d, 21d/C, 2 cycles	Orally	OXA: 85 mg/m^2^, d1; CF: 200 mg/m^2^, d1–2; 5-Fu: 400 mg/m^2^, d1–2; 5-Fu: 600 mg/m^2^, d1–2; 21d/C, 2 cycles	6w–1y	WHO,Un	O1,2,3,4,5,6
Du, C.J. 2011 (28)	120/120	122/118	IV	*Astragalus*-based formulae: Astragalus, Atractylodis macrocephala, Poria cocos, Pinellia ternate, Citrus reticulata, Rhizoma polygonati, Glycyrrhiza, Ligustrum lucidum ait, Sanguisorbae radix, Spatholobus suberctu, Donkey-hide glue, Radix actinidiae, 400 ml/d, 21d/C, 2 cycles	Orally	OXA: 100 mg/m^2^, d1; CF: 200 mg/m^2^, d1–5; 5-Fu: 500 mg/m^2^, d1–5; 21d/C, 2 cycles	6w	RECIST	O1,2
Fan, C.M. 2011 (29)	23/28	33/18	IIIA: 8, IIIB: 10, IV: 5/IIIA: 10, IIIB: 13, IV:5	Aidi: Astragalus, Ginseng, Cantharides, Eleutherococcus senticosus, 50 ml/d,10d/C, 4 cycles	Injection	OXA: 85 mg/m^2^, d1; S-1: 80 mg/m^2^, d1–14; 21d/C, 4 cycles	>12w	WHO, WHO	O1,2,3,4,5
Hu, F.S. 2011 (30)	51/48	69/30	IV	*Astragalus*-based formulae: Astragalus, Codonopsis, Radix pseudostellariae, Atractylodis macrocephala, Poria cocos, Fructus lycii, Ligustrum lucidum ait, Semen cuscutae, Spatholobus suberctu, Paeonia rubra, Curcuma zedoary, Polyphylla, Hedyotis diffusa, Radix actinidiae, 400 ml/d, 21d/C, 3 cycles	Orally	CAP: 1,000 mg/m^2^, d1–14; OXA: 130 mg/m^2^, d1; 21d/C, 3 cycles	9w	WHO, WHO	O1,2,4,5,6
Ren, Y.Z. 2012 (31)	33/32	30/35	IV	Shenqi Fuzheng: Astragalus, Codonopsis, 250 ml/d, 14d/C, 3 cycles	Injection	OXA: 85 mg/m^2^, d1; CF: 300 mg/m^2^, d1–2; 5-Fu: 400 mg/m^2^, d1–2; 5-Fu: 600 mg/m^2^, d1–2; 2w/C, 3 cycles	6w	WHO	O1,2
Wang, J.R. 2012 (32)	26/24	32/18	III: 15, IV: 11/III: 14, IV: 10	Shenqi Fuzheng: Astragalus, Codonopsis, 250 ml/d, 14d/C, 2 cycles	Injection	DOC: 75 mg/m^2^, d1; DDP: 20 mg/m^2^, d1–5; 5-Fu:750 mg/m^2^, d1–5; 28d/C, 2 cycles	8w-24w	RECIST, WHO	O1,2,4,5
Li, H.Y. 2013 (33)	35/35	35/35	III: 14, IV: 21/III: 16, IV: 19	Shenqi Fuzheng: Astragalus, Codonopsis, 250 ml/d,14d/C, 4 cycles	Injection	OXA: 85 mg/m^2^, d1; CF: 200 mg/m^2^, d1–2; 5-Fu: 400 mg/m^2^, d1–2; 5-Fu: 600 mg/m^2^, d1–2; 2w/C, 4 cycles	8w	WHO,Un	O1,2,5
Tan, G. 2013 (34)	26/22	26/22	IV	*Astragalus*-based formulae: Astragalus, Codonopsis, Atractylodis macrocephala, Poria cocos, Dioscoreae opposita, Citrus reticulata, Amomum, Galli gigerii endothelium corneum, Aflatoxin, Salviae miltiorrhizae, Cowherb seed, Hedyotis diffusa, Ganoderma, Scutellariae barbatae, 400 ml/d, 28d/C, 2 cycles	Orally	OXA: 85 mg/m^2^, d1; CF: 200 mg/m^2^, d–5; 5-Fu: 400 mg/m^2^, d1–2; 5-Fu: 600 mg/m^2^, d1–2; 2w/C, 4 cycles	8w	WHO, WHO	O1,2,4
Yin, L.L. 2013 (35)	26/27	27/26	III: 16, IV: 10/III: 14, IV: 13	Shenqi Fuzheng: Astragalus, Codonopsis, 250 ml/d, 14d/C, 2 cycles	Injection	S-1: 120 mg/d, d1–21; DDP: 20 mg/m^2^, d1–5; 4w/C, 2 cycles	8w	WHO, NCI	O1,2,5,6
Fei, Y.H. 2014 (36)	40/40	54/26	III: 21, IV: 19/III: 18, IV: 22	*Astragalus*-based formulae: Astragalus, Codonopsis, Ganoderma, Atractylodis macrocephala, Poria cocos, Radix ophiopogonis, Polyporus umbellatus, Pinellia ternate, Fructus aurantii, Coix seed, Hedyotis diffusa, Curcuma, Bambusae caulis in taenias, 400 ml/d, 3w	Orally	DOC: 40 mg/m^2^, d1, d8; DDP: 15 mg/m^2^, d1–5; 5-Fu, 600 mg/m^2^, d1–5; 28d/C, 2 cycles	6w	WHO	O1,2,6
Wen, J. 2014 (37)	15/15	23/7	IIIB: 5, IIIC: 6, IV: 4/IIIB: 7, IIIC: 4, IV: 4	Shenqi Fuzheng: Astragalus, Codonopsis, 250 ml/d, 14d/C, 4 cycles	Injection	OXA: 85 mg/m^2^, d1; CF: 200 mg/m^2^, d1–2; 5-Fu: 400 mg/m^2^, d1–2; 5-Fu: 600 mg/m^2^, d1–2; 2w/C, 4 cycles	8w	RECIST, WHO	O1,2,4,5
Xiang, S.L. 2014 (38)	33/33	28/38	IIIB: 19, IV: 14/IIIB: 18, IV: 15	Aidi: Astragalus, Ginseng, Cantharides, Eleutherococcus senticosus, 50 ml/d, d1–10, 28d/C, 2 cycles	Injection	PTX: 175 mg/m^2^, d1; DDP: 20 mg/m^2^, d1–5; 5-Fu: 600 mg/m^2^, d5–9; 28d/C, 2 cycles	8w	WHO, WHO	O1,2,4,5
Zhang, L. 2014 (39)	32/32	35/29	IIIA: 6, IIIB: 27, IV: 31	Aidi: Astragalus, Ginseng, Cantharides, Eleutherococcus senticosus, 50–80 ml/d, 21d/C, 2 cycles	Injection	OXA: 85 mg/m^2^, d1; CF: 200 mg/m^2^, d1–2; 5-Fu: 400 mg/m^2^, d1–2; 5-Fu: 600 mg/m^2^, d1–2; 2w/C, 2 cycles	6w	WHO, WHO	O1,2,5
Zhang, M.J. 2014 (40)	48/48	54/42	IIIB: 27, IV: 21/IIIB: 30, IV: 18	Aidi: Astragalus, Ginseng, Cantharides, Eleutherococcus senticosus, 50 ml/d, 21d/C, 2 cycles	Injection	OXA: 130 mg/m^2^, d1; 5-Fu: 400 mg/m^2^, d1; 5-Fu: 2,600 mg/m^2^, d1–5; CF: 200 mg/m^2^, d1; 21d/C; 2 cycles	6w	RECIST, Un	O1,4,5
Zhang, Y.N. 2015 (41)	46/38	49/35	IV	*Astragalus*-based formulae: Astragalus, Codonopsis, Radix pseudostellariae, Atractylodis macrocephala, Poria cocos, Fructus lycii, Ligustrum lucidum ait, Semen cuscutae, Spatholobus suberctu, Paeonia rubra, Curcuma zedoary, Polyphylla, Hedyotis diffusa, Radix actinidiae, 400 ml/d, 21d/C, 3 cycles	Orally	CAP: 1,000 mg/m^2^, d1–14; OXA: 130 mg/m^2^, d1; 21d/C, 3 cycles	9w	WHO	O1,2,4,6
Duan, F. 2016 (42)	46/46	51/41	IIIB: 35, IV: 11/IIIB: 33, IV: 13	Aidi: Astragalus, Ginseng, Cantharides, Eleutherococcus senticosus, 50 ml/d, 21d/C, 2 cycles	Injection	PTX: 175 mg/m^2^, d1; DDP: 25 mg/m^2^, d1–5; 5-Fu: 600 mg/m^2^, d5–9; 28d/C, 2 cycles	8w	WHO, WHO	O1,2,4,5
Hu, Q. 2016 (43)	21/21	18/24	III, IV	Weining Granule: Astragalus, Hedyotis diffusa, Curcuma zedoary, Fructus lycii, Poria cocos, 400 ml/d, 28d/C, 3 cycles	Orally	OXA: 130 mg/m^2^, d1; S-1: BSA <1.25 m^2^, 80mg/d, 1.25–1.50 m^2^, 120 mg/d, >1.25 m^2^,160 mg/d; 42d/C, 2 cycles	12w	WHO	O4,5,6
Huang, P. 2016 (44)	34/33	36/31	IIIB-IV	*Astragalus*-based formulae: Astragalus, Radix pseudostellariae, Coix seed, Paeonia lactiflora, Agrimonia pilosa, Pinellia ternate, Citrus reticulata, Radix ophiopogonis, Curcuma zedoary, Hedyotis diffusa, Rice sprout, Malt, 200 ml/d, 21d/C, 2 cycles	Orally	OXA: 130 mg/m^2^, d1; S-1: 80 mg/m^2^, d1–14; 21d/C, 2 cycles	6w	WHO, Un	O1,2,3,4,5,6
Ma, M. 2016 (45)	22/22	26/18	III, IV	*Astragalus*-based formulae: Astragalus, Ground beeltle, Radix cynanchi panicullati, 400 ml/d, 21d/C, 2 cycles	Orally	OXA: 130 mg/m^2^, d1; S-1: 80 mg/m^2^, d1–14; 21d/C, 2 cycles	6w	WHO, Un	O1,2,4,5
Shao, K.F. 2016 (46)	31/31	34/28	III: 17, IV: 14/III: 15, IV: 16	*Astragalus*-based formulae: Astragalus, Codonopsis, Atractylodis macrocephala, Poria cocos, Coix seed, Semen lablab album, Amomum, Angelica Sinensis, Rhizoma cimicifugae, Dioscoreae opposita, Lotus seed, Bupleurum, Citrus reticulata, Radix platycodi, Glycyrrhiza, 400 ml/d, 4w	Orally	OXA: 85 mg/m^2^, d1; CF: 200 mg/m^2^, d1–2; 5-Fu: 400 mg/m^2^, d1–2; 5-Fu: 600 mg/m^2^, d1–2; 2w/C, 2 cycles	4w	WHO	O5
Hu, Q. 2017 (47)	18/18	22/14	IIIA: 5, IIIB: 6, IV: 7/IIIA: 6, IIIB: 8, IV: 4	Kangai: Astragalus, Ginseng, Matrine, 50 ml/d, d1–14, 21d/C, 6 cycles	Injection	OXA: 130 mg/m^2^, d1; S-1: 120 mg/m^2^, d1–14; 21d/C, 6 cycles	>2y	RECIST, NCI	O1,2,3,5
Pan, B.Y. 2018 (48)	58/54	60/52	IV	Bo-Er-Ning Capsule: Astragalus, Ligustrum lucidum ait, Pleione bulbocodioides, Purslane, Rhizoma paridis, Nightshade, Perilla fruit, Galli gigerii endothelium corneum, Rhubarb, Ornel, Bombyx batryticatus, 1.8g/d, d1–14; 21d/C, 3 cycles	Orally	Tegafur: 80–120 mg/d, d1–14; DDP: 40 mg, d1–3; 21d/C, 3 cycles	3y	/	O3,4
Xu, W. 2019 (49)	66/66	79/53	III: 29, IV: 37/III: 30, IV: 36	*Astragalus*-based formulae: Astragalus, Codonopsis, Atractylodis macrocephala, Poria cocos, Pinellia ternate, Citrus reticulata, Coix seed, Dioscoreae opposita, Atractylodis macrocephala, Hedyotis diffusa, Glycyrrhiza, 400 ml/d, 8w	Orally	OXA: 85 mg/m^2^, d1; CF: 200 mg/m^2^, d1–2; 5-Fu: 400 mg/m^2^, d1–2; 5-Fu: 600 mg/m^2^, d1–2; 2w/C, 4 cycles	8w	RECIST,Un	O1,2,5,6
Yuan, D.D. 2019 (50)	60/60	76/44	III: 22, IV: 38/III: 24, IV: 36	Shenqi Fuzheng: Astragalus, Codonopsis, 250 ml/d,14d/C, 4 cycles	Injection	DOC: 75 mg/m^2^, d1; DDP: 20 mg/m^2^, d1-5; 5-Fu: 750 mg/m^2^, d1–5; 28d/C, 4 cycles	16w	WHO,Un	O1,2,5
Zhu, Y.F. 2019 (51)	30/30	48/12	IV	Aidi: Astragalus, Ginseng, Cantharides, Eleutherococcus senticosus, 40 ml/d, 21d/C, 4 cycles	Injection	S-1: BSA<1.25 m^2^, 80 mg/d, >1.25 m^2^, 120 mg/d, d1–14; OXA: 130 mg/m^2^, d1; DOC: 75 mg/m^2^, d1; 21d/C, 4 cycles	12w	RECIST, WHO	O1,2,4,5
Hou, Y. 2020 (52)	45/45	49/41	IV	*Astragalus*-based formulae: Astragalus, Atractylodis macrocephala, Glycyrrhiza, Ginseng, Angelica sinensis, Rhizoma cimicifugae, Bupleurum, Citrus reticulata, 400 ml/d, 6w	Orally	CAP: 1,000 mg/m^2^, d1–14; OXA: 130 mg/m^2^, d1; 21d/C, 2 cycles	6w–2y	RECIST,Un	O1,2,3,5

E/C, experimental group (Astragalus-containing TCM with PBC)/control group (PBC alone); PBC, platinum-based chemotherapy; M/F, male/female; 5-Fu, 5-fluorouracil; OXA, oxaliplatin; PTX, paclitaxel; DDP, cisplatin; CF, Calcium folinate; DOC, docetaxel; BSA, body surface area; CAP, capecitabine; WHO, World Health Organization guidelines for solid tumor responses; RECIST, Response Evaluation Criteria in Solid Tumors; Un, unclear; O, outcomes; O1, objective response rate (ORR); O2, disease control rate (DCR); O3, survival rate; O4, quality of life (QOL); O5, adverse drug reactions (ADRs); O6, peripheral blood lymphocyte levels.

### Risk of Bias

**Figure 2:**
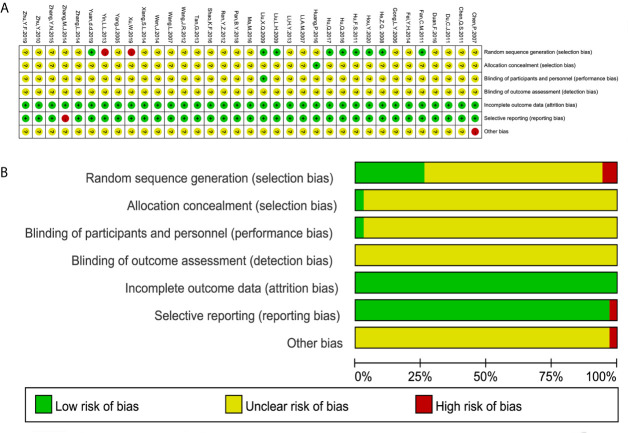
Risk of bias summary and diagram. **(A)** Risk of bias summary. **(B)** Risk of bias diagram.

All 35 studies referred to randomization; however, only eight studies (23, 25, 29, 30, 43, 47, 50, 52) used a random number table to generate a random sequence, one study used a lottery approach (24), and two studies (35, 49) used admission order, which was inappropriate. One study used an envelope method (44) to perform the allocation concealment, while the other studies did not report the method. Only one study mentioned single blinding (25), and none of the studies adequately reported the blinding of the investigators, patients, and outcome assessors. Selective reporting existed in one study (40) that failed to completely report the DCR. One study (20) had a high risk of other bias because it was funded by the industry. The risk of bias for the individual trials is summarized in **Figure 2**.

### Tumor Response

**Figure 3:**
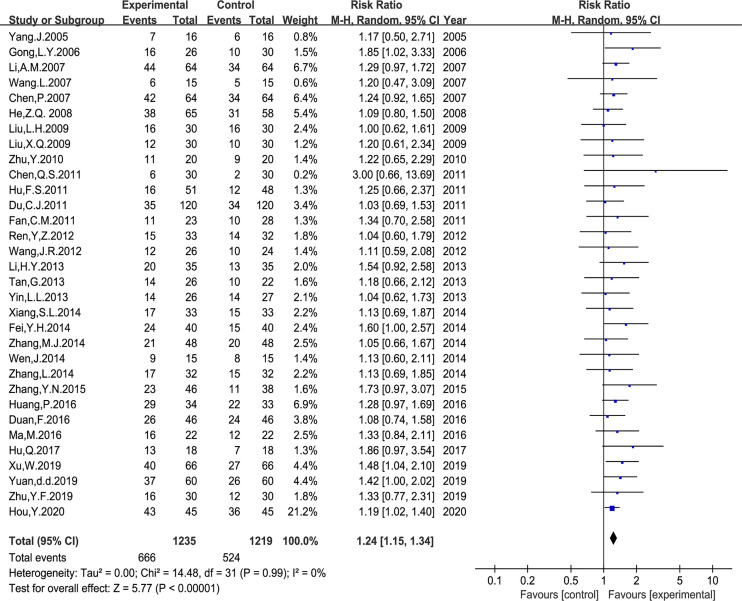
Meta-analysis results of objective response rate (ORR) between the two groups.

Thirty-two trials with 2,454 patients reported the ORR following WHO or RECIST guidelines. The random-effects meta-analysis showed that *Astragalus*-based herbal therapy plus PBC enhanced the ORR, which showed a statistically significant difference (RR: 1.24, 95% CI: 1.15–1.34, P < 0.00001, I^2^ = 0%, **Figure 3**).

**Figure 4:**
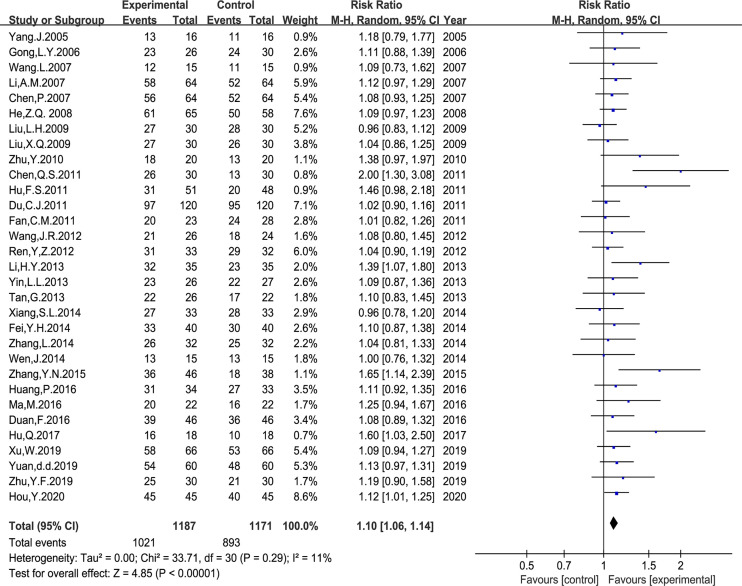
Meta-analysis results of disease control rate (DCR) between the two groups.

Thirty-one trials with 2,358 cases reported the DCR. The meta-analysis using a random-effects model showed that *Astragalus*-based TCM plus PBC increased the DCR; the difference was statistically significant (RR: 1.10, 95% CI: 1.06–1.14, P < 0.00001, I^2^ = 11%, **Figure 4**).

### Survival Rate

**Figure 5:**
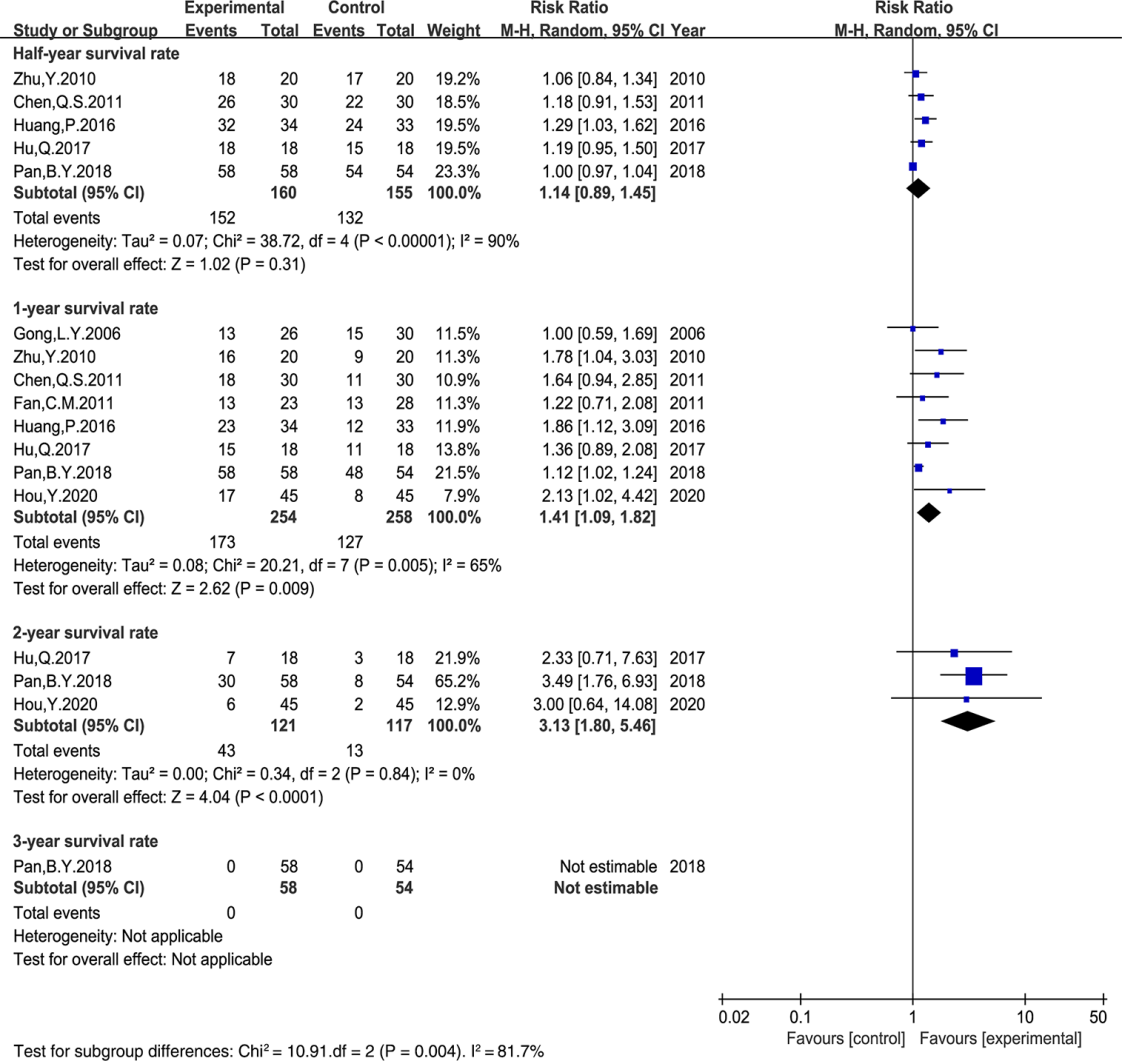
Meta-analysis results of survival rate between the two groups.

Six trials (19, 26, 27, 29, 44, 52) reported the survival rate (**Figure 5**). Additionally, in two trials (47, 48), the survival rate was extracted from survival curves. Thus, eight trials containing 512 cases were included. The results demonstrated that there was no significant difference in the half-year survival rate between the two groups (RR: 1.14, 95% CI: 0.89–1.45, P = 0.31, I^2^ = 90%). However, compared with the PBC-treated control group, the 1- and 2-year survival rates in the *Astragalus*-based TCM group were significantly improved (RR: 1.41, 95% CI: 1.09–1.82, P = 0.005, I^2 ^= 65%; RR: 3.13, 95% CI: 1.80–5.46, P = 0.84, I^2^ = 0%).

### Quality of Life (QOL)

**Figure 6:**
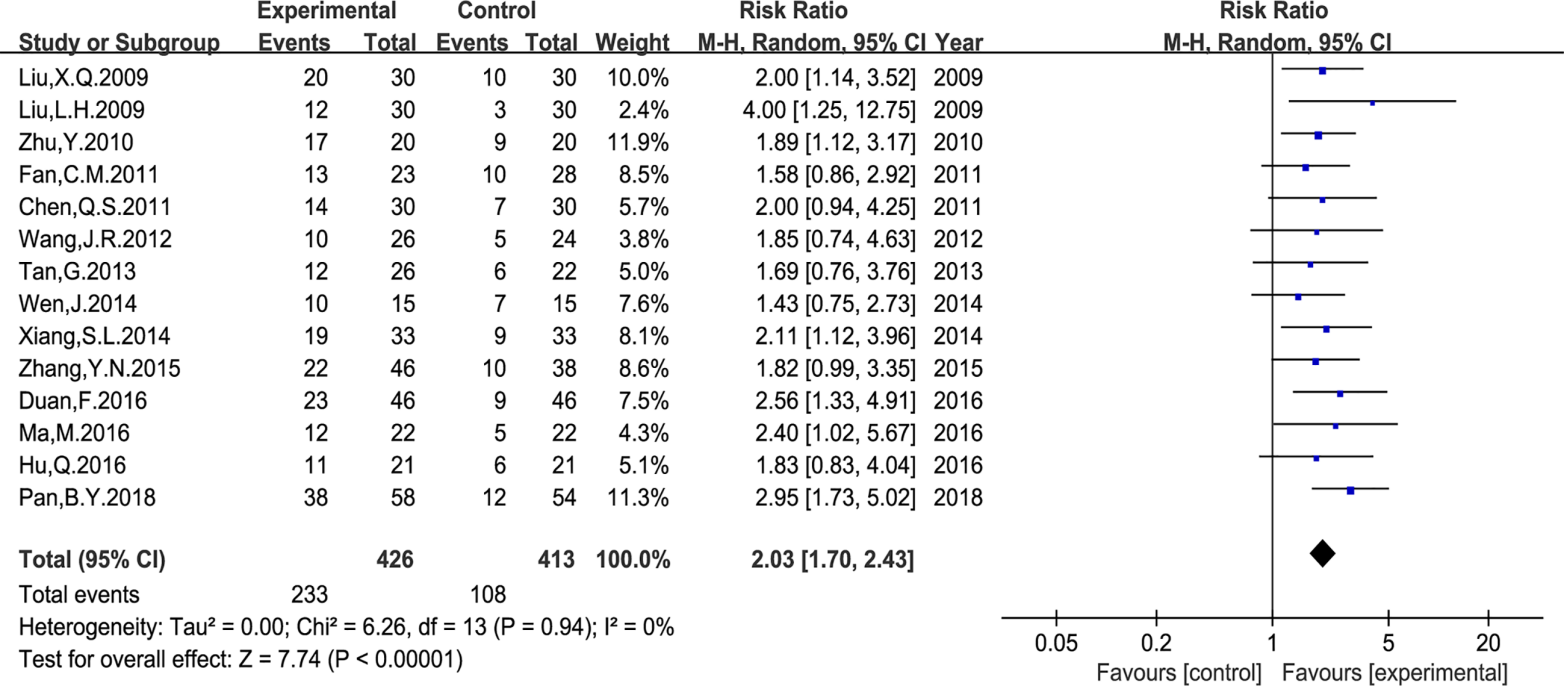
Meta-analysis results of quality of life (QOL) according to the number of KPS improved patients.

**Figure 7:**
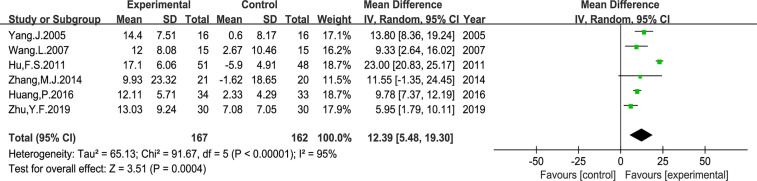
Meta-analysis results of QOL according to mean ± SD.

In the included studies, two types of data were applied to report the QOL changes on KPS score. First, the number of patients who reported QOL improvement (KPS score 10 points higher after treatment). And second, the mean ± standard deviation (SD) of the KPS score before and after treatment. As shown in **Figure 6**, the number of patients with improved QOL based on the KPS score were reported in 14 studies (RR: 2.03, 95% CI: 1.70–2.43, P < 0.00001, I^2^ = 0%); and the mean ± SD of KPS score were reported in other six studies (MD: 12.39, 95% CI: 5.48–19.30, P *=* 0.0004, I^2^ = 95%) (**Figure 7**). Taken together, compared with PBC, the study results showed that *Astragalus*-containing TCM plus PBC significantly improved QOL.

### Adverse Drug Reactions (ADRs)

Twenty-nine trials that included 2,053 patients reported ADRs principally according to the WHO criteria (12) (**Table 2** and **Figures S1–10**). The results of the random-effects meta-analysis demonstrated that, compared with PBC alone, *Astragalus*-containing TCM plus PBC significantly reduced the risk of neutropenia (RR: 0.65, 95% CI: 0.54–0.79, P < 0.00001), anemia (RR: 0.68, 95% CI: 0.52–0.89, P = 0.005), thrombocytopenia (RR: 0.65, 95% CI: 0.51–0.82, P = 0.0004), nausea and vomiting (RR: 0.73, 95% CI: 0.64–0.82, P < 0.00001), diarrhea (RR: 0.58, 95% CI: 0.45–0.75, P < 0.0001), hepatic dysfunction (RR: 0.55, 95% CI: 0.40–0.77, P = 0.0005), renal dysfunction (RR: 0.48, 95% CI: 0.26–0.88, P = 0.02), neurotoxicity (RR: 0.78, 95% CI: 0.65–0.92, P = 0.004), and alopecia (RR: 0.77, 95% CI: 0.61–0.97, P = 0.03), but not the stomatitis rate (RR: 0.73, 95% CI: 0.53–1.00, P = 0.05).

**Table 2:** Meta-analysis results of adverse drug reactions (ADRs).

Outcomes	Trials	Experimental group (Events/Total)	Control group (Events/Total)	SM	RR, 95%CI	I^2^	P
Neutropenia	20	269/698	416/696	REM	0.65 [0.54, 0.79]	79%	<0.00001
Anemia	13	135/379	195/375	REM	0.68 [0.52, 0.89]	70%	0.005
Thrombocytopenia	20	155/602	237/595	REM	0.65 [0.51, 0.82]	62%	0.0004
Nausea and vomiting	19	263/647	377/646	REM	0.73 [0.64, 0.82]	32%	<0.00001
Diarrhea	10	62/307	111/301	REM	0.58 [0.45, 0.75]	0%	<0.0001
Hepatic dysfunction	14	45/442	86/446	REM	0.55 [0.40, 0.77]	0%	0.0005
Renal dysfunction	10	14/324	34/323	REM	0.48 [0.26, 0.88]	0%	0.02
Neurotoxicity	12	123/389	162/379	REM	0.78 [0.65, 0.92]	0%	0.004
Alopecia	4	50/145	61/138	REM	0.77 [0.61, 0.97]	0%	0.03
Stomatitis	6	45/174	62/174	REM	0.73 [0.53, 1.00]	0%	0.05

RR, risk ratio; CI, confidence interval; SM, statistical method; REM, random-effect model.

### Peripheral Blood Lymphocyte Levels

**Figure 8:**
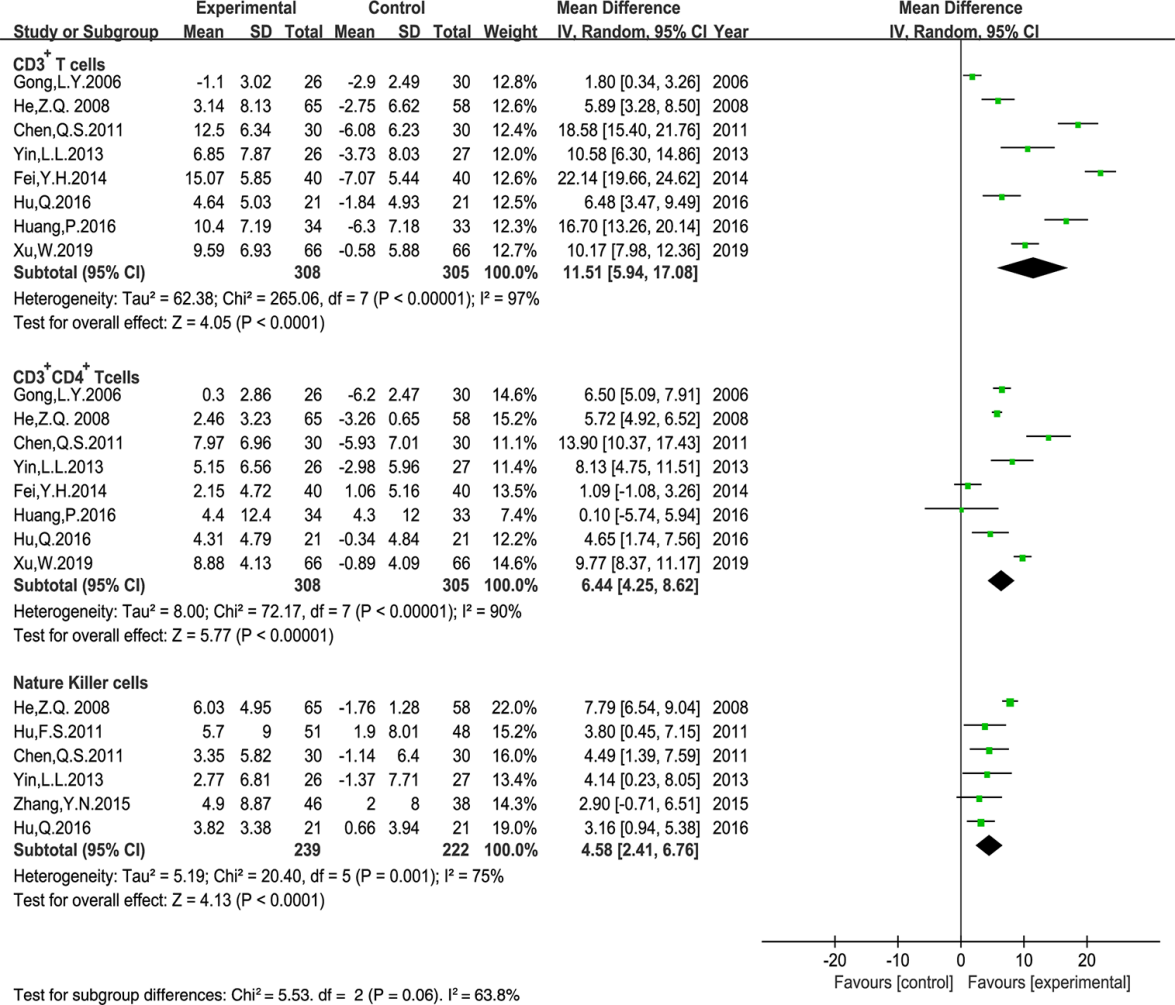
Meta-analysis results of CD3^+^ T cells, CD3^+^ CD4^+^ T cells, Nature Killer cells between the two groups.

**Figure 9:**
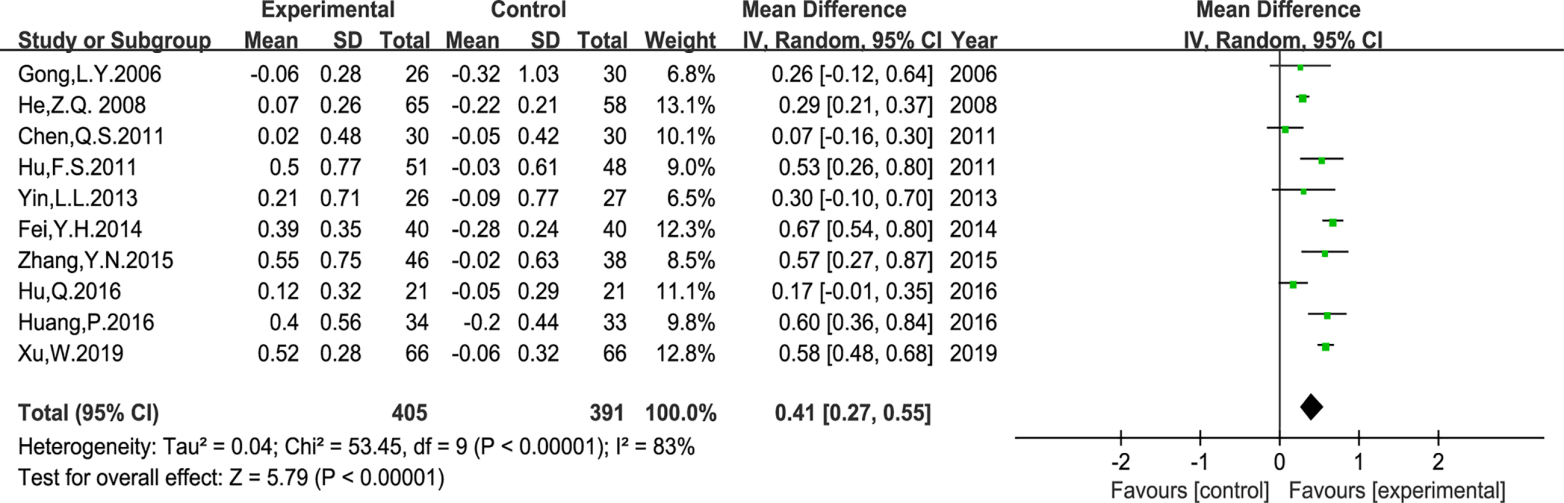
Meta-analysis results of CD4^+^/CD8^+^ T cells ratio between the two groups.

Ten trials that included 796 patients reported the peripheral blood lymphocyte levels. The results of CD3^+^ CD8^+^ T cell levels displayed poor clinical consistency. Therefore, we only evaluated the MD of the CD3^+^ T cells, CD3^+^ CD4^+^ T cells, the CD4^+^/CD8^+^ T cells ratio, and NK cells using a random-effects model. Our results indicated that *Astragalus*-containing TCM plus PBC significantly enhanced the ratio of CD3^+^ T cells (MD: 11.51, 95% CI: 5.94–17.08, P < 0.0001), CD3^+^ CD4^+^ T cells (MD: 6.44, 95% CI: 4.25–8.62, P < 0.00001), NK cells (MD: 4.58, 95% CI: 2.41–6.76, P < 0.0001) (**Figure 8**), and the CD4^+^/CD8^+^ T cells ratio (MD: 0.41, 95% CI: 0.27–0.55, P < 0.00001) (**Figure 9**) after treatment.

### Subgroup Analysis of ORR and DCR

We performed subgroup analyses in accordance with the drug delivery of *Astragalus*-containing TCM, PBC regimen, and treatment duration to reveal the influence of the addition of *Astragalus*-containing TCM on the ORR and DCR (**Figures S11–16**). The drug delivery of *Astragalus*-containing TCM was either orally or by injection. Subgroup analysis showed that *Astragalus*-containing TCM could improve the ORR and DCR regardless of whether it was administered orally or by injection, respectively (**Table 3**). PBC regimens were cisplatin-based chemotherapy and oxaliplatin-based chemotherapy regimens. Subgroup analysis displayed that *Astragalus*-containing TCM plus oxaliplatin-based chemotherapy could enhance both the ORR and DCR; however, when combined with cisplatin-based chemotherapy, it could only enhance the ORR (**Table 3**). Finally, the treatment durations were ≥8 weeks and <8 weeks. As shown in **Table 3**, whether the treatment duration was ≥8 weeks or <8 weeks, the combination of *Astragalus*-containing TCM and PBC could improve the ORR and DCR.

**Table 3:** Subgroup analysis of ORR and DCR.

Subgroups	Number of studies	RR (95% CI)	Z	P	Heterogeneity	
					I^2^	P_h_
**1.Objective response rate (ORR)**						
**Drug delivery**						
Orally	15	1.27 (1.15, 1.39)	4.93	<0.00001	0%	0.96
Injection	17	1.20 (1.07, 1.35)	3.08	0.002	0%	0.94
**PBC regimens**						
DDP-based chemotherapy regimen	10	1.25 (1.09, 1.44)	3.21	0.001	0%	0.79
OXA-based chemotherapy regimen	22	1.24 (1.13, 1.35)	4.81	<0.00001	0%	0.99
**Treatment duration**						
≥8 weeks	22	1.27 (1.15, 1.40)	4.74	<0.00001	0%	0.98
<8 weeks	10	1.21 (1.08, 1.35)	3.36	0.0008	0%	0.88
**2.Disease control rate (DCR)**						
**Drug delivery**						
Orally	15	1.14 (1.07, 1.22)	4.03	<0.0001	27%	0.16
Injection	16	1.07 (1.02, 1.12)	2.73	0.006	0%	0.65
**PBC regimens**						
DDP-based chemotherapy regimen	10	1.06 (1.00, 1.13)	2.06	0.04	0%	0.93
OXA-based chemotherapy regimen	21	1.13 (1.07, 1.20)	4.25	<0.0001	31%	0.09
**Treatment duration**						
≥8 weeks	22	1.10 (1.05, 1.16)	4.14	<0.0001	6%	0.38
<8 weeks	9	1.09 (1.02, 1.18)	2.45	0.01	28%	0.19

RR, risk ratio; CI, confidence interval; DDP, cisplatin; OXA, oxaliplatin.

### Sensitivity Analysis

We performed sensitivity analysis by omitting each study in turn to check the robustness of the main outcome, including ORR, DCR, and survival rate. This analysis showed that the pooled RR values of the ORR, DCR, and 1-year survival rate were stable, except for the half-year survival rate (**Table 4**). Considering that the half-year survival rate was extracted from survival curves in the study of Pan, B.Y.2018 (48), we excluded this study, and the I^2^ decreased from 90 to 0%.

**Table 4:** Sensitivity analysis.

Indicators	Trials	SM	RR (95% CI)	I^2^	Excluded trials	Trials	SM	RR (95% CI)	I^2^
half-year survival rate	5	REM	1.14 [0.89, 1.45]	90%	Pan, B.Y. 2018 (48)	4	REM	1.18 [1.05, 1.33]	0%

SM, statistical method; REM, random-effects model; RR, risk ratio; CI, confidence interval.

As shown in **Figures S17, 18**, the TSA for the ORR and DCR showed that the Z-curve (blue dashed line) crossed the conventional boundary (brown dotted line), the trial sequential monitoring boundary for benefit (lower red solid line), and the RIS of the TSA (vertical red line; 660 for ORR, 574 for DCR), which suggested that the findings of the meta-analysis were robust for the ORR and DCR.

The TSA for the half-year survival rate (**Figure S19**) showed that the Z-curve did not cross either the conventional or trial sequential monitoring boundary, as well as the RIS (equal to 2920), which indicated that the evidence on the effect of half-year survival rate was insufficient. TSA for 1-year survival rate (**Figure S20**) showed that the Z-curve crossed the conventional and trial sequential monitoring boundary for benefit, but not the RIS (equal to 611). This indicates that the combination of *Astragalus*-containing TCM and PBC might enhance the 1-year survival rate for AGC patients. However, we did not have enough power to confirm the conclusion before RIS of 611 participants. The TSA for the 2-year survival rate (**Figure S21**) showed that the Z-curve crossed the conventional boundary and RIS (equal to 95), which indicated that the evidence on the effect of the 2-year survival rate was robust. Due to insufficient data, we failed to evaluate the effect on the 3-year survival rate.

### Publication Bias

The funnel plots (**Figures S22–33**) were not strictly symmetrical in the meta-analysis of the ORR, DCR, QOL, neutropenia, anemia, thrombocytopenia, nausea and vomiting, diarrhea, hepatic dysfunction, renal dysfunction, neurotoxicity, and CD4^+^/CD8^+^ T cells ratio. However, as shown in Egger’s test (**Table 5**), no significant publication bias existed among the meta-analysis of ORR (P = 0.3986), QOL (P = 0.7377), diarrhea (P = 0.3092), hepatic dysfunction (P = 0.5113), renal dysfunction (P = 0.9436), and CD4^+^/CD8^+^ T cells ratio (P=0.5250).

**Table 5:** Egger’s test.

Meta-analysis of publication bias	P value
ORR	0.3986
DCR	0.0021
QOL	0.7377
Neutropenia	0.0000
Anemia	0.0487
Thrombocytopenia	0.0000
Nausea and vomiting	0.0002
Diarrhea	0.3092
Hepatic dysfunction	0.5113
Renal dysfunction	0.9436
Neurotoxicity	0.0388
CD4^+^/CD8^+^ T cells ratio	0.5250

ORR, objective response rate; DCR, disease control rate; QOL, quality of life.

### Quality of Evidence

In summary, the quality was moderate for the ORR, 1-year survival rate, QOL, hepatic dysfunction, renal dysfunction, stomatitis, and levels of peripheral blood lymphocytes; low for DCR, neutropenia, anemia, thrombocytopenia, nausea and vomiting, diarrhea, and neurotoxicity; and very low for other results (**Table 6**).

**Table 6:** GRADE evidence profile.

TABLE 6a | GRADE evidence profile of clinical efficacy and safety.									
Outcomes (Trials)	Quality assessment					No. of patients		Risk ratios (95% CI)	Quality
	Risk of bias	Inconsistency	Indirectness	Imprecision	Reporting bias	*Astragalus*-Containing TCM	PBC		
ORR (32)	Serious^a^	No	No	No	No	666/1,235 (53.9%)	524/1,219 (43%)	RR 1.24 (1.15 to 1.34)	⊕⊕⊕O
									Moderate
DCR (32)	Serious^a^	No	No	No	Serious	1,021/1,187 (86%)	893/1,171 (76.3%)	RR 1.10 (1.06 to 1.14)	⊕⊕OO
									Low
Half-year survival rate (5)	Very Serious^d^	Serious^g^	No	No	No	152/160 (95%)	132/155 (85.2%)	RR 1.14 (0.89 to 1.45)	⊕OOO
									Very Low
1-year survival rate (8)	Serious^c^	No^f^	No	No	No	173/254 (68.1%)	127/258 (49.2%)	RR 1.41 (1.09 to 1.82)	⊕⊕⊕O
									Moderate
2-year survival rate (3)	Very Serious^d^	No	No	Serious^e^	No	43/121 (35.5%)	13/117 (11.1%)	RR 3.13 (1.80 to 5.46)	⊕OOO
									Very Low
3-year survival rate (1)	Very Serious^d^	No	No	Serious^e^	No	0/58 (0%)	0/54 (0%)	Not pooled	⊕OOO
									Very Low
QOL, according to the number of KPS improved patients (14)	Serious^c^	No	No	No	No	233/426 (54.7%)	108/413 (26.2%)	RR 2.03 (1.70 to 2.43)	⊕⊕⊕O
									Moderate
Neutropenia (21)	Serious^a^	No^f^	No	No	Serious	269/698 (38.5%)	416/696 (59.8%)	RR 0.65 (0.54 to 0.79)	⊕⊕OO
									Low
Anemia (13)	Serious^a^	No^f^	No	No	Serious	135/379 (35.6%)	195/375 (52%)	RR 0.68 (0.52 to 0.89)	⊕⊕OO
									Low
Thrombocytopenia (21)	Serious^a^	No^f^	No	No	Serious	155/602 (25.7%)	237/595 (39.8%)	RR 0.65 (0.51 to 0.82)	⊕⊕OO
									Low
Nausea and vomiting (20)	Serious^a^	No^f^	No	No	Serious	263/647 (40.6%)	377/646 (58.4%)	RR 0.73 (0.64 to 0.82)	⊕⊕OO
									Low
Diarrhea (10)	Serious^c^	No	No	No	Serious	62/307 (20.2%)	111/301 (36.9%)	RR 0.58 (0.45 to 0.75)	⊕⊕OO
									Low
Hepatic dysfunction (14)	Serious^a^	No	No	No	No	45/442 (10.2%)	86/446 (19.3%)	RR 0.55 (0.40 to 0.77)	⊕⊕⊕O
									Moderate
Renal dysfunction (10)	Serious^a^	No	No	No	No	14/324 (4.3%)	34/323 (10.5%)	RR 0.48 (0.26 to 0.88)	⊕⊕⊕O
									Moderate
Neurotoxicity (12)	Serious^a^	No	No	No	Serious	123/389 (31.6%)	162/379 (42.7%)	RR 0.78 (0.65 to 0.92)	⊕⊕OO
									Low
Alopecia (4)	Very Serious^d^	No	No	Serious^e^	No	50/145 (34.5%)	61/138 (44.2%)	RR 0.77 (0.61 to 0.97)	⊕OOO
									Very Low
Stomatitis (6)	Serious^a^	No	No	No	No	45/174 (25.9%)	62/174 (35.6%)	RR 0.73 (0.53 to 1.00)	⊕⊕⊕O
									Moderate

ORR, objective response rate; DCR, disease control rate; QOL, quality of life; NK cells, natural killer cells; CI, confidence interval.

a Most trials had unclear risk, and with high risk, but the result had good robustness. The evidence was rated down by only one level.

b Most trials had unclear risk and with high risk, and the result had poor robustness. The evidence was rated down by two levels.

c Most trials had unclear risk and the trials were no high risk, but the result had good robustness. The evidence was rated down by only one level.

d Most trials had unclear risk and the trials were no high risk, but the result had poor robustness. The evidence was rated down by two levels.

e The sample size for each outcome was fewer than 300 cases. Therefore, the evidence was rated down by one level.

f Heterogeneity presented in them, and the results had good robustness. Not rated down.

g Heterogeneity presented in them, and the result had poor robustness. The evidence was rated down by one level.

**Table 6:** GRADE evidence profile.

**TABLE 6b | GRADE evidence profile of QOL (mean ± SD) and levels of peripheral blood lymphocytes.**									
**Outcomes (Trials)**	**Quality assessment**					**No. of patients**		**Mean difference (95% CI)**	**Quality**
	**Risk of bias**	**Inconsistency**	**Indirectness**	**Imprecision**	**Reporting bias**	***Astragalus*-Containing TCM**	**PBC**		
QOL, according to mean ± SD (6)	Serious^a^	No^f^	No	No	No	167	162	MD 12.39 higher (5.48 to 19.3 higher)	⊕⊕⊕O
									Moderate
CD3^+^ T cells (8)	Serious^a^	No^f^	No	No	No	308	305	MD 11.51 higher (5.94 to 17.08 higher)	⊕⊕⊕O
									Moderate
CD3^+^CD4^+^ T cells (8)	Serious^a^	No^f^	No	No	No	308	305	MD 6.44 higher (4.25 to 8.62 higher)	⊕⊕⊕O
									Moderate
CD4^+^/CD8^+^ T cells ratio (10)	Serious^a^	No^f^	No	No	No	405	391	MD 0.41 higher (0.27 to 0.55 higher)	⊕⊕⊕O
									Moderate
NK cells (6)	Serious^a^	No^f^	No	No	No	239	222	MD 4.58 higher (2.41 to 6.76 higher)	⊕⊕⊕O
									Moderate

ORR, objective response rate; DCR, disease control rate; QOL, quality of life; NK cells, natural killer cells; CI, confidence interval.

a Most trials had unclear risk, and with high risk, but the result had good robustness. The evidence was rated down by only one level.

b Most trials had unclear risk and with high risk, and the result had poor robustness. The evidence was rated down by two levels.

c Most trials had unclear risk and the trials were no high risk, but the result had good robustness. The evidence was rated down by only one level.

d Most trials had unclear risk and the trials were no high risk, but the result had poor robustness. The evidence was rated down by two levels.

e The sample size for each outcome was fewer than 300 cases. Therefore, the evidence was rated down by one level.

f Heterogeneity presented in them, and the results had good robustness. Not rated down.

g Heterogeneity presented in them, and the result had poor robustness. The evidence was rated down by one level.

## Discussion

Gastric cancer has high incidence and morbidity levels around the world. In China, patients with AGC have poor clinical outcomes and a low 5-year survival rate of <20% (53). PBC is widely used and plays a crucial role in the treatment of AGC (54). However, the survival benefit in patients with AGC is still limited; therefore, it is necessary to develop effective combination therapies to increase the efficacy of PBC and reduce side effects in order to prolong the survival time and improve QOL in these patients.

As an important part of the comprehensive treatment of cancer, TCM, especially *Astragalus*-containing TCM, is used in combination with chemotherapy and broadly prescribed for AGC patients in China. To evaluate whether *Astragalus*-containing TCM with PBC improves the clinical efficacy and its safety, 35 RCTs involving 2,670 patients with AGC were included in this meta-analysis. To the best of our knowledge, it is the first systematic review and meta-analysis that has evaluated the efficacy and safety of *Astragalus*-containing TCM in combination with PBC for AGC treatment.

Our results indicated that *Astragalus*-containing TCM could enhance the ORR and DCR of PBC, meaning that the experimental group had a better short-term efficacy. Furthermore, we also performed subgroup analyses according to the drug delivery method of *Astragalus*-containing TCM, PBC regimen, and treatment duration. The results suggested that compared with PBC alone, both oral and injection-based administration of *Astragalus*-containing TCM resulted in better tumor response in AGC patients. In addition, both *Astragalus*-containing TCM combined with cisplatin-based chemotherapy and oxaliplatin-based chemotherapy resulted in a better ORR than chemotherapy alone. AGC patients that were given *Astragalus*-containing TCM plus oxaliplatin-based chemotherapy had better DCR; however, those who were given *Astragalus*-containing TCM plus cisplatin-based chemotherapy showed no difference in DCR. In addition, the subgroup analysis results also showed that *Astragalus*-containing TCM plus PBC resulted in better tumor response in AGC patients regardless of the duration of administration. Moreover, basic studies also showed that *Astragalus* and its main components could reduce or stabilize the gastric tumor by inducing antiproliferation, promoting apoptosis, and modulating the invasiveness of tumor cells, among other mechanisms (55–57). These results provided indirect basic and mechanistic evidences for the antitumor mechanisms of using *Astragalus*-containing TCM in AGC.

In terms of the survival rate, our study indicated that *Astragalus*-containing TCM plus PBC enhanced the 1- (P = 0.009) and 2-year survival rates (P < 0.0001) compared with PBC alone. However, due to the small number of included trials (n = 8), the data available for subgroup and sensitivity analyses were limited. In addition, none of the included studies completely reported the overall survival, progression-free survival, 5-year survival rate, which were important clinical survival outcomes. Therefore, conducting a meta-analysis on these outcome measures was not possible. More evidence is needed to support our findings.

PBC often leads to many adverse drug reactions in patients with AGC, which seriously impacts QOL. Therefore, reducing chemotherapy side effects while maintaining the curative effect has become a popular and urgent topic. More and more evidence has shown that *Astragalus* and its main components (such as *Astragalus* polysaccharide, flavonoid compounds, saponins compounds, alkaloids, etc.) have the potential to reduce side effects of chemotherapeutic agents (58–61). According to our results, *Astragalus*-containing TCM plus PBC reduced ADRs (bone marrow suppression, gastrointestinal reaction, hepatic and renal dysfunction, neurotoxicity, and alopecia) in patients with stage III/IV AGC (P < 0.05) and brought significantly improvement on their QOL based on KPS.

The human immune system plays a vital role in immune surveillance of malignant cells. Patients with AGC have low immune function, and PBC often aggravates the immunosuppressive state in patients, impairing the antitumor response (62), which increases the risk of tumor invasion and metastasis. Therefore, improving immunity is of great significance in antitumor therapy. An increasing number of pharmacological studies have shown that *Astragalus* and its ingredients could improve chemotherapy-induced immunosuppression, which is associated with protective effects on immune organs by regulating leukocytes, lymphocytes, and macrophages, among other cells (63–65). In clinical settings, determination of peripheral blood lymphocytes is an effective method for evaluating the immune function of patients. This meta-analysis showed that *Astragalus*-containing TCM plus PBC significantly increased the percentages of CD3^+^, CD3^+^CD4^+^, CD4^+^/CD8^+^, and NK cells in the peripheral blood.

This study has several limitations. Firstly, we only searched for literature from Chinese and English databases; thus, some related studies from Japanese, Korean, or other databases might have been missed. All of the RCTs included in our study were conducted in China, and only one study was published in an international journal (48); Funnel plots and egger’s test results reflected that there was some potential publication bias. Secondly, only nine studies clearly described the use of randomization methods. In most included trials, allocation concealment and blinding were “unclear”, which might result in potential implementation bias and selective bias. Thirdly, in all included trials, the follow-up duration was relatively short (≤3 years). We look forward to more longer-term follow-up studies to support our findings. Fourthly, there was substantial heterogeneity (I^2^ > 50%) among studies for many outcomes, such as the half- and 1-year survival rate, peripheral blood lymphocyte levels, neutropenia, anemia, and thrombocytopenia, among others, which might have weakened the evidence strength of this meta-analysis. Therefore, more well-designed RCTs are needed to support our findings. Fifthly, in the treatment of AGC, *Astragalus* is rarely used as single-agent therapy; it usually is combined with other different herbal medicines. Additional researches are needed to further understand the specific immunological and cytotoxic mechanisms of *Astragalus* as an adjuvant to chemotherapy in the treatment of AGC. And lastly, based on the GRADE approach, the quality of outcomes was moderate to very low; most trials we included in our manuscript might not be reported strictly in accordance with the CONSORT reporting standards. All of these limitations might have resulted in insufficient evaluation of the outcome. However, we hope that our manuscript will make more and more doctors realize the potential benefits of *Astragalus*-containing TCM in AGC, and conduct more well-designed clinical trials adhering to CONSORT guidelines to verify the efficacy of *Astragalus*-containing TCM in the future.

## Conclusion

Our study demonstrated the potencies of *Astragalus*-containing TCM with PBC to enhance the efficacy and safety for patients with AGC, and more efforts are needed to promote the application of *Astragalus*-containing TCM in the clinic. Furthermore, the long-term efficacy of *Astragalus*-containing TCM plus PBC in AGC treatment still needs to be verified in future well-designed clinical trials that adhere to CONSORT guidelines.

## Data Availability Statement

The original contributions presented in the study are included in the article/**Supplementary Material**. Further inquiries can be directed to the corresponding authors.

## Author Contributions

BH and YQ designed the research. MC, JH, and YZ performed literature search. YL and JJ performed article selection. RL and QG assessed methodological bias risk. XZ and YQ performed data extraction. JJ, RQ, SC, and HZ conducted a meta-analysis and assessed study quality. MC finished the manuscript draft. All authors contributed to the article and approved the submitted version.

## Funding

This study was supported by the National Natural Science Foundation of China (Grant Nos. 81673961, 81774294), Beijing Municipal Science and Technology Commission (No. Z181100001618006), Beijing Natural Science Foundation (No. 7172186), Institution Subject of Guang’anmen Hospital, China Academy of Chinese Medical Sciences (Grant No. 2019S452).

## Conflict of Interest

The authors declare that the research was conducted in the absence of any commercial or financial relationships that could be construed as a potential conflict of interest.

## Publisher’s Note

All claims expressed in this article are solely those of the authors and do not necessarily represent those of their affiliated organizations, or those of the publisher, the editors and the reviewers. Any product that may be evaluated in this article, or claim that may be made by its manufacturer, is not guaranteed or endorsed by the publisher.

## Abbreviations

TCM, traditional Chinese medicine; AGC, advanced gastric cancer; PBC, platinum-based chemotherapy; RCTs, randomized controlled trials; RR, risk ratio; MD, mean difference; CI, confidence interval; TSA, trial sequential analysis; GRADE, grading of recommendations assessment, development, and evaluation; RIS, required information size; ORR, objective response rate; DCR, disease control rate; QOL, quality of life; ADRs, adverse drug reactions.
