# Supplementary material for: Efficacy and Safety of Astragalus-Containing Traditional Chinese Medicine Combined With Platinum-Based Chemotherapy in Advanced Gastric Cancer: A Systematic Review and Meta-Analysis
Source: Front Oncol. 2021 Aug 4;11:632168. doi: 10.3389/fonc.2021.632168 (PMC8371531; doi:10.3389/fonc.2021.632168)
Supplement: Supplementary file 1 [file DataSheet_1.pdf]

Supplementary Material

1 Supplementary Data

Search Strategy Used in Pubmed 2020/7/19

#1 Stomach Neoplasms [Mesh]
#2 (gastr*[Title/Abstract] OR stomach*[Title/Abstract] OR digest*[Title/Abstract] OR epigastr*[Title/Abstract]) AND (carcin*[Title/Abstract] OR cancer*[Title/Abstract] OR neoplas*[Title/Abstract] OR tumour*[Title/Abstract] OR tumor*[Title/Abstract] OR growth*[Title/Abstract] OR adenocarcin*[Title/Abstract] OR malig*[Title/Abstract])
#3 #1 OR #2
#4 Astragalus propinquus [Mesh]
#5 "astragalus"[Text Word] OR "radix astragali"[Text Word] OR "huang qi"[Text Word] OR "huangqi"[Text Word]
#6 kangai[Title/Abstract] OR kang-ai[Title/Abstract] OR aidi[Title/Abstract] OR ai-di[Title/Abstract] OR shenqi*[Title/Abstract] OR delisheng[Title/Abstract] OR de-li-sheng[Title/Abstract] OR qizhen[Title/Abstract] OR boerning[Title/Abstract] OR compound banmao[Title/Abstract] OR zhenqi[Title/Abstract] OR yangzheng xiaoji[Title/Abstract] OR qijiao shengbai[Title/Abstract] OR lianqi[Title/Abstract] OR cidan [Title/Abstract] OR compound tianxian[Title/Abstract] OR buzhong yiqi[Title/Abstract] OR shiquan dabu[Title/Abstract] OR guipi[Title/Abstract] OR renshen yangrong[Title/Abstract] OR danggui buxue[Title/Abstract]
#7 #4 OR #5 OR #6
#8 #3 AND #7

2 Supplementary Figures and Tables

2.1 Supplementary Figures

Study or Subgroup	Experimental Events	Experimental Total	Control Events	Control Total	Weight	Risk Ratio M-H, Random, 95% CI	Year
Yang.J.2005	13	16	14	16	6.9%	0.93 [0.69, 1.25]	2005
Gong,L.Y.2006	25	26	30	30	8.1%	0.96 [0.87, 1.06]	2006
Li,A.M.2007	15	64	31	64	5.2%	0.48 [0.29, 0.81]	2007
Wang.L.2007	10	15	12	15	5.8%	0.83 [0.54, 1.29]	2007
Chen,P.2007	14	64	31	64	5.1%	0.45 [0.27, 0.77]	2007
He,Z.Q. 2008	21	65	30	58	5.9%	0.62 [0.41, 0.96]	2008
Liu,L.H.2009	19	30	25	30	6.8%	0.76 [0.55, 1.04]	2009
Fan,C.M.2011	10	23	15	28	4.7%	0.81 [0.45, 1.45]	2011
Chen,Q.S.2011	8	30	22	30	4.4%	0.36 [0.19, 0.68]	2011
Hu,F.S.2011	15	51	31	48	5.5%	0.46 [0.28, 0.73]	2011
Wang,J.R.2012	20	26	23	24	7.5%	0.80 [0.64, 1.01]	2012
Yin,L.L.2013	5	26	12	27	3.0%	0.43 [0.18, 1.06]	2013
Xiang,S.L.2014	1	33	2	33	0.6%	0.50 [0.05, 5.25]	2014
Zhang,L.2014	23	32	28	32	7.3%	0.82 [0.64, 1.06]	2014
Ma,M.2016	4	22	11	22	2.6%	0.36 [0.14, 0.97]	2016
Hu,Q.2016	9	21	13	21	4.6%	0.69 [0.38, 1.26]	2016
Duan,F.2016	1	46	4	46	0.7%	0.25 [0.03, 2.15]	2016
Hu,Q.2017	6	18	12	18	3.8%	0.50 [0.24, 1.04]	2017
Yuan,d.d.2019	43	60	52	60	7.7%	0.83 [0.69, 1.00]	2019
Zhu,Y.F.2019	7	30	18	30	3.9%	0.39 [0.19, 0.79]	2019
**Total (95% CI)**		**698**		**696**	**100.0%**	**0.65 [0.54, 0.79]**	
Total events	269		416				

Heterogeneity: $Tau^2 = 0.11$; $Chi^2 = 92.35$, $df = 19$ ($P < 0.00001$); $I^2 = 79\%$

Test for overall effect: $Z = 4.42$ ($P < 0.00001$)

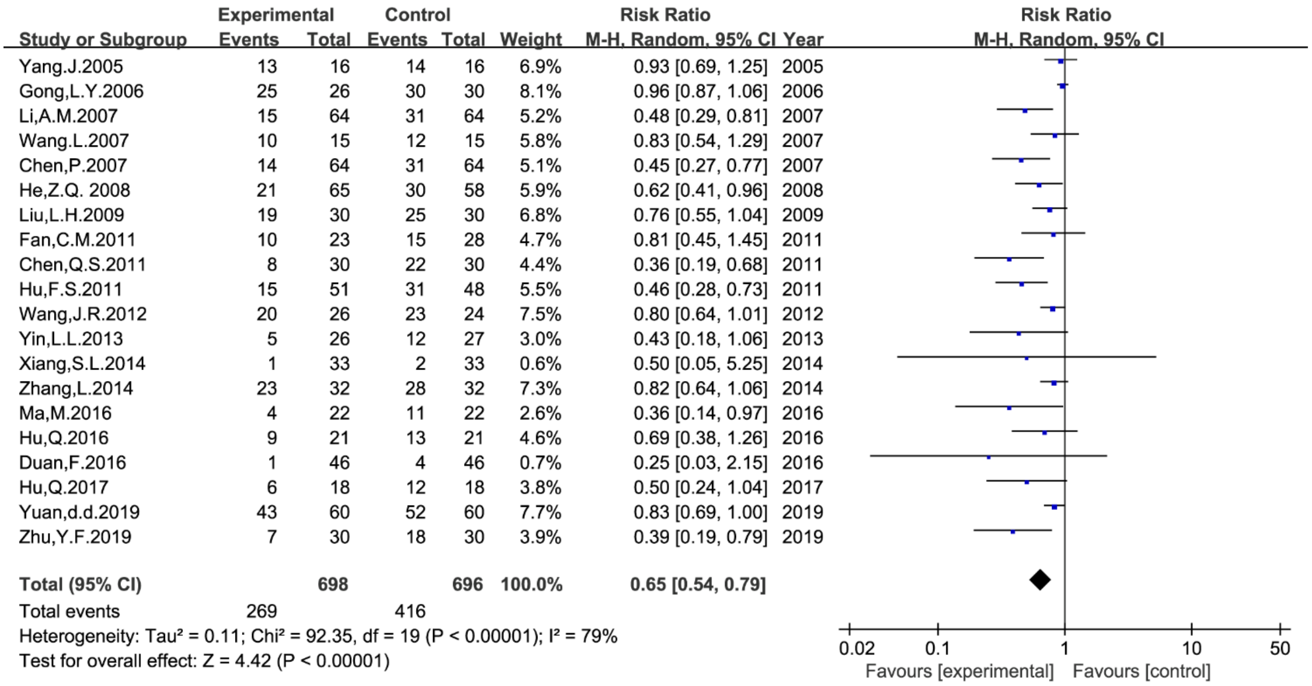

Figure S1. Meta-analysis results of neutropenia between the two groups

Study or Subgroup	Experimental Events	Experimental Total	Control Events	Control Total	Weight	Risk Ratio M-H, Random, 95% CI	Year
Yang.J.2005	10	16	12	16	10.1%	0.83 [0.52, 1.34]	2005
Gong,L.Y.2006	24	26	29	30	14.5%	0.95 [0.84, 1.09]	2006
Wang.L.2007	10	15	11	15	10.2%	0.91 [0.57, 1.45]	2007
He,Z.Q. 2008	14	65	25	58	9.1%	0.50 [0.29, 0.87]	2008
Liu,X.Q.2009	6	30	8	30	5.3%	0.75 [0.30, 1.90]	2009
Chen,Q.S.2011	11	30	24	30	9.7%	0.46 [0.28, 0.76]	2011
Hu,F.S.2011	33	51	35	48	13.0%	0.89 [0.68, 1.16]	2011
Fan,C.M.2011	3	23	5	28	3.2%	0.73 [0.19, 2.74]	2011
Yin,L.L.2013	3	26	2	27	2.1%	1.56 [0.28, 8.58]	2013
Hu,Q.2016	5	21	8	21	5.2%	0.63 [0.24, 1.60]	2016
Shao,K.F.2016	8	28	14	24	7.6%	0.49 [0.25, 0.96]	2016
Hu,Q.2017	5	18	12	18	6.2%	0.42 [0.18, 0.94]	2017
Zhu,Y.F.2019	3	30	10	30	3.7%	0.30 [0.09, 0.98]	2019
**Total (95% CI)**		**379**		**375**	**100.0%**	**0.68 [0.52, 0.89]**	
Total events	135		195				

Heterogeneity: $Tau^2 = 0.12$; $Chi^2 = 39.45$, $df = 12$ ($P < 0.0001$); $I^2 = 70\%$

Test for overall effect: $Z = 2.84$ ($P = 0.005$)

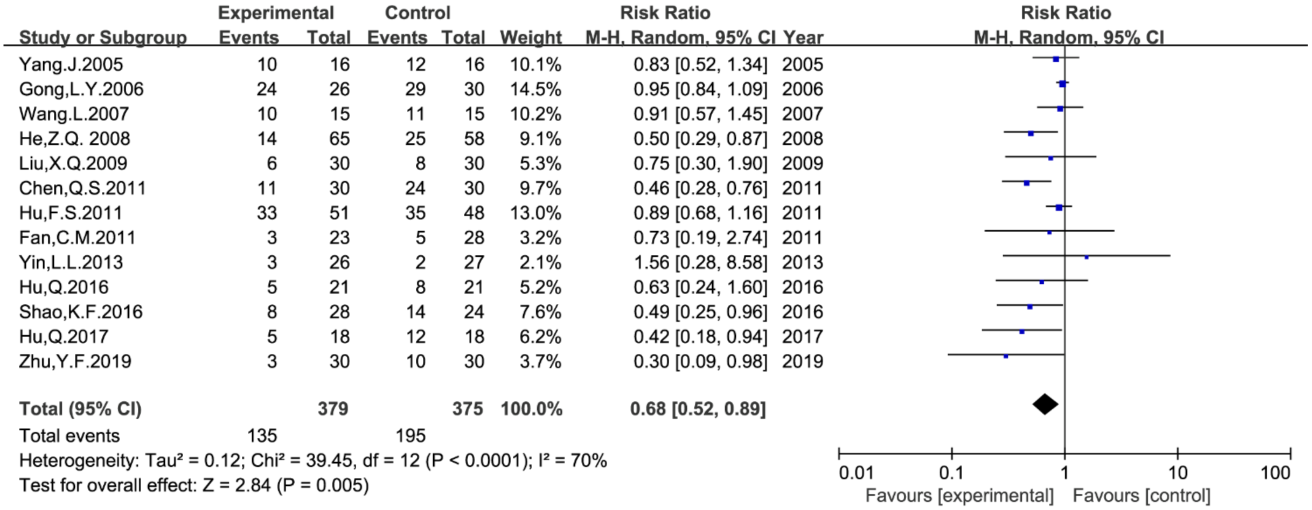

Figure S2. Meta-analysis results of anemia between the two groups

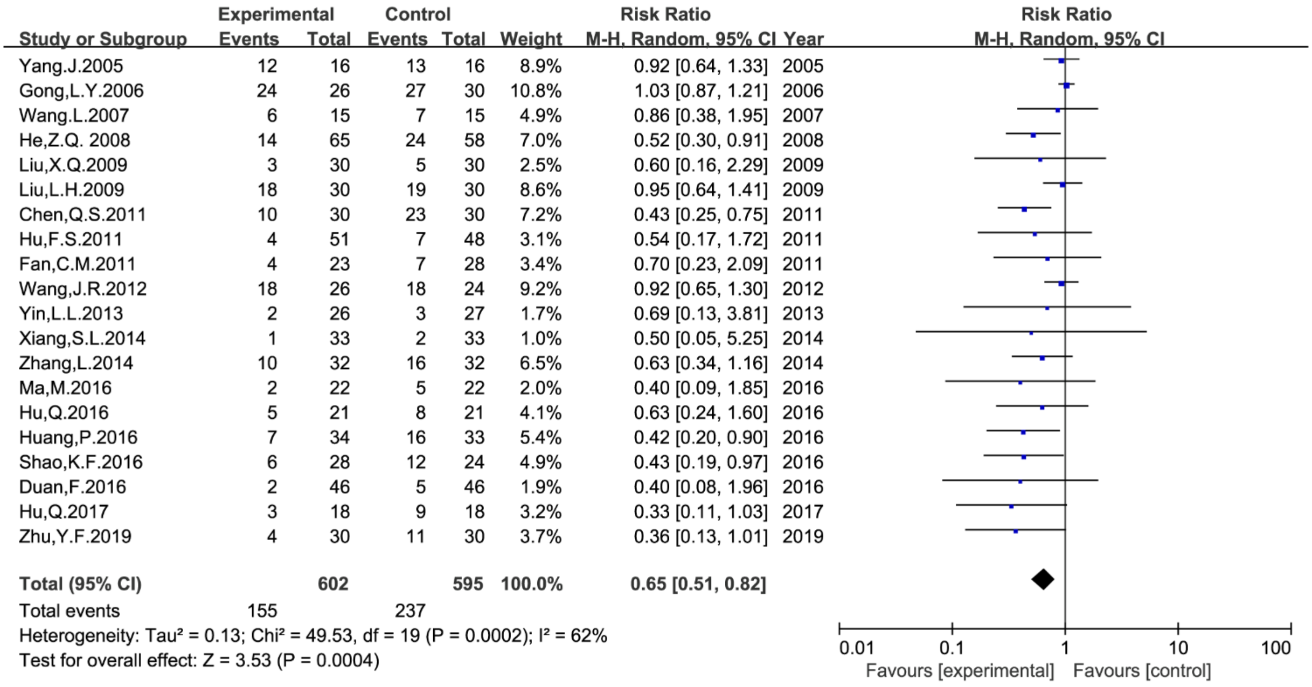

Figure S3. Meta-analysis results of thrombocytopenia between the two groups

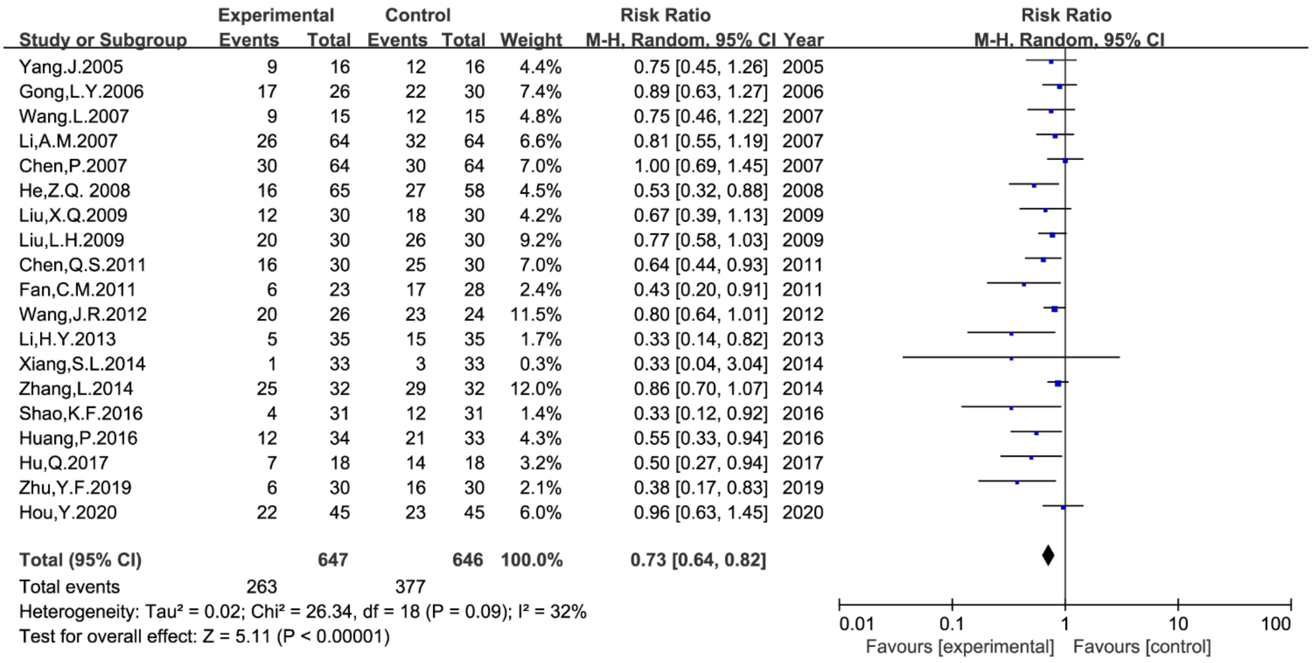

Figure S4. Meta-analysis results of nausea and vomiting between the two groups

Study or Subgroup	Experimental Events	Experimental Total	Control Events	Control Total	Weight	Risk Ratio M-H, Random, 95% CI	Year
Yang.J.2005	8	16	11	16	18.0%	0.73 [0.40, 1.31]	2005
Gong,L.Y.2006	7	26	7	30	7.6%	1.15 [0.47, 2.86]	2006
Wang.L.2007	7	15	11	15	16.3%	0.64 [0.34, 1.18]	2007
He,Z.Q. 2008	14	65	23	58	19.9%	0.54 [0.31, 0.95]	2008
Liu,X.Q.2009	2	30	4	30	2.4%	0.50 [0.10, 2.53]	2009
Chen,Q.S.2011	4	30	12	30	6.1%	0.33 [0.12, 0.92]	2011
Wang,J.R.2012	6	26	7	24	7.1%	0.79 [0.31, 2.02]	2012
Li,H.Y.2013	2	35	9	35	3.0%	0.22 [0.05, 0.96]	2013
Huang,P.2016	9	34	18	33	15.3%	0.49 [0.26, 0.92]	2016
Zhu,Y.F.2019	3	30	9	30	4.3%	0.33 [0.10, 1.11]	2019
**Total (95% CI)**		**307**		**301**	**100.0%**	**0.58 [0.45, 0.75]**	
Total events	62		111				

Heterogeneity: Tau² = 0.00; Chi² = 7.51, df = 9 (P = 0.58); I² = 0%
Test for overall effect: Z = 4.26 (P < 0.0001)

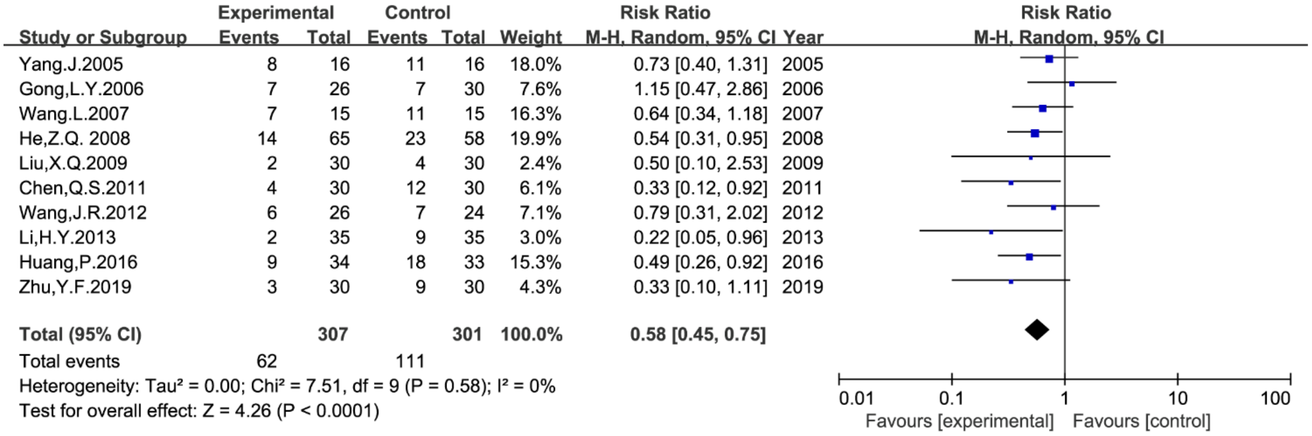

Figure S5. Meta-analysis results of diarrhea between the two groups

Study or Subgroup	Experimental Events	Experimental Total	Control Events	Control Total	Weight	Risk Ratio M-H, Random, 95% CI	Year
Gong,L.Y.2006	6	26	8	30	13.3%	0.87 [0.35, 2.17]	2006
Wang.L.2007	1	15	2	15	2.1%	0.50 [0.05, 4.94]	2007
Liu,L.H.2009	4	30	6	30	8.3%	0.67 [0.21, 2.13]	2009
Chen,Q.S.2011	3	30	9	30	7.7%	0.33 [0.10, 1.11]	2011
Hu,F.S.2011	5	51	5	48	8.1%	0.94 [0.29, 3.05]	2011
Fan,C.M.2011	1	23	3	28	2.3%	0.41 [0.05, 3.64]	2011
Wang,J.R.2012	6	26	6	24	11.5%	0.92 [0.34, 2.47]	2012
Li,H.Y.2013	2	35	9	35	5.3%	0.22 [0.05, 0.96]	2013
Zhang,M.J.2014	6	48	7	48	10.9%	0.86 [0.31, 2.36]	2014
Xiang,S.L.2014	2	33	3	33	3.8%	0.67 [0.12, 3.73]	2014
Zhang,L.2014	6	32	17	32	17.9%	0.35 [0.16, 0.78]	2014
Hu,Q.2017	2	18	8	18	5.7%	0.25 [0.06, 1.02]	2017
Zhu,Y.F.2019	1	30	2	30	2.0%	0.50 [0.05, 5.22]	2019
Hou,Y.2020	0	45	1	45	1.1%	0.33 [0.01, 7.97]	2020
**Total (95% CI)**		**442**		**446**	**100.0%**	**0.55 [0.40, 0.77]**	
Total events	45		86				

Heterogeneity: Tau² = 0.00; Chi² = 8.49, df = 13 (P = 0.81); I² = 0%
Test for overall effect: Z = 3.46 (P = 0.0005)

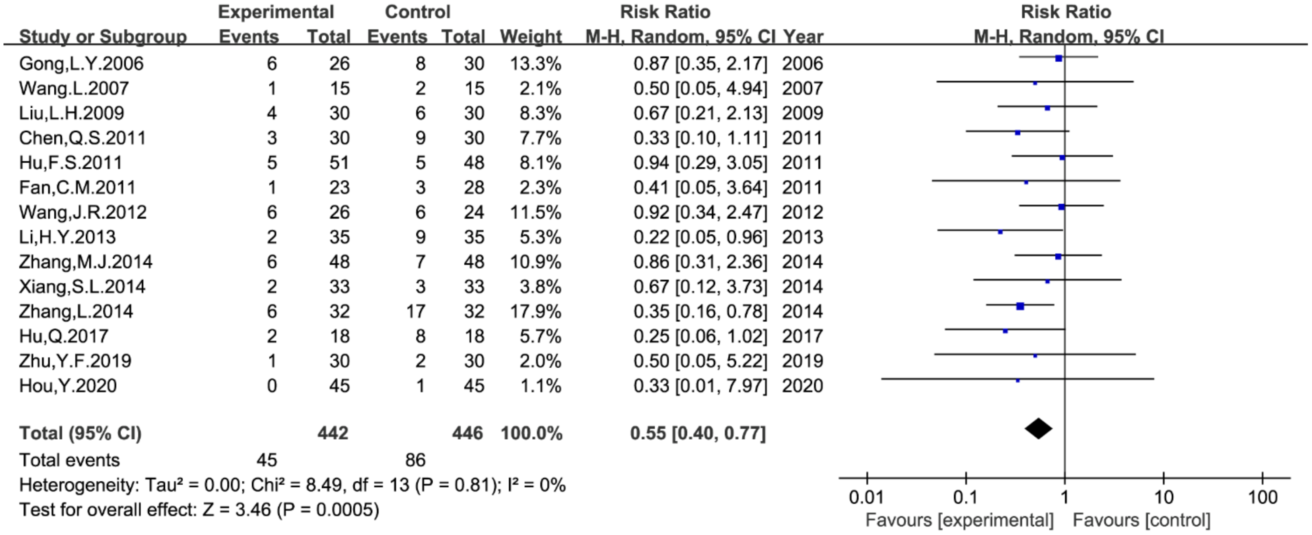

Figure S6. Meta-analysis results of hepatic dysfunction between the two groups

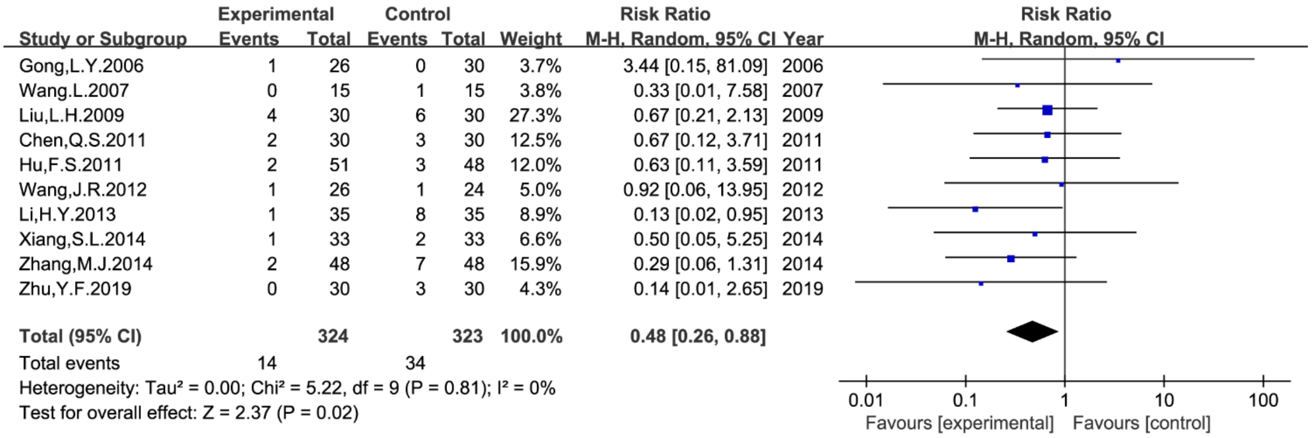

Figure S7. Meta-analysis results of renal dysfunction between the two groups

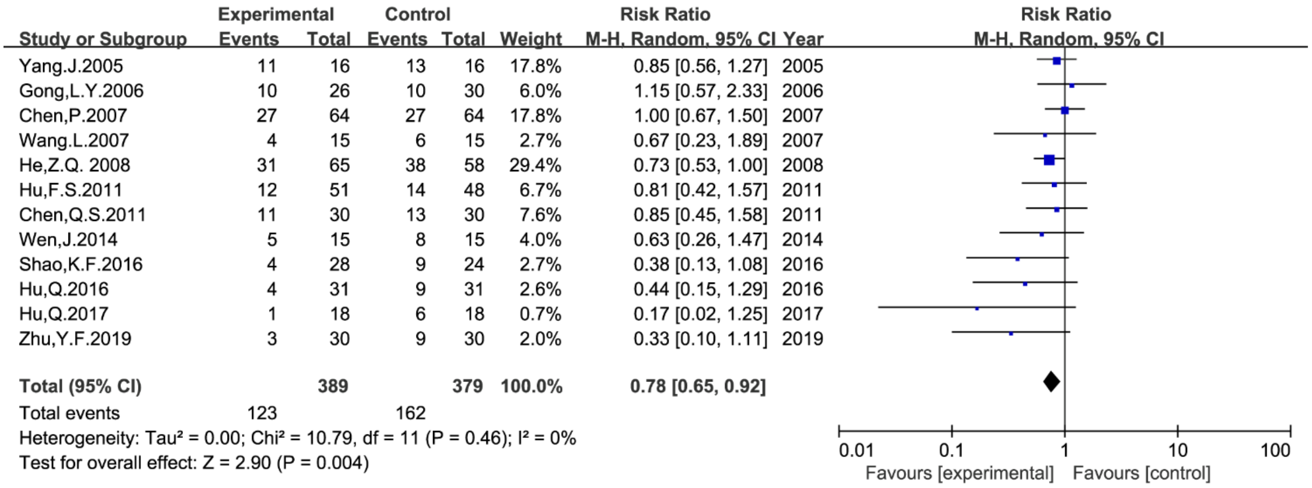

Figure S8. Meta-analysis results of neurotoxicity between the two groups

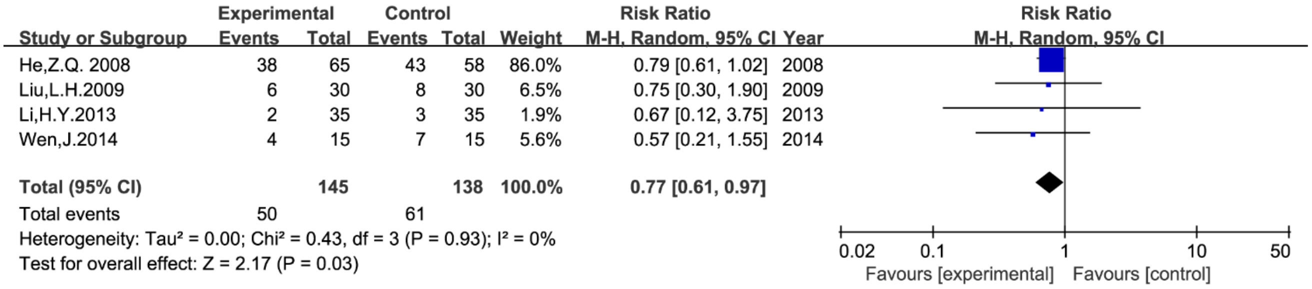

Figure S9. Meta-analysis results of alopecia between the two groups

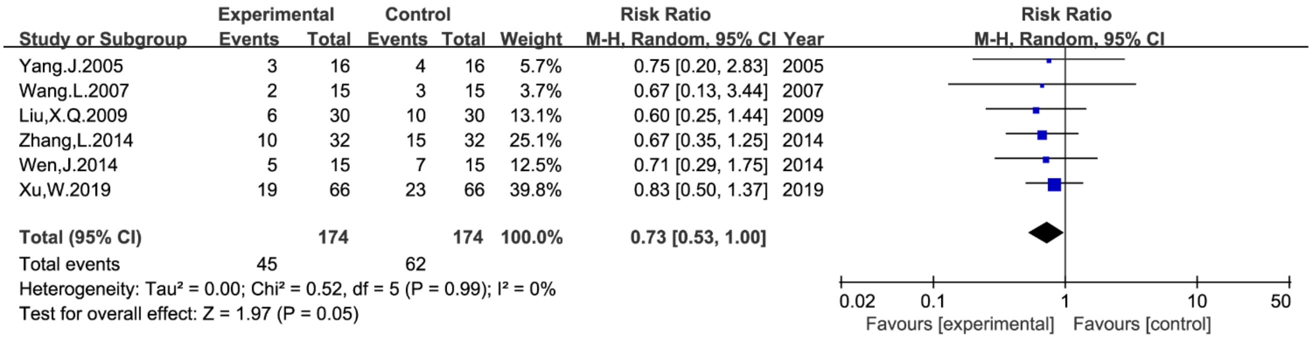

Figure S10. Meta-analysis results of stomatitis between the two groups

Study or Subgroup	Experimental Events	Experimental Total	Control Events	Control Total	Weight	Risk Ratio M-H, Random, 95% CI	Year
**Objective Response Rate (Oral Administration Group)**							
Yang.J.2005	7	16	6	16	0.8%	1.17 [0.50, 2.71]	2005
Li,A.M.2007	44	64	34	64	6.7%	1.29 [0.97, 1.72]	2007
Wang.L.2007	6	15	5	15	0.6%	1.20 [0.47, 3.09]	2007
Chen,P.2007	42	64	34	64	6.4%	1.24 [0.92, 1.65]	2007
Zhu,Y.2010	11	20	9	20	1.4%	1.22 [0.65, 2.29]	2010
Chen,Q.S.2011	6	30	2	30	0.2%	3.00 [0.66, 13.69]	2011
Du,C.J.2011	35	120	34	120	3.4%	1.03 [0.69, 1.53]	2011
Hu,F.S.2011	16	51	12	48	1.3%	1.25 [0.66, 2.37]	2011
Tan,G.2013	14	26	10	22	1.6%	1.18 [0.66, 2.12]	2013
Fei,Y.H.2014	24	40	15	40	2.4%	1.60 [1.00, 2.57]	2014
Zhang,Y.N.2015	23	46	11	38	1.6%	1.73 [0.97, 3.07]	2015
Huang,P.2016	29	34	22	33	6.9%	1.28 [0.97, 1.69]	2016
Ma,M.2016	16	22	12	22	2.5%	1.33 [0.84, 2.11]	2016
Xu,W.2019	40	66	27	66	4.4%	1.48 [1.04, 2.10]	2019
Hou,Y.2020	43	45	36	45	21.2%	1.19 [1.02, 1.40]	2020
**Subtotal (95% CI)**		**659**		**643**	**61.5%**	**1.27 [1.15, 1.39]**	
Total events	356		269				
Heterogeneity: Tau² = 0.00; Chi² = 6.08, df = 14 (P = 0.96); I² = 0%							
Test for overall effect: Z = 4.93 (P < 0.00001)							
**Objective Response Rate (Injection Administration Group)**							
Gong,L.Y.2006	16	26	10	30	1.5%	1.85 [1.02, 3.33]	2006
He,Z.Q. 2008	38	65	31	58	5.4%	1.09 [0.80, 1.50]	2008
Liu,X.Q.2009	12	30	10	30	1.2%	1.20 [0.61, 2.34]	2009
Liu,L.H.2009	16	30	16	30	2.4%	1.00 [0.62, 1.61]	2009
Fan,C.M.2011	11	23	10	28	1.3%	1.34 [0.70, 2.58]	2011
Wang,J.R.2012	12	26	10	24	1.4%	1.11 [0.59, 2.08]	2012
Ren,Y,Z.2012	15	33	14	32	1.8%	1.04 [0.60, 1.79]	2012
Li,H.Y.2013	20	35	13	35	2.0%	1.54 [0.92, 2.58]	2013
Yin,L.L.2013	14	26	14	27	2.1%	1.04 [0.62, 1.73]	2013
Wen,J.2014	9	15	8	15	1.4%	1.13 [0.60, 2.11]	2014
Zhang,L.2014	17	32	15	32	2.2%	1.13 [0.69, 1.85]	2014
Zhang,M.J.2014	21	48	20	48	2.5%	1.05 [0.66, 1.67]	2014
Xiang,S.L.2014	17	33	15	33	2.2%	1.13 [0.69, 1.87]	2014
Duan,F.2016	26	46	24	46	3.8%	1.08 [0.74, 1.58]	2016
Hu,Q.2017	13	18	7	18	1.3%	1.86 [0.97, 3.54]	2017
Zhu,Y.F.2019	16	30	12	30	1.8%	1.33 [0.77, 2.31]	2019
Yuan,d.d.2019	37	60	26	60	4.4%	1.42 [1.00, 2.02]	2019
**Subtotal (95% CI)**		**576**		**576**	**38.5%**	**1.20 [1.07, 1.35]**	
Total events	310		255				
Heterogeneity: Tau² = 0.00; Chi² = 8.14, df = 16 (P = 0.94); I² = 0%							
Test for overall effect: Z = 3.08 (P = 0.002)							
**Total (95% CI)**		**1235**		**1219**	**100.0%**	**1.24 [1.15, 1.34]**	
Total events	666		524				
Heterogeneity: Tau² = 0.00; Chi² = 14.48, df = 31 (P = 0.99); I² = 0%							
Test for overall effect: Z = 5.77 (P < 0.00001)							
Test for subgroup differences: Chi² = 0.42, df = 1 (P = 0.52), I² = 0%							

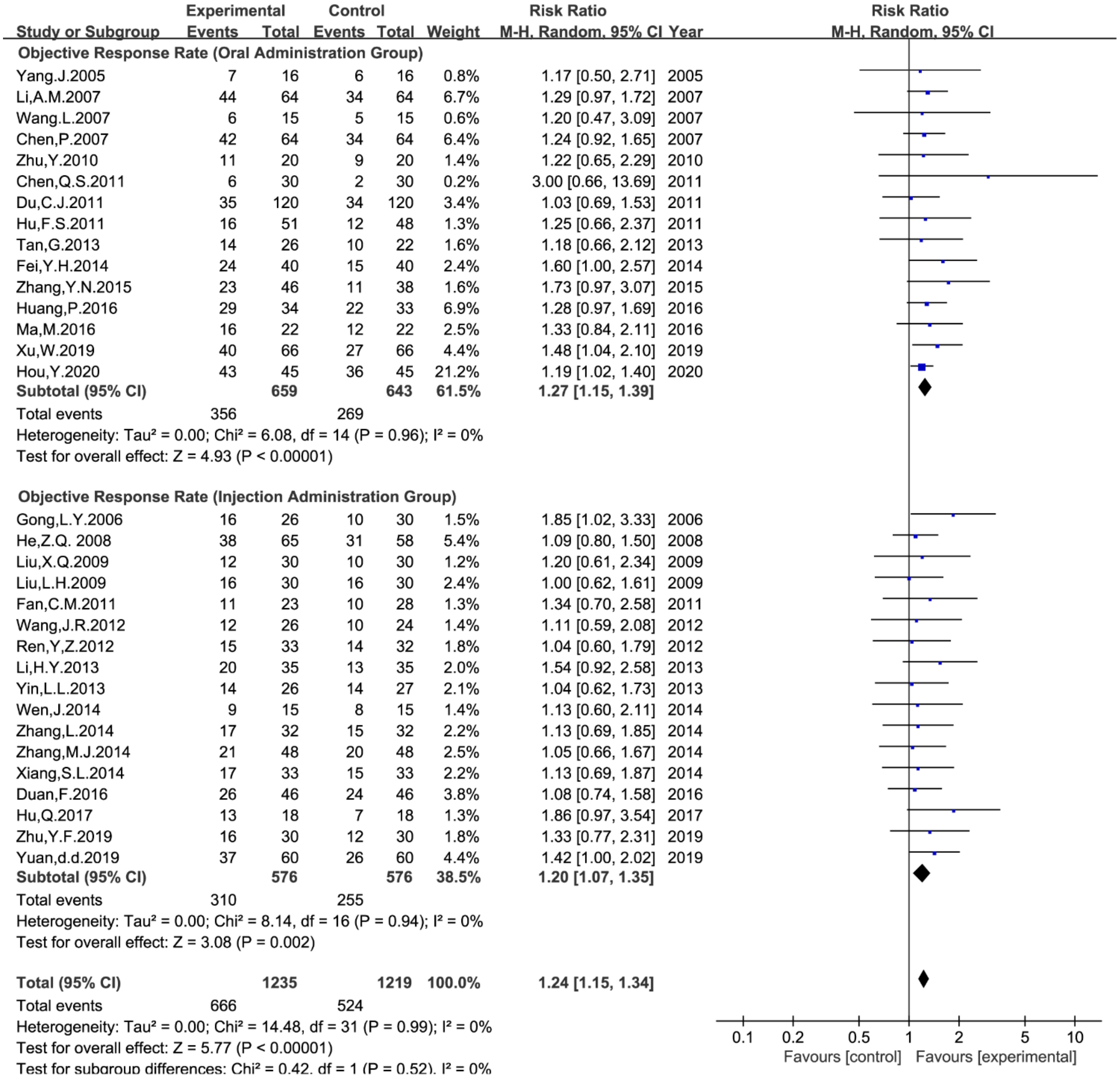

Figure S11. Subgroup analysis of ORR via drug delivery

Study or Subgroup	Experimental Events	Experimental Total	Control Events	Control Total	Weight	Risk Ratio M-H, Random, 95% CI	Year
**Disease Control Rate (Oral Administration Group)**							
Yang.J.2005	13	16	11	16	0.9%	1.18 [0.79, 1.77]	2005
Wang.L.2007	12	15	11	15	0.9%	1.09 [0.73, 1.62]	2007
Chen,P.2007	56	64	52	64	5.4%	1.08 [0.93, 1.25]	2007
Li,A.M.2007	58	64	52	64	5.9%	1.12 [0.97, 1.29]	2007
Zhu,Y.2010	18	20	13	20	1.1%	1.38 [0.97, 1.97]	2010
Hu,F.S.2011	31	51	20	48	0.9%	1.46 [0.98, 2.18]	2011
Du,C.J.2011	97	120	95	120	7.1%	1.02 [0.90, 1.16]	2011
Chen,Q.S.2011	26	30	13	30	0.8%	2.00 [1.30, 3.08]	2011
Tan,G.2013	22	26	17	22	1.8%	1.10 [0.83, 1.45]	2013
Fei,Y.H.2014	33	40	30	40	2.6%	1.10 [0.87, 1.38]	2014
Zhang,Y.N.2015	36	46	18	38	1.1%	1.65 [1.14, 2.39]	2015
Huang,P.2016	31	34	27	33	3.5%	1.11 [0.92, 1.35]	2016
Ma,M.2016	20	22	16	22	1.7%	1.25 [0.94, 1.67]	2016
Xu,W.2019	58	66	53	66	5.4%	1.09 [0.94, 1.27]	2019
Hou,Y.2020	45	45	40	45	8.6%	1.12 [1.01, 1.25]	2020
**Subtotal (95% CI)**		**659**		**643**	**47.7%**	**1.14 [1.07, 1.22]**	
Total events	556		468				
Heterogeneity: Tau² = 0.00; Chi² = 19.09, df = 14 (P = 0.16); I² = 27%							
Test for overall effect: Z = 4.03 (P < 0.0001)							
**Disease Control Rate (Injection Administration Group)**							
Gong,L.Y.2006	23	26	24	30	2.6%	1.11 [0.88, 1.39]	2006
He,Z.Q. 2008	61	65	50	58	7.6%	1.09 [0.97, 1.23]	2008
Liu,L.H.2009	27	30	28	30	5.2%	0.96 [0.83, 1.12]	2009
Liu,X.Q.2009	27	30	26	30	3.8%	1.04 [0.86, 1.25]	2009
Fan,C.M.2011	20	23	24	28	2.8%	1.01 [0.82, 1.26]	2011
Wang,J.R.2012	21	26	18	24	1.6%	1.08 [0.80, 1.45]	2012
Ren,Y,Z.2012	31	33	29	32	6.0%	1.04 [0.90, 1.19]	2012
Li,H.Y.2013	32	35	23	35	2.0%	1.39 [1.07, 1.80]	2013
Yin,L.L.2013	23	26	22	27	2.6%	1.09 [0.87, 1.36]	2013
Zhang,L.2014	26	32	25	32	2.2%	1.04 [0.81, 1.33]	2014
Wen,J.2014	13	15	13	15	1.8%	1.00 [0.76, 1.32]	2014
Xiang,S.L.2014	27	33	28	33	2.9%	0.96 [0.78, 1.20]	2014
Duan,F.2016	39	46	36	46	3.4%	1.08 [0.89, 1.32]	2016
Hu,Q.2017	16	18	10	18	0.7%	1.60 [1.03, 2.50]	2017
Zhu,Y.F.2019	25	30	21	30	1.7%	1.19 [0.90, 1.58]	2019
Yuan,d.d.2019	54	60	48	60	5.3%	1.13 [0.97, 1.31]	2019
**Subtotal (95% CI)**		**528**		**528**	**52.3%**	**1.07 [1.02, 1.12]**	
Total events	465		425				
Heterogeneity: Tau² = 0.00; Chi² = 12.39, df = 15 (P = 0.65); I² = 0%							
Test for overall effect: Z = 2.73 (P = 0.006)							
**Total (95% CI)**		**1187**		**1171**	**100.0%**	**1.10 [1.06, 1.14]**	
Total events	1021		893				
Heterogeneity: Tau² = 0.00; Chi² = 33.71, df = 30 (P = 0.29); I² = 11%							
Test for overall effect: Z = 4.85 (P < 0.00001)							
Test for subgroup differences: Chi² = 2.35, df = 1 (P = 0.13), I² = 57.4%							

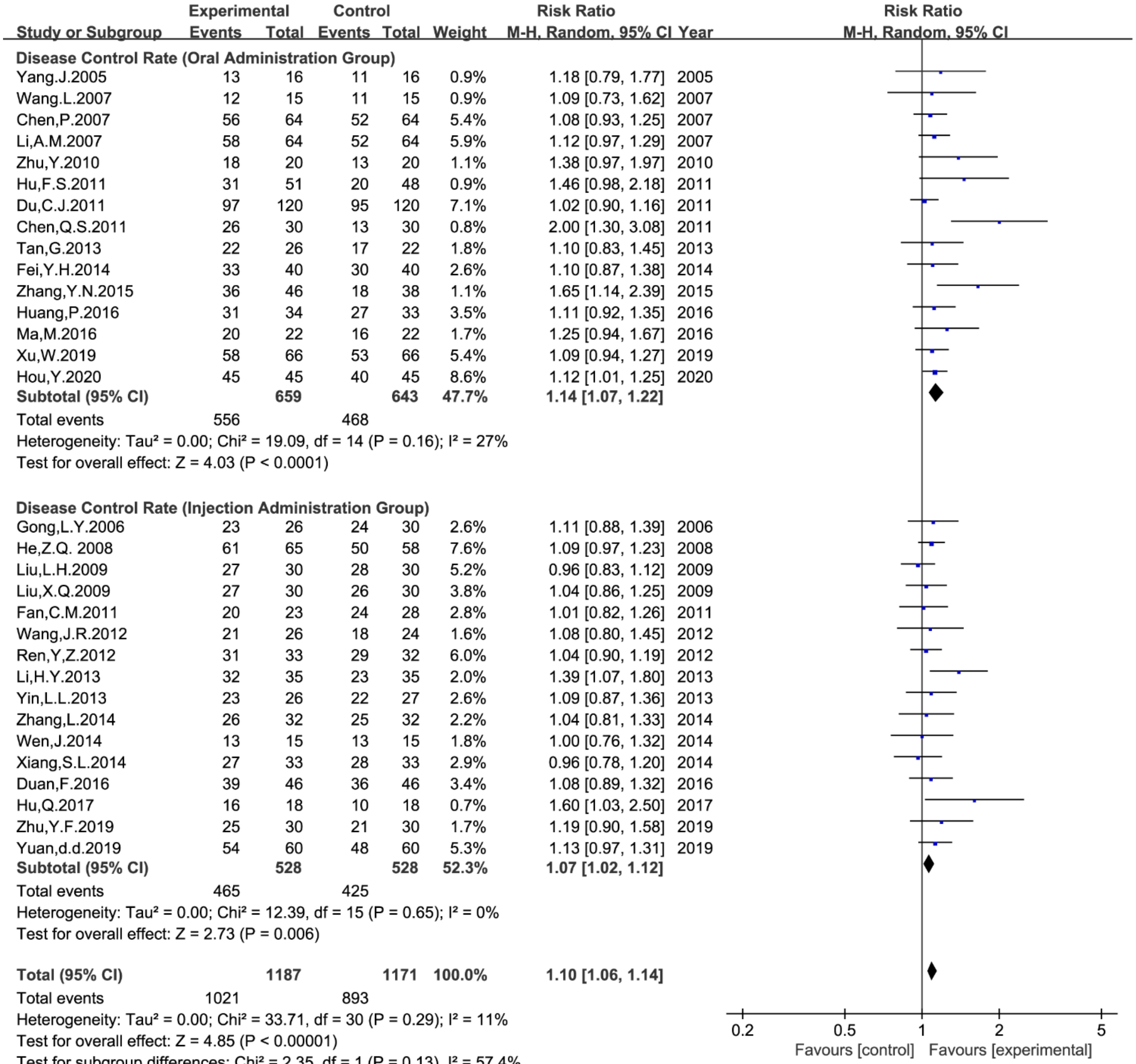

Figure S12. Subgroup analysis of DCR via drug delivery

Study or Subgroup	Experimental Events	Experimental Total	Control Events	Control Total	Weight	Risk Ratio M-H, Random, 95% CI	Year
**Objective response rate (OXA-based chemotherapy regimen group)**							
Yang.J.2005	7	16	6	16	0.8%	1.17 [0.50, 2.71]	2005
Wang.L.2007	6	15	5	15	0.6%	1.20 [0.47, 3.09]	2007
Chen,P.2007	42	64	34	64	6.4%	1.24 [0.92, 1.65]	2007
He,Z.Q. 2008	38	65	31	58	5.4%	1.09 [0.80, 1.50]	2008
Zhu,Y.2010	11	20	9	20	1.4%	1.22 [0.65, 2.29]	2010
Chen,Q.S.2011	6	30	2	30	0.2%	3.00 [0.66, 13.69]	2011
Du,C.J.2011	35	120	34	120	3.4%	1.03 [0.69, 1.53]	2011
Fan,C.M.2011	11	23	10	28	1.3%	1.34 [0.70, 2.58]	2011
Hu,F.S.2011	16	51	12	48	1.3%	1.25 [0.66, 2.37]	2011
Ren,Y,Z.2012	15	33	14	32	1.8%	1.04 [0.60, 1.79]	2012
Li,H.Y.2013	20	35	13	35	2.0%	1.54 [0.92, 2.58]	2013
Tan,G.2013	14	26	10	22	1.6%	1.18 [0.66, 2.12]	2013
Zhang,M.J.2014	21	48	20	48	2.5%	1.05 [0.66, 1.67]	2014
Zhang,L.2014	17	32	15	32	2.2%	1.13 [0.69, 1.85]	2014
Wen,J.2014	9	15	8	15	1.4%	1.13 [0.60, 2.11]	2014
Zhang,Y.N.2015	23	46	11	38	1.6%	1.73 [0.97, 3.07]	2015
Huang,P.2016	29	34	22	33	6.9%	1.28 [0.97, 1.69]	2016
Ma,M.2016	16	22	12	22	2.5%	1.33 [0.84, 2.11]	2016
Hu,Q.2017	13	18	7	18	1.3%	1.86 [0.97, 3.54]	2017
Xu,W.2019	40	66	27	66	4.4%	1.48 [1.04, 2.10]	2019
Zhu,Y.F.2019	16	30	12	30	1.8%	1.33 [0.77, 2.31]	2019
Hou,Y.2020	43	45	36	45	21.2%	1.19 [1.02, 1.40]	2020
**Subtotal (95% CI)**		**854**		**835**	**72.0%**	**1.24 [1.13, 1.35]**	
Total events	448		350				
Heterogeneity: Tau² = 0.00; Chi² = 8.91, df = 21 (P = 0.99); I² = 0%							
Test for overall effect: Z = 4.81 (P < 0.00001)							
**Objective response rate (DDP-based chemotherapy regimen group)**							
Gong,L.Y.2006	16	26	10	30	1.5%	1.85 [1.02, 3.33]	2006
Li,A.M.2007	44	64	34	64	6.7%	1.29 [0.97, 1.72]	2007
Liu,X.Q.2009	12	30	10	30	1.2%	1.20 [0.61, 2.34]	2009
Liu,L.H.2009	16	30	16	30	2.4%	1.00 [0.62, 1.61]	2009
Wang,J.R.2012	12	26	10	24	1.4%	1.11 [0.59, 2.08]	2012
Yin,L.L.2013	14	26	14	27	2.1%	1.04 [0.62, 1.73]	2013
Xiang,S.L.2014	17	33	15	33	2.2%	1.13 [0.69, 1.87]	2014
Fei,Y.H.2014	24	40	15	40	2.4%	1.60 [1.00, 2.57]	2014
Duan,F.2016	26	46	24	46	3.8%	1.08 [0.74, 1.58]	2016
Yuan,d.d.2019	37	60	26	60	4.4%	1.42 [1.00, 2.02]	2019
**Subtotal (95% CI)**		**381**		**384**	**28.0%**	**1.25 [1.09, 1.44]**	
Total events	218		174				
Heterogeneity: Tau² = 0.00; Chi² = 5.53, df = 9 (P = 0.79); I² = 0%							
Test for overall effect: Z = 3.21 (P = 0.001)							
**Total (95% CI)**		**1235**		**1219**	**100.0%**	**1.24 [1.15, 1.34]**	
Total events	666		524				
Heterogeneity: Tau² = 0.00; Chi² = 14.48, df = 31 (P = 0.99); I² = 0%							
Test for overall effect: Z = 5.77 (P < 0.00001)							
Test for subgroup differences: Chi² = 0.03, df = 1 (P = 0.86), I² = 0%							

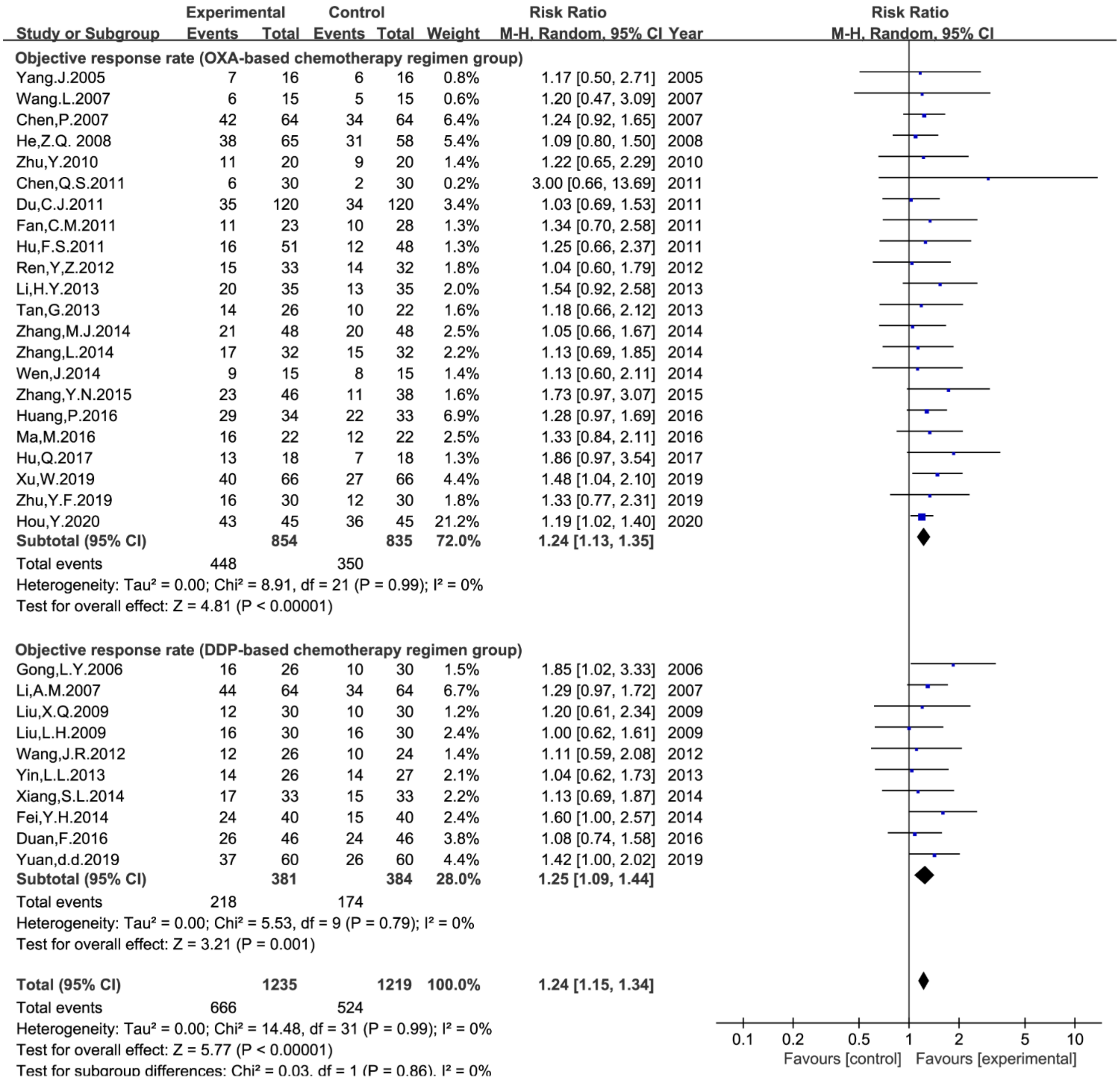

Figure S13. Subgroup analysis of ORR via chemotherapy regimen

Study or Subgroup	Experimental Events	Experimental Total	Control Events	Control Total	Weight	Risk Ratio M-H, Random, 95% CI	Year
**Disease control rate (OXA-based chemotherapy regimen group)**							
Yang.J.2005	13	16	11	16	0.9%	1.18 [0.79, 1.77]	2005
Wang.L.2007	12	15	11	15	0.9%	1.09 [0.73, 1.62]	2007
Chen,P.2007	56	64	52	64	5.4%	1.08 [0.93, 1.25]	2007
He,Z.Q. 2008	61	65	50	58	7.6%	1.09 [0.97, 1.23]	2008
Zhu,Y.2010	18	20	13	20	1.1%	1.38 [0.97, 1.97]	2010
Fan,C.M.2011	20	23	24	28	2.8%	1.01 [0.82, 1.26]	2011
Hu,F.S.2011	31	51	20	48	0.9%	1.46 [0.98, 2.18]	2011
Chen,Q.S.2011	26	30	13	30	0.8%	2.00 [1.30, 3.08]	2011
Du,C.J.2011	97	120	95	120	7.1%	1.02 [0.90, 1.16]	2011
Ren,Y,Z.2012	31	33	29	32	6.0%	1.04 [0.90, 1.19]	2012
Tan,G.2013	22	26	17	22	1.8%	1.10 [0.83, 1.45]	2013
Li,H.Y.2013	32	35	23	35	2.0%	1.39 [1.07, 1.80]	2013
Wen,J.2014	13	15	13	15	1.8%	1.00 [0.76, 1.32]	2014
Zhang,L.2014	26	32	25	32	2.2%	1.04 [0.81, 1.33]	2014
Zhang,Y.N.2015	36	46	18	38	1.1%	1.65 [1.14, 2.39]	2015
Huang,P.2016	31	34	27	33	3.5%	1.11 [0.92, 1.35]	2016
Ma,M.2016	20	22	16	22	1.7%	1.25 [0.94, 1.67]	2016
Hu,Q.2017	16	18	10	18	0.7%	1.60 [1.03, 2.50]	2017
Zhu,Y.F.2019	25	30	21	30	1.7%	1.19 [0.90, 1.58]	2019
Xu,W.2019	58	66	53	66	5.4%	1.09 [0.94, 1.27]	2019
Hou,Y.2020	45	45	40	45	8.6%	1.12 [1.01, 1.25]	2020
**Subtotal (95% CI)**		**806**		**787**	**64.1%**	**1.13 [1.07, 1.20]**	
Total events	689		581				
Heterogeneity: Tau² = 0.00; Chi² = 29.10, df = 20 (P = 0.09); I² = 31%							
Test for overall effect: Z = 4.25 (P < 0.0001)							
**Disease control rate (DDP-based chemotherapy regimen group)**							
Gong,L.Y.2006	23	26	24	30	2.6%	1.11 [0.88, 1.39]	2006
Li,A.M.2007	58	64	52	64	5.9%	1.12 [0.97, 1.29]	2007
Liu,L.H.2009	27	30	28	30	5.2%	0.96 [0.83, 1.12]	2009
Liu,X.Q.2009	27	30	26	30	3.8%	1.04 [0.86, 1.25]	2009
Wang,J.R.2012	21	26	18	24	1.6%	1.08 [0.80, 1.45]	2012
Yin,L.L.2013	23	26	22	27	2.6%	1.09 [0.87, 1.36]	2013
Xiang,S.L.2014	27	33	28	33	2.9%	0.96 [0.78, 1.20]	2014
Fei,Y.H.2014	33	40	30	40	2.6%	1.10 [0.87, 1.38]	2014
Duan,F.2016	39	46	36	46	3.4%	1.08 [0.89, 1.32]	2016
Yuan,d.d.2019	54	60	48	60	5.3%	1.13 [0.97, 1.31]	2019
**Subtotal (95% CI)**		**381**		**384**	**35.9%**	**1.06 [1.00, 1.13]**	
Total events	332		312				
Heterogeneity: Tau² = 0.00; Chi² = 3.73, df = 9 (P = 0.93); I² = 0%							
Test for overall effect: Z = 2.06 (P = 0.04)							
**Total (95% CI)**		**1187**		**1171**	**100.0%**	**1.10 [1.06, 1.14]**	
Total events	1021		893				
Heterogeneity: Tau² = 0.00; Chi² = 33.71, df = 30 (P = 0.29); I² = 11%							
Test for overall effect: Z = 4.85 (P < 0.00001)							
Test for subgroup differences: Chi² = 2.19, df = 1 (P = 0.14), I² = 54.2%							

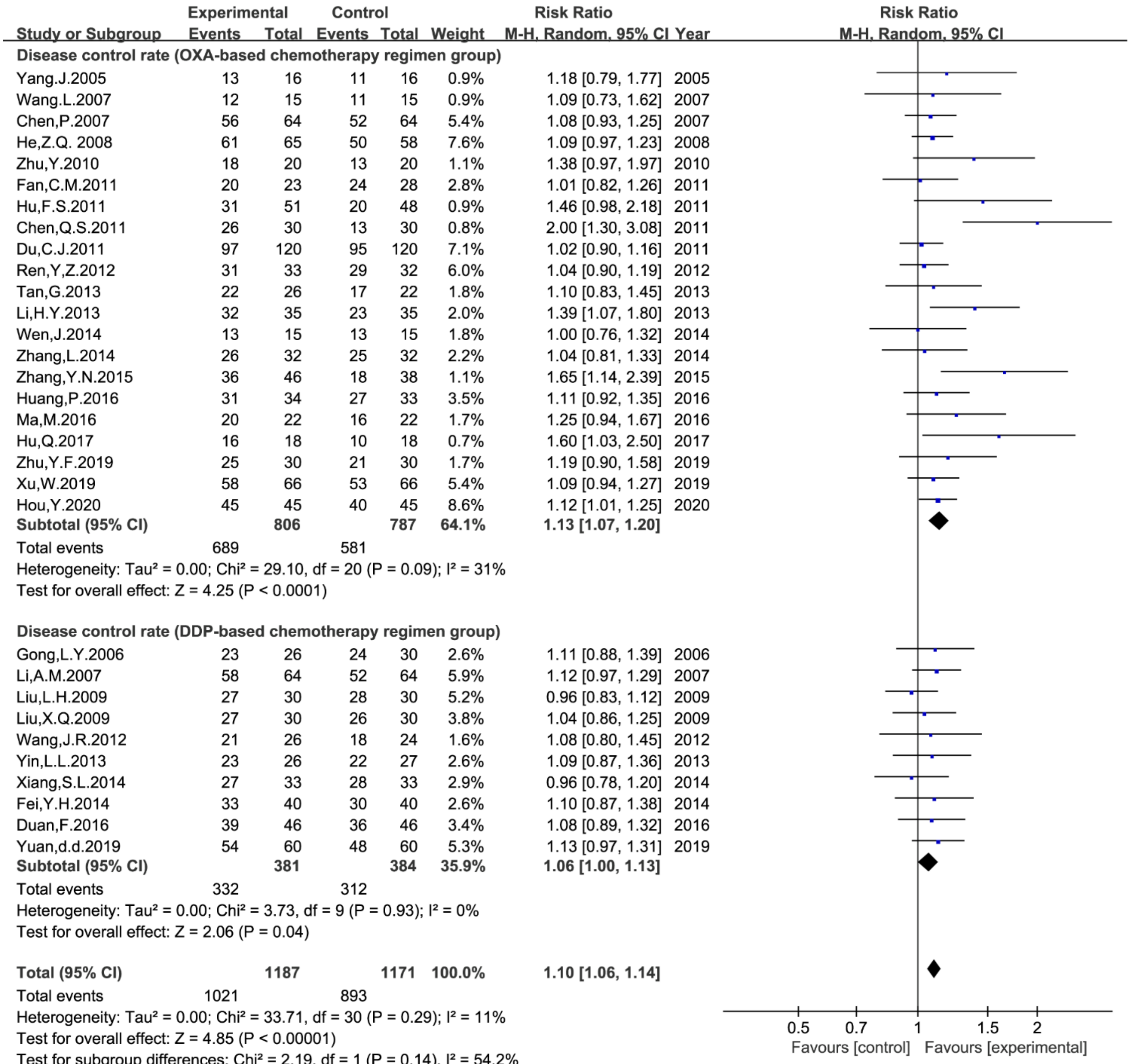

Figure S14. Subgroup analysis of DCR via chemotherapy regimen

Study or Subgroup	Experimental Events	Experimental Total	Control Events	Control Total	Weight	Risk Ratio M-H, Random, 95% CI	Year
**Objective Response Rate (≥8 weeks Group)**							
Yang.J.2005	7	16	6	16	0.8%	1.17 [0.50, 2.71]	2005
Gong,L.Y.2006	16	26	10	30	1.5%	1.85 [1.02, 3.33]	2006
Wang.L.2007	6	15	5	15	0.6%	1.20 [0.47, 3.09]	2007
Li,A.M.2007	44	64	34	64	6.7%	1.29 [0.97, 1.72]	2007
Chen,P.2007	42	64	34	64	6.4%	1.24 [0.92, 1.65]	2007
He,Z.Q. 2008	38	65	31	58	5.4%	1.09 [0.80, 1.50]	2008
Liu,L.H.2009	16	30	16	30	2.4%	1.00 [0.62, 1.61]	2009
Zhu,Y.2010	11	20	9	20	1.4%	1.22 [0.65, 2.29]	2010
Hu,F.S.2011	16	51	12	48	1.3%	1.25 [0.66, 2.37]	2011
Fan,C.M.2011	11	23	10	28	1.3%	1.34 [0.70, 2.58]	2011
Wang,J.R.2012	12	26	10	24	1.4%	1.11 [0.59, 2.08]	2012
Yin,L.L.2013	14	26	14	27	2.1%	1.04 [0.62, 1.73]	2013
Tan,G.2013	14	26	10	22	1.6%	1.18 [0.66, 2.12]	2013
Li,H.Y.2013	20	35	13	35	2.0%	1.54 [0.92, 2.58]	2013
Xiang,S.L.2014	17	33	15	33	2.2%	1.13 [0.69, 1.87]	2014
Wen,J.2014	9	15	8	15	1.4%	1.13 [0.60, 2.11]	2014
Zhang,Y.N.2015	23	46	11	38	1.6%	1.73 [0.97, 3.07]	2015
Duan,F.2016	26	46	24	46	3.8%	1.08 [0.74, 1.58]	2016
Hu,Q.2017	13	18	7	18	1.3%	1.86 [0.97, 3.54]	2017
Yuan,d.d.2019	37	60	26	60	4.4%	1.42 [1.00, 2.02]	2019
Xu,W.2019	40	66	27	66	4.4%	1.48 [1.04, 2.10]	2019
Zhu,Y.F.2019	16	30	12	30	1.8%	1.33 [0.77, 2.31]	2019
**Subtotal (95% CI)**		**801**		**787**	**55.5%**	**1.27 [1.15, 1.40]**	
Total events	448		344				
Heterogeneity: Tau² = 0.00; Chi² = 9.57, df = 21 (P = 0.98); I² = 0%							
Test for overall effect: Z = 4.74 (P < 0.00001)							
**Objective Response Rate (＜8weeks Group)**							
Liu,X.Q.2009	12	30	10	30	1.2%	1.20 [0.61, 2.34]	2009
Du,C.J.2011	35	120	34	120	3.4%	1.03 [0.69, 1.53]	2011
Chen,Q.S.2011	6	30	2	30	0.2%	3.00 [0.66, 13.69]	2011
Ren,Y,Z.2012	15	33	14	32	1.8%	1.04 [0.60, 1.79]	2012
Zhang,M.J.2014	21	48	20	48	2.5%	1.05 [0.66, 1.67]	2014
Zhang,L.2014	17	32	15	32	2.2%	1.13 [0.69, 1.85]	2014
Fei,Y.H.2014	24	40	15	40	2.4%	1.60 [1.00, 2.57]	2014
Ma,M.2016	16	22	12	22	2.5%	1.33 [0.84, 2.11]	2016
Huang,P.2016	29	34	22	33	6.9%	1.28 [0.97, 1.69]	2016
Hou,Y.2020	43	45	36	45	21.2%	1.19 [1.02, 1.40]	2020
**Subtotal (95% CI)**		**434**		**432**	**44.5%**	**1.21 [1.08, 1.35]**	
Total events	218		180				
Heterogeneity: Tau² = 0.00; Chi² = 4.43, df = 9 (P = 0.88); I² = 0%							
Test for overall effect: Z = 3.36 (P = 0.0008)							
**Total (95% CI)**		**1235**		**1219**	**100.0%**	**1.24 [1.15, 1.34]**	
Total events	666		524				
Heterogeneity: Tau² = 0.00; Chi² = 14.48, df = 31 (P = 0.99); I² = 0%							
Test for overall effect: Z = 5.77 (P < 0.00001)							
Test for subgroup differences: Chi² = 0.43, df = 1 (P = 0.51), I² = 0%							

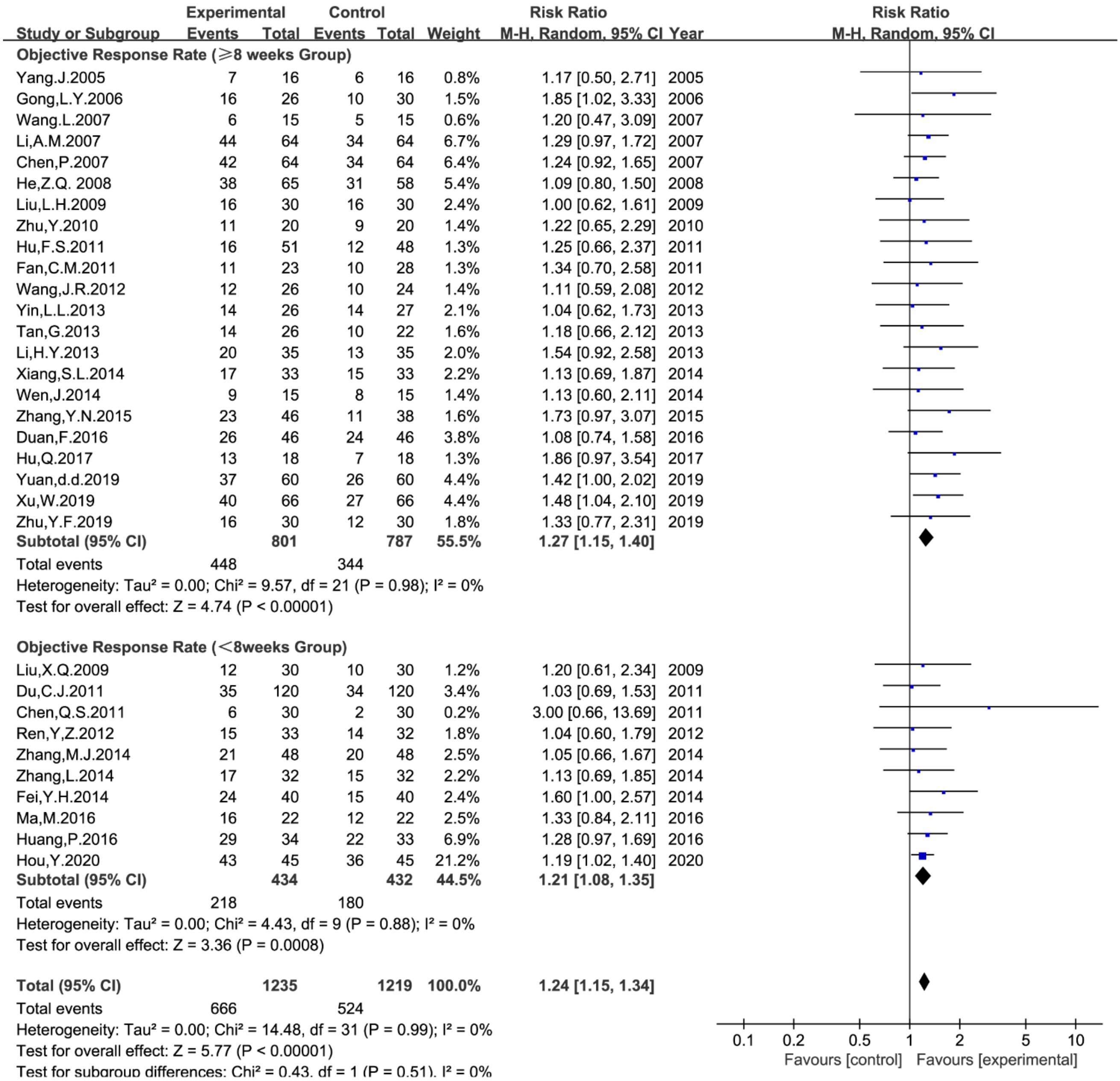

Figure S15. Subgroup analysis of ORR via treatment duration

Study or Subgroup	Experimental Events	Experimental Total	Control Events	Control Total	Weight	Risk Ratio M-H, Random, 95% CI	Year
**Disease Control Rate (≥8 weeks Group)**							
Yang.J.2005	13	16	11	16	0.9%	1.18 [0.79, 1.77]	2005
Gong,L.Y.2006	23	26	24	30	2.6%	1.11 [0.88, 1.39]	2006
Chen,P.2007	56	64	52	64	5.4%	1.08 [0.93, 1.25]	2007
Li,A.M.2007	58	64	52	64	5.9%	1.12 [0.97, 1.29]	2007
Wang.L.2007	12	15	11	15	0.9%	1.09 [0.73, 1.62]	2007
He,Z.Q. 2008	61	65	50	58	7.6%	1.09 [0.97, 1.23]	2008
Liu,L.H.2009	27	30	28	30	5.2%	0.96 [0.83, 1.12]	2009
Zhu,Y.2010	18	20	13	20	1.1%	1.38 [0.97, 1.97]	2010
Fan,C.M.2011	20	23	24	28	2.8%	1.01 [0.82, 1.26]	2011
Hu,F.S.2011	31	51	20	48	0.9%	1.46 [0.98, 2.18]	2011
Wang,J.R.2012	21	26	18	24	1.6%	1.08 [0.80, 1.45]	2012
Li,H.Y.2013	32	35	23	35	2.0%	1.39 [1.07, 1.80]	2013
Yin,L.L.2013	23	26	22	27	2.6%	1.09 [0.87, 1.36]	2013
Tan,G.2013	22	26	17	22	1.8%	1.10 [0.83, 1.45]	2013
Xiang,S.L.2014	27	33	28	33	2.9%	0.96 [0.78, 1.20]	2014
Wen,J.2014	13	15	13	15	1.8%	1.00 [0.76, 1.32]	2014
Zhang,Y.N.2015	36	46	18	38	1.1%	1.65 [1.14, 2.39]	2015
Duan,F.2016	39	46	36	46	3.4%	1.08 [0.89, 1.32]	2016
Hu,Q.2017	16	18	10	18	0.7%	1.60 [1.03, 2.50]	2017
Yuan,d.d.2019	54	60	48	60	5.3%	1.13 [0.97, 1.31]	2019
Xu,W.2019	58	66	53	66	5.4%	1.09 [0.94, 1.27]	2019
Zhu,Y.F.2019	25	30	21	30	1.7%	1.19 [0.90, 1.58]	2019
**Subtotal (95% CI)**		**801**		**787**	**63.7%**	**1.10 [1.05, 1.16]**	
Total events	685		592				
Heterogeneity: Tau² = 0.00; Chi² = 22.36, df = 21 (P = 0.38); I² = 6%							
Test for overall effect: Z = 4.14 (P < 0.0001)							
**Disease Control Rate (<8 weeks Group)**							
Liu,X.Q.2009	27	30	26	30	3.8%	1.04 [0.86, 1.25]	2009
Du,C.J.2011	97	120	95	120	7.1%	1.02 [0.90, 1.16]	2011
Chen,Q.S.2011	26	30	13	30	0.8%	2.00 [1.30, 3.08]	2011
Ren,Y,Z.2012	31	33	29	32	6.0%	1.04 [0.90, 1.19]	2012
Zhang,L.2014	26	32	25	32	2.2%	1.04 [0.81, 1.33]	2014
Fei,Y.H.2014	33	40	30	40	2.6%	1.10 [0.87, 1.38]	2014
Ma,M.2016	20	22	16	22	1.7%	1.25 [0.94, 1.67]	2016
Huang,P.2016	31	34	27	33	3.5%	1.11 [0.92, 1.35]	2016
Hou,Y.2020	45	45	40	45	8.6%	1.12 [1.01, 1.25]	2020
**Subtotal (95% CI)**		**386**		**384**	**36.3%**	**1.09 [1.02, 1.18]**	
Total events	336		301				
Heterogeneity: Tau² = 0.00; Chi² = 11.16, df = 8 (P = 0.19); I² = 28%							
Test for overall effect: Z = 2.45 (P = 0.01)							
**Total (95% CI)**		**1187**		**1171**	**100.0%**	**1.10 [1.06, 1.14]**	
Total events	1021		893				
Heterogeneity: Tau² = 0.00; Chi² = 33.71, df = 30 (P = 0.29); I² = 11%							
Test for overall effect: Z = 4.85 (P < 0.00001)							
Test for subgroup differences: Chi² = 0.04, df = 1 (P = 0.84), I² = 0%							

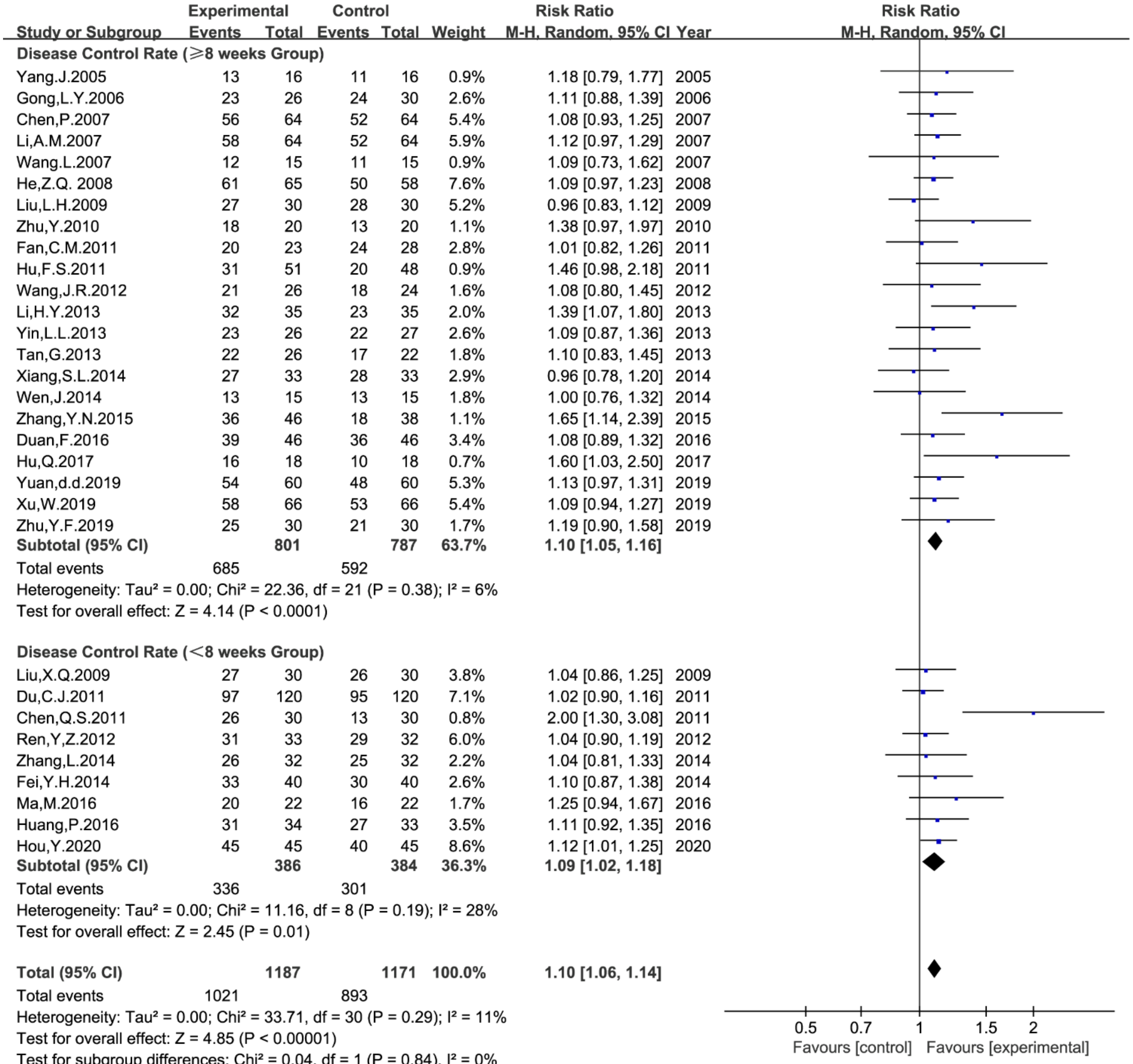

Figure S16. Subgroup analysis of DCR via treatment duration

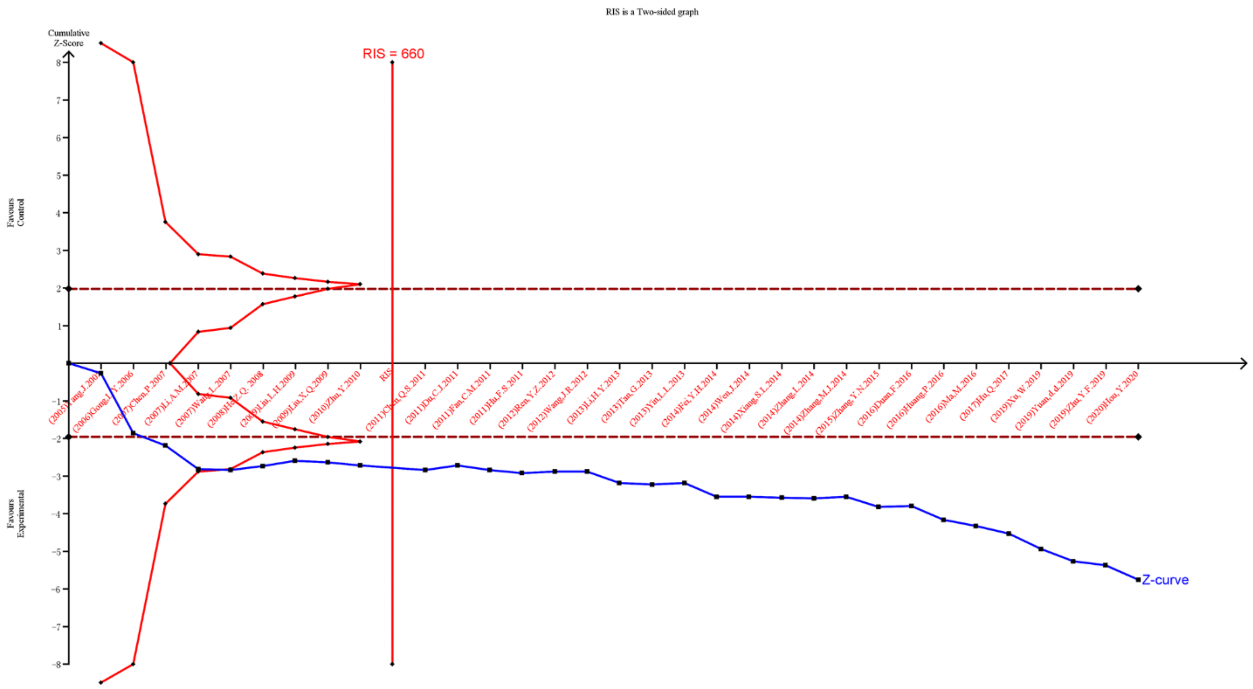

Figure S17. TSA of ORR

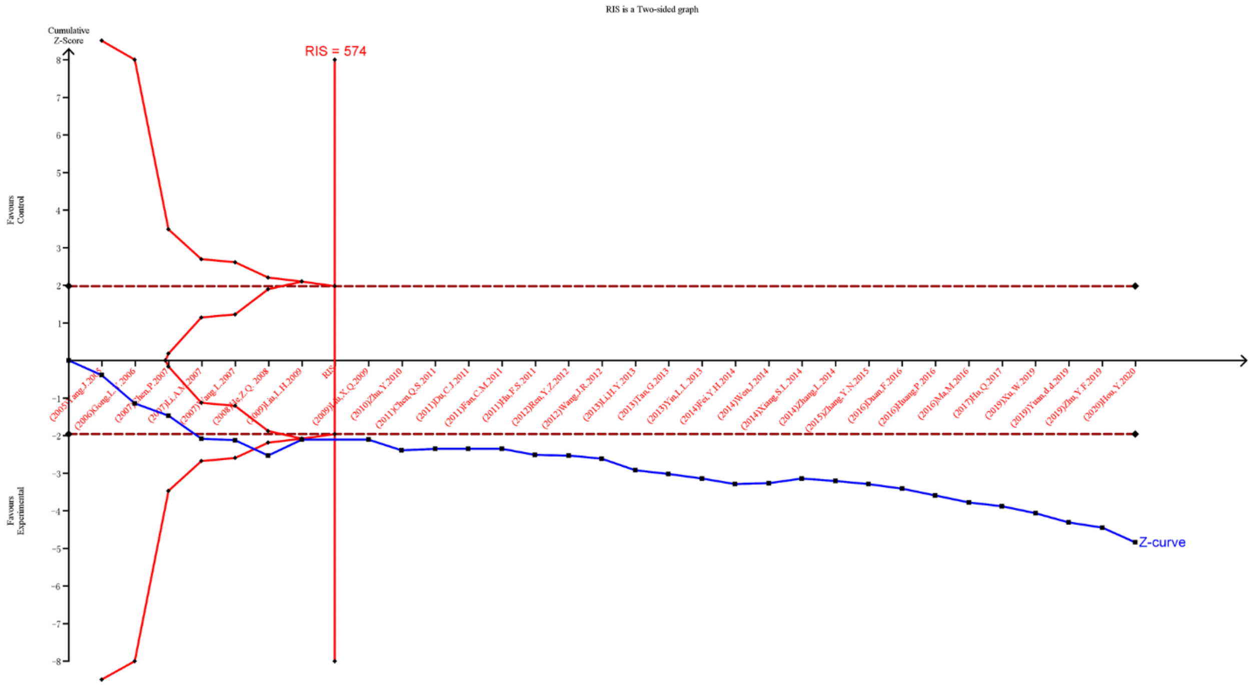

Figure S18. TSA of DCR

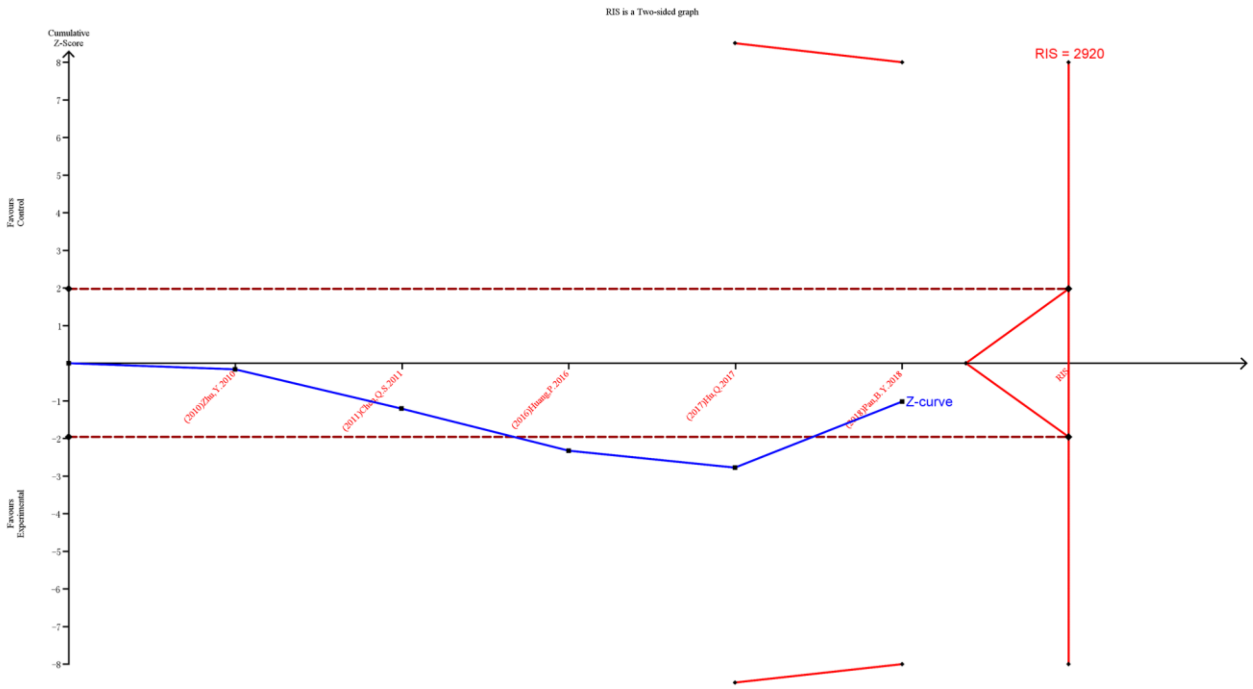

Figure S19. TSA of half-year survival rate

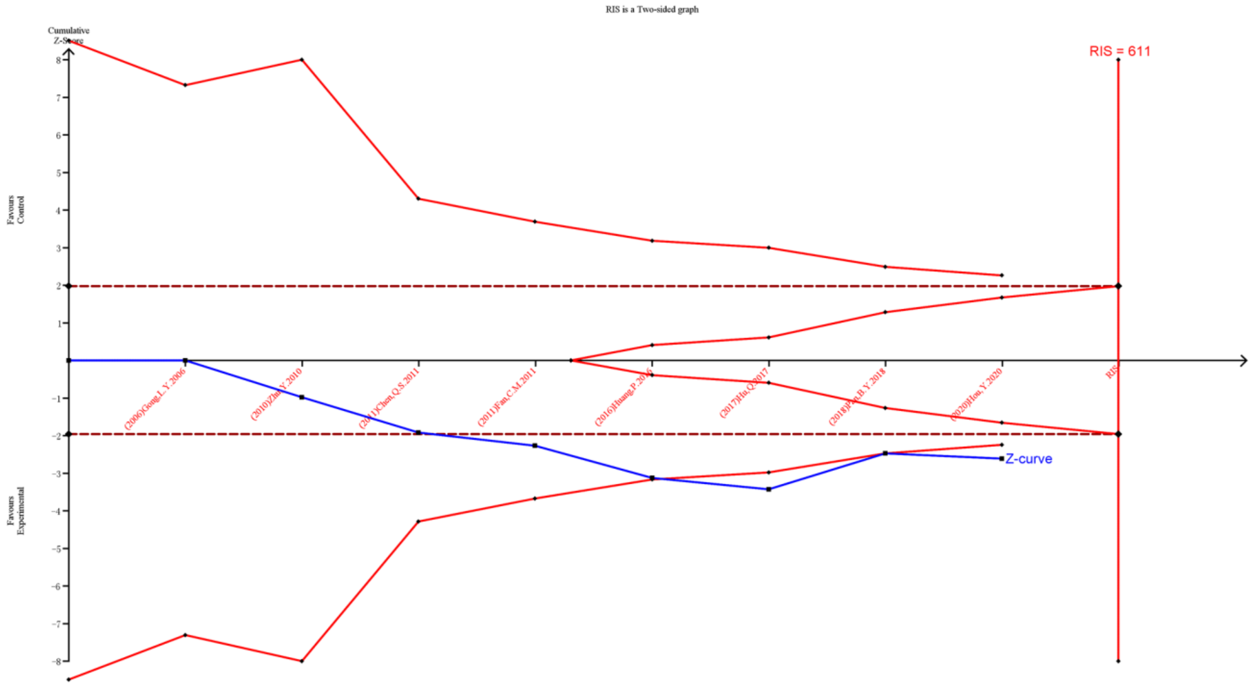

Figure S20. TSA of 1-year survival rate

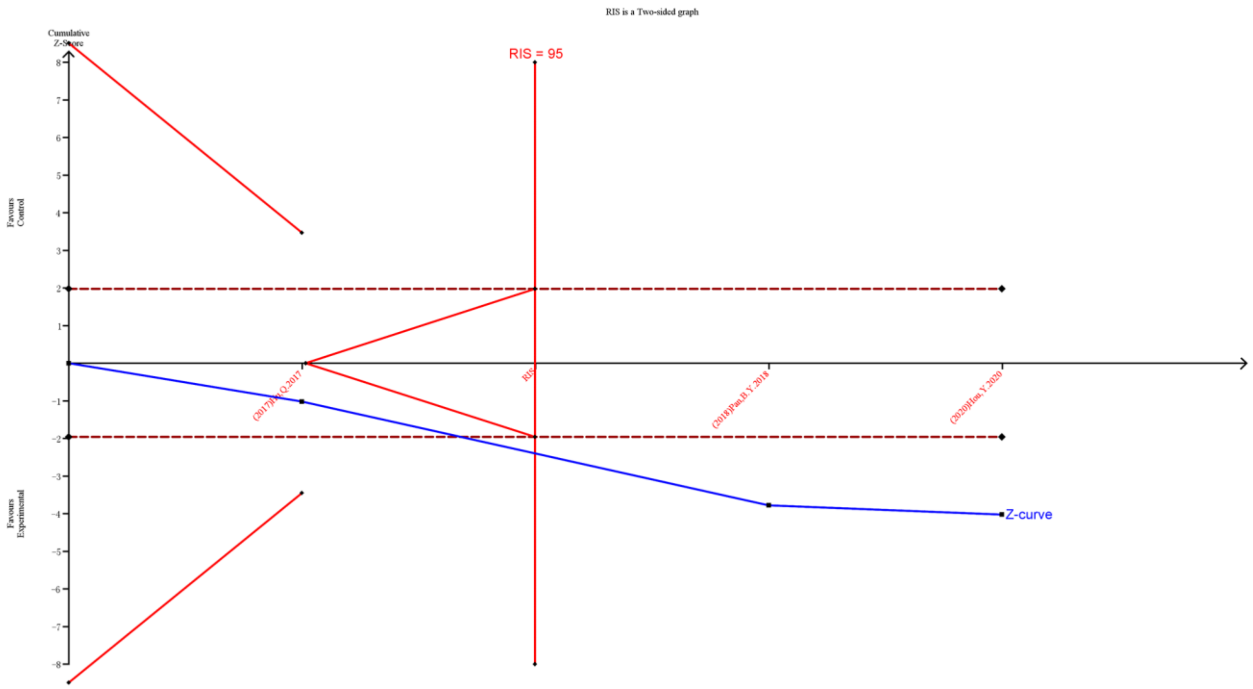

Figure S21. TSA of 2-year survival rate

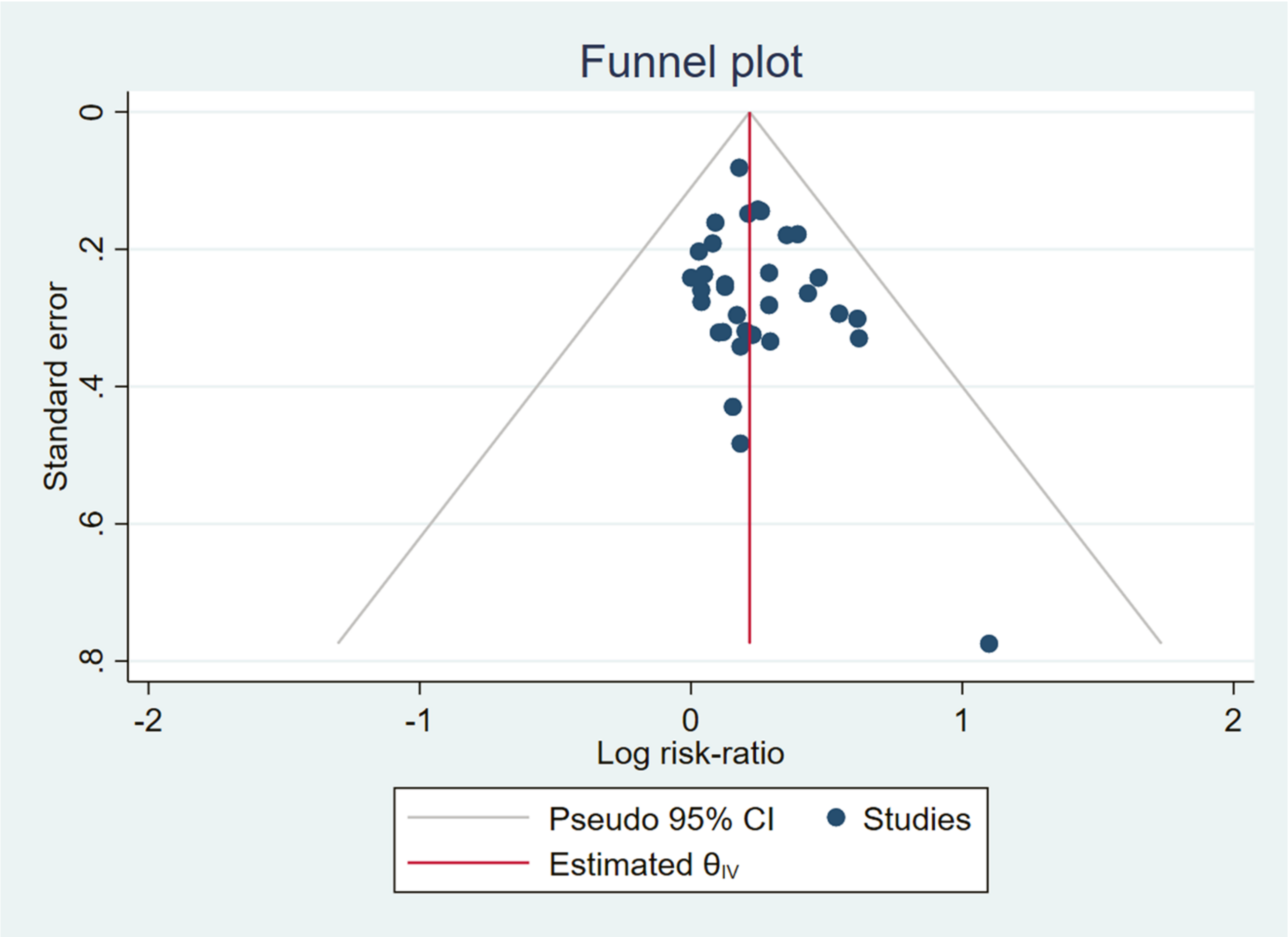

Figure S22. ORR funnel plot

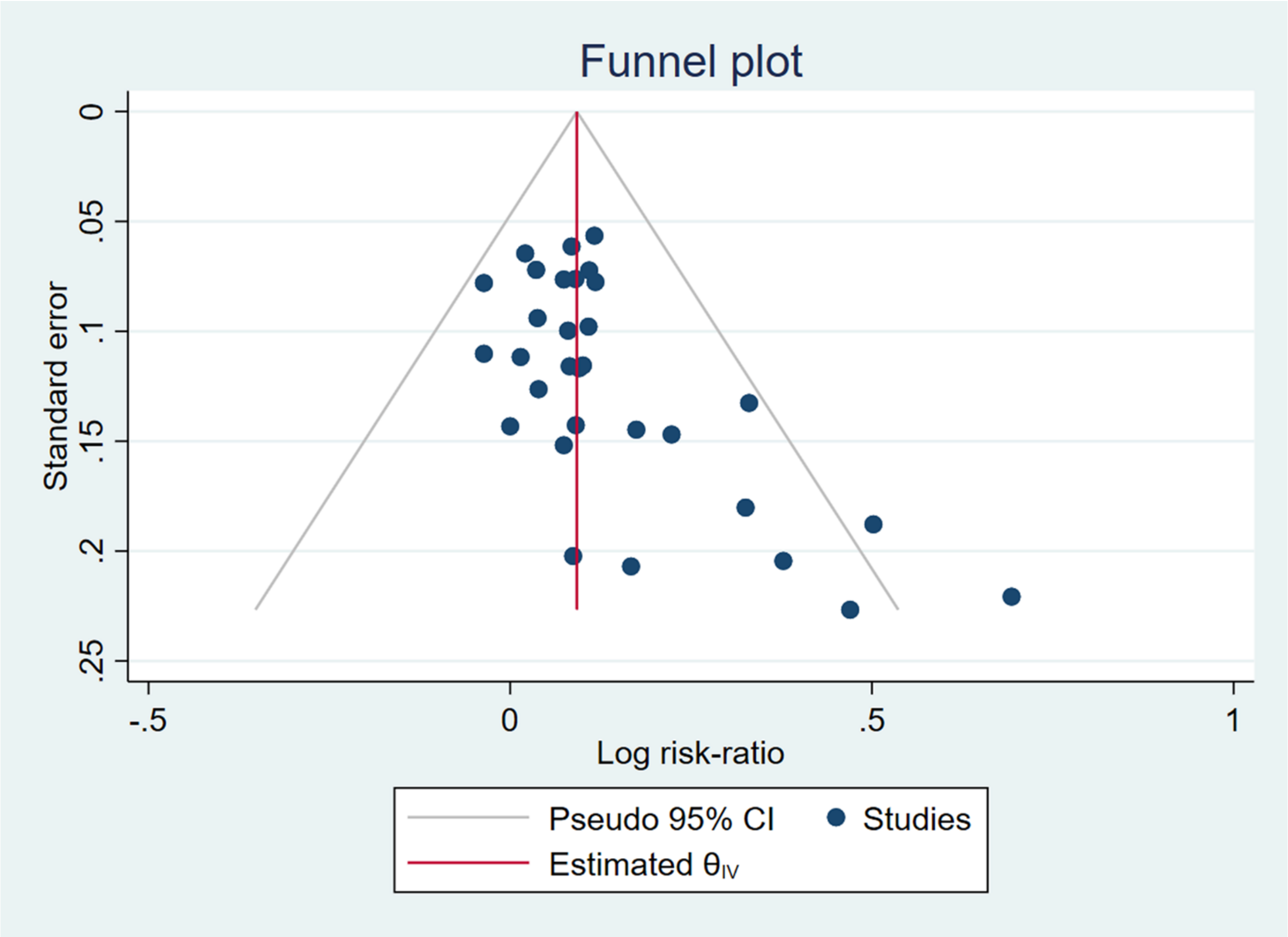

Figure S23. DCR funnel plot

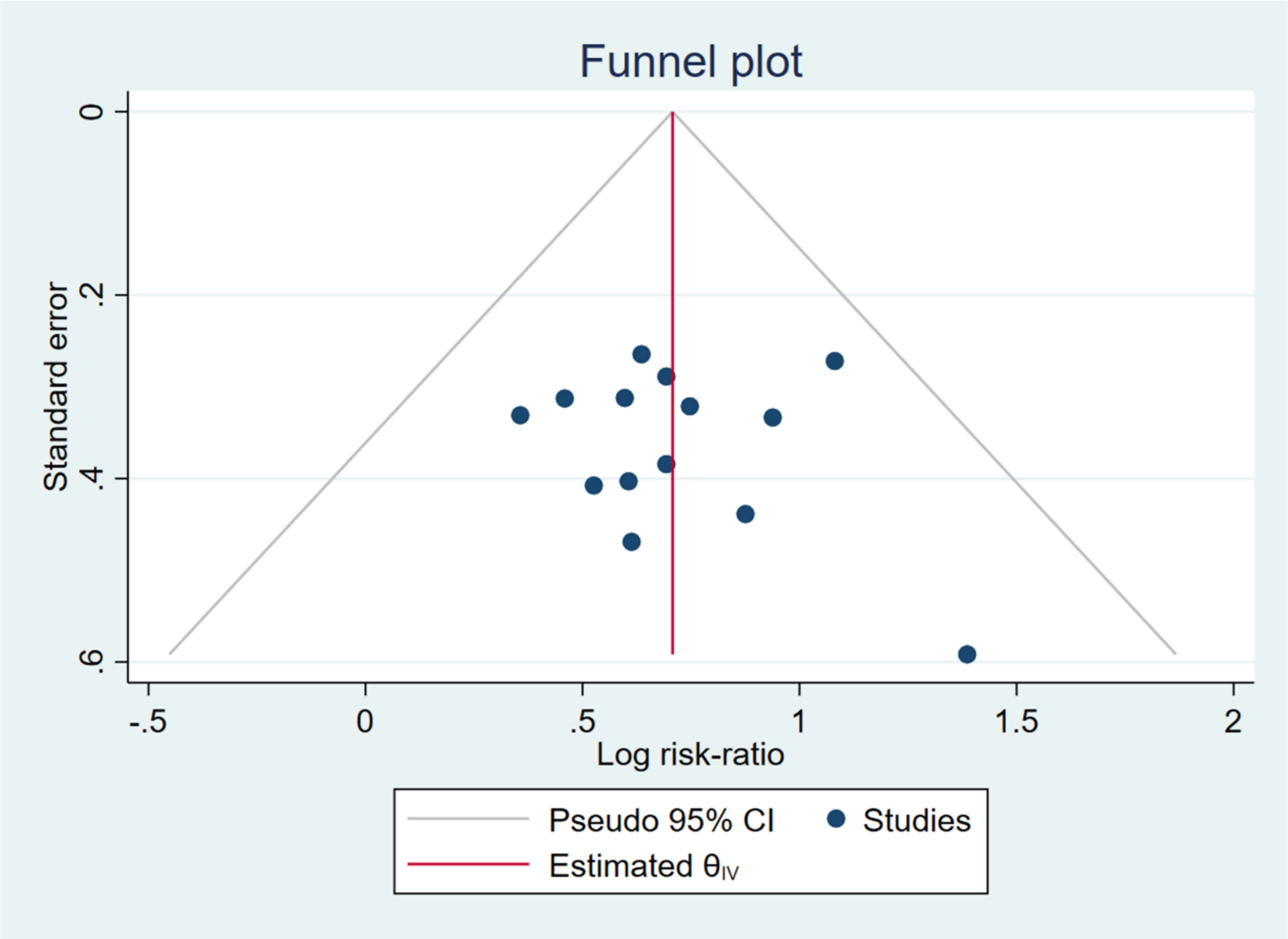

Figure S24. QOL funnel plot

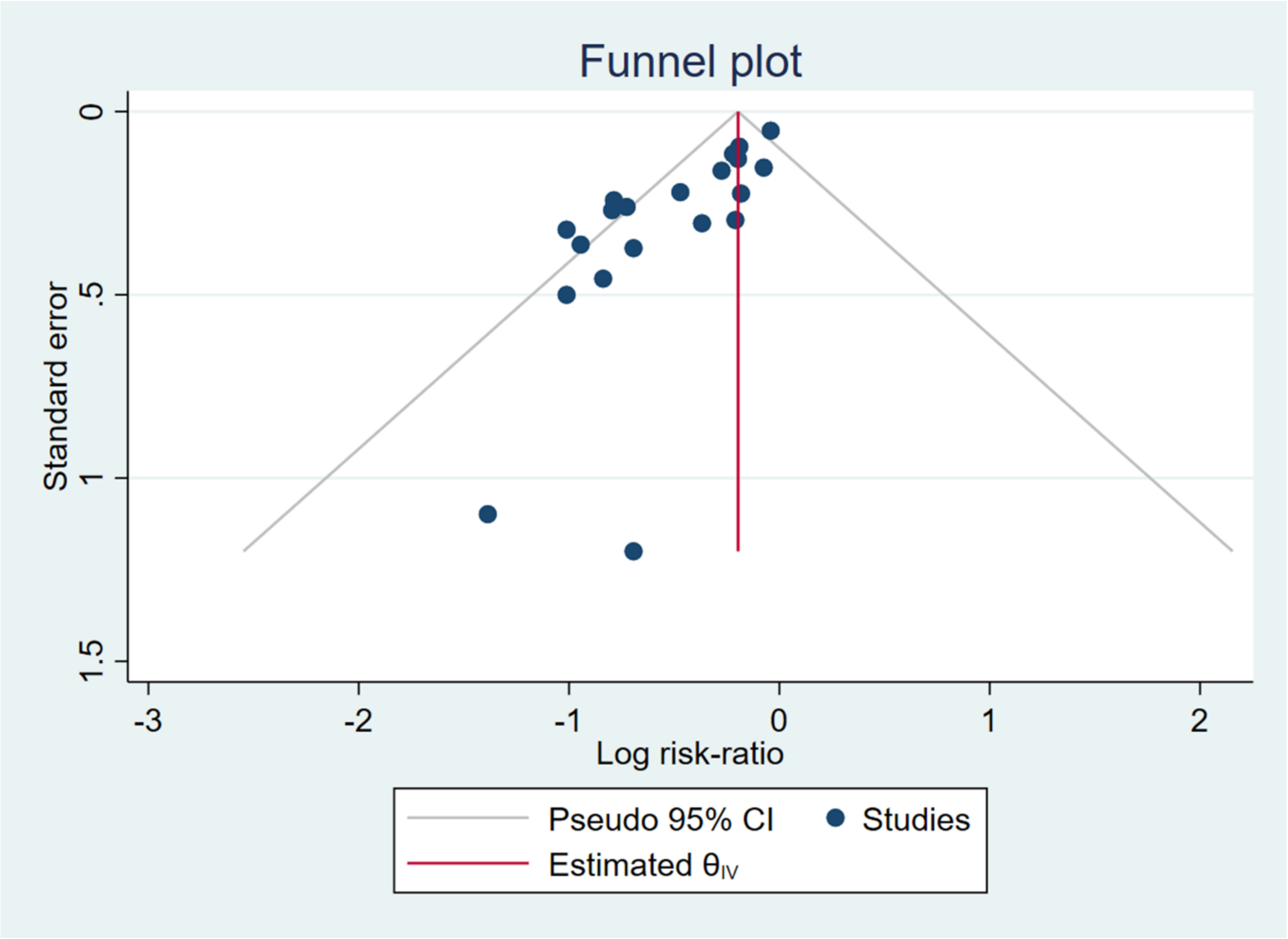

Figure S25. Neutropenia funnel plot

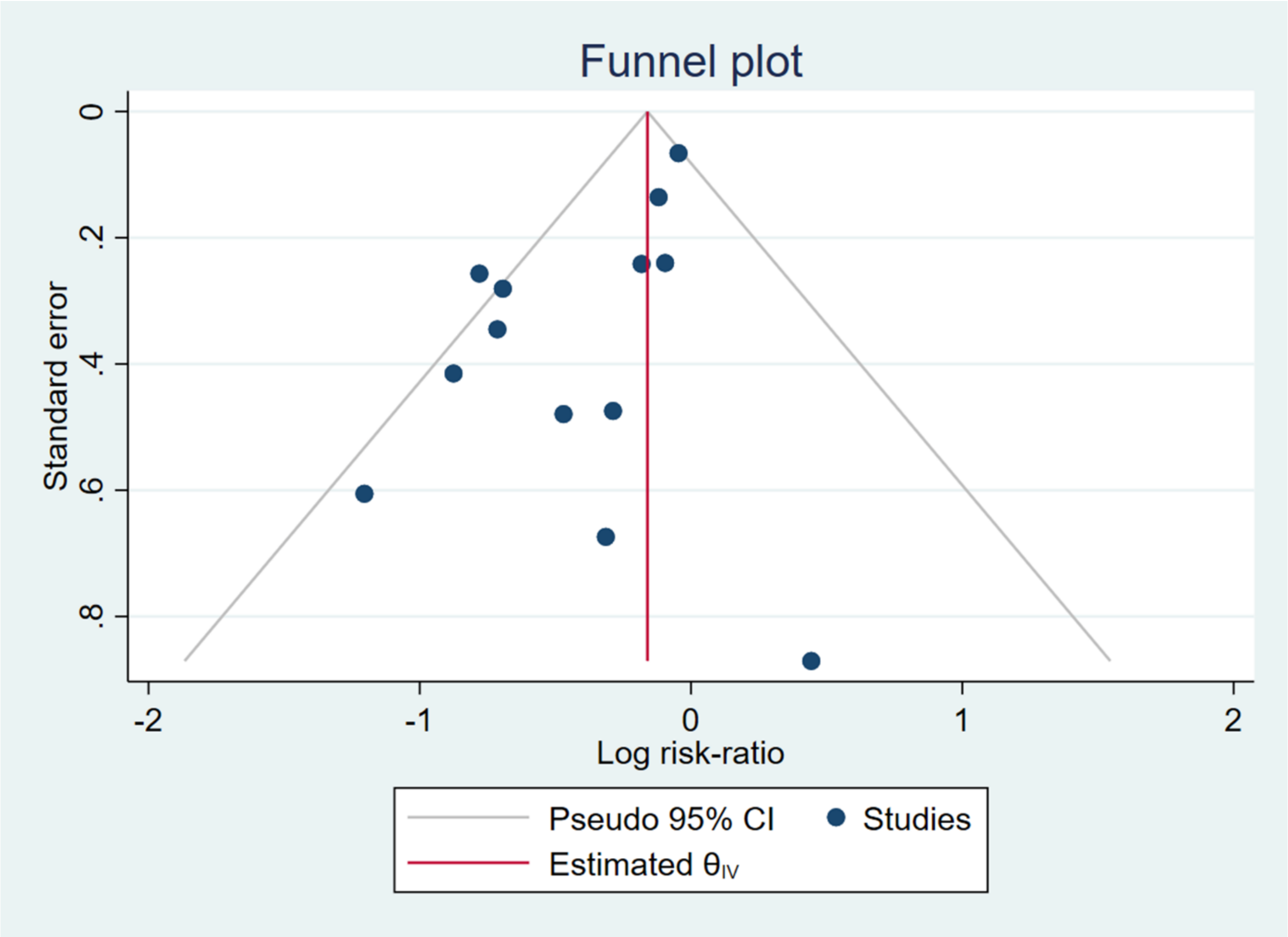

Figure S26.Anemia funnel plot

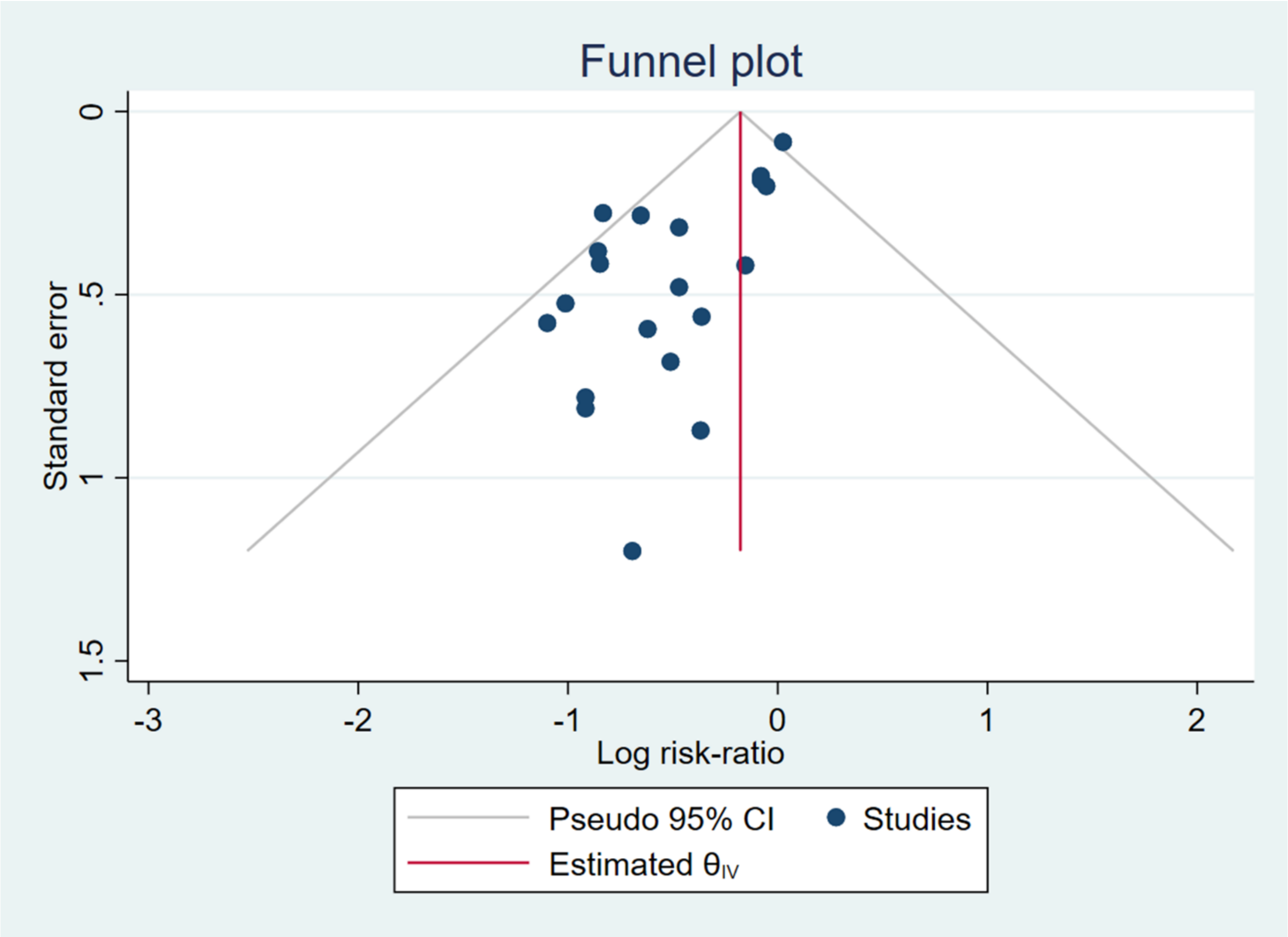

Figure S27.Thrombocytopenia funnel plot

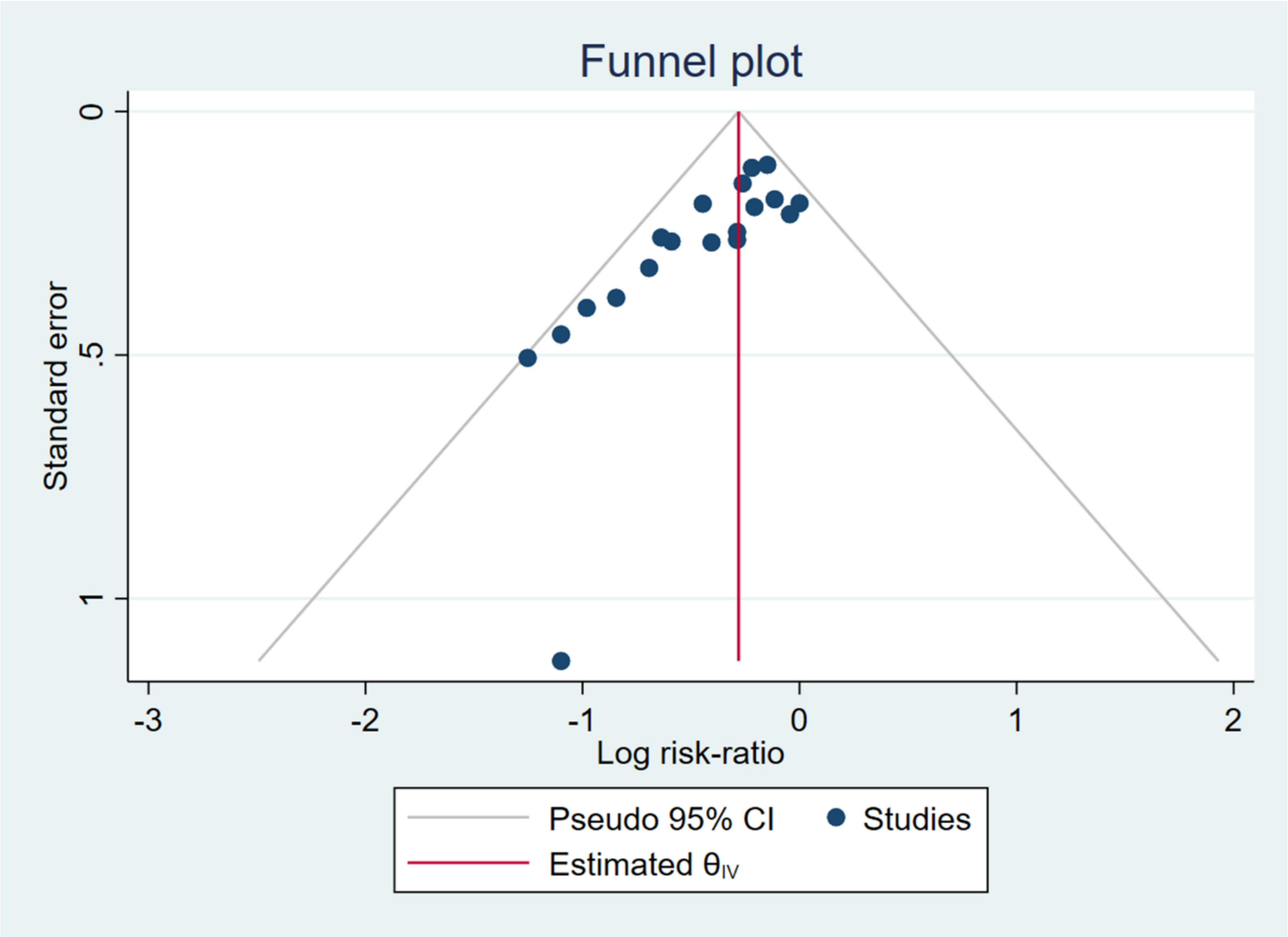

Figure S28.Nausea and vomiting funnel plot

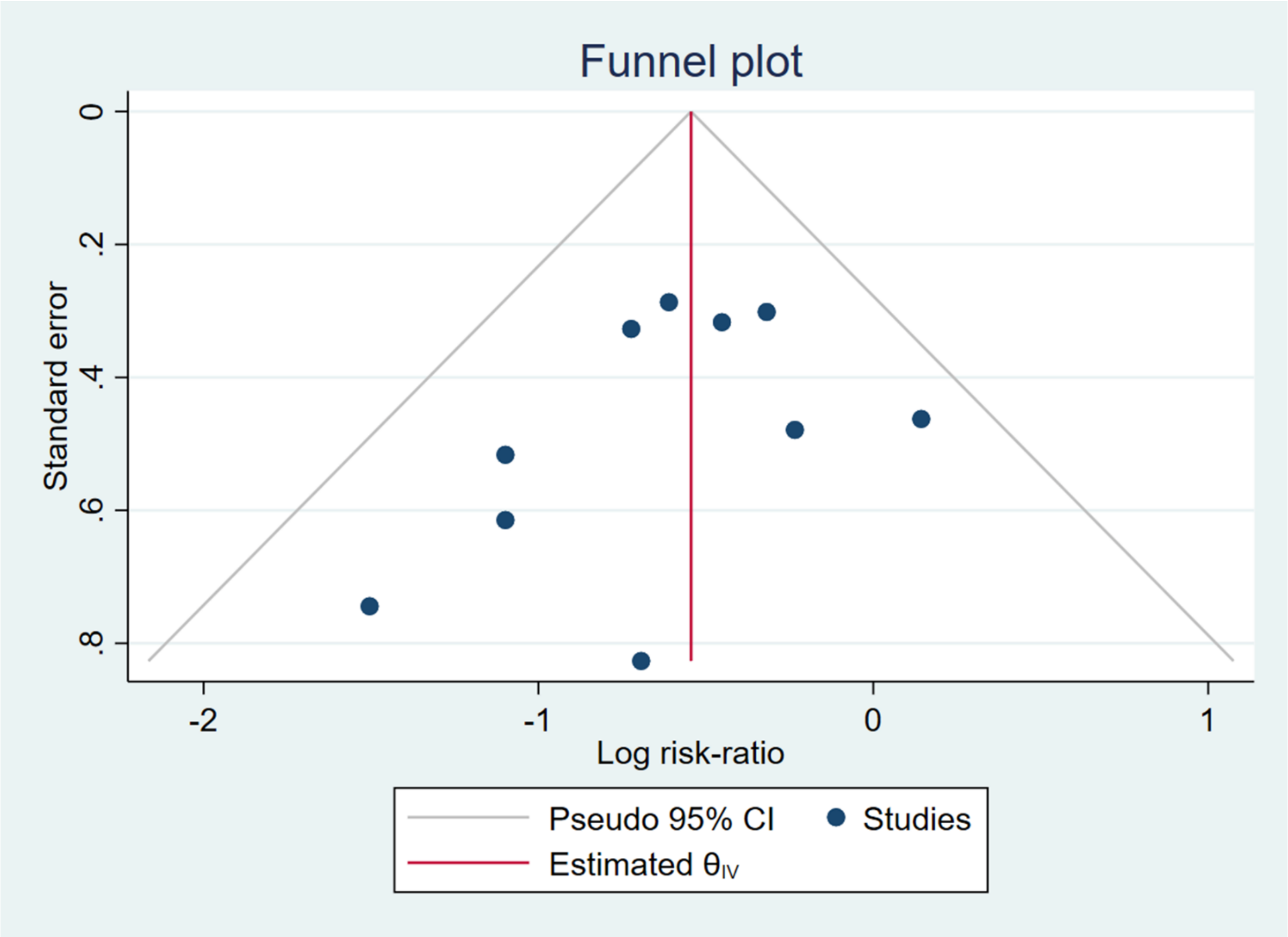

Figure S29. Diarrhea funnel plot

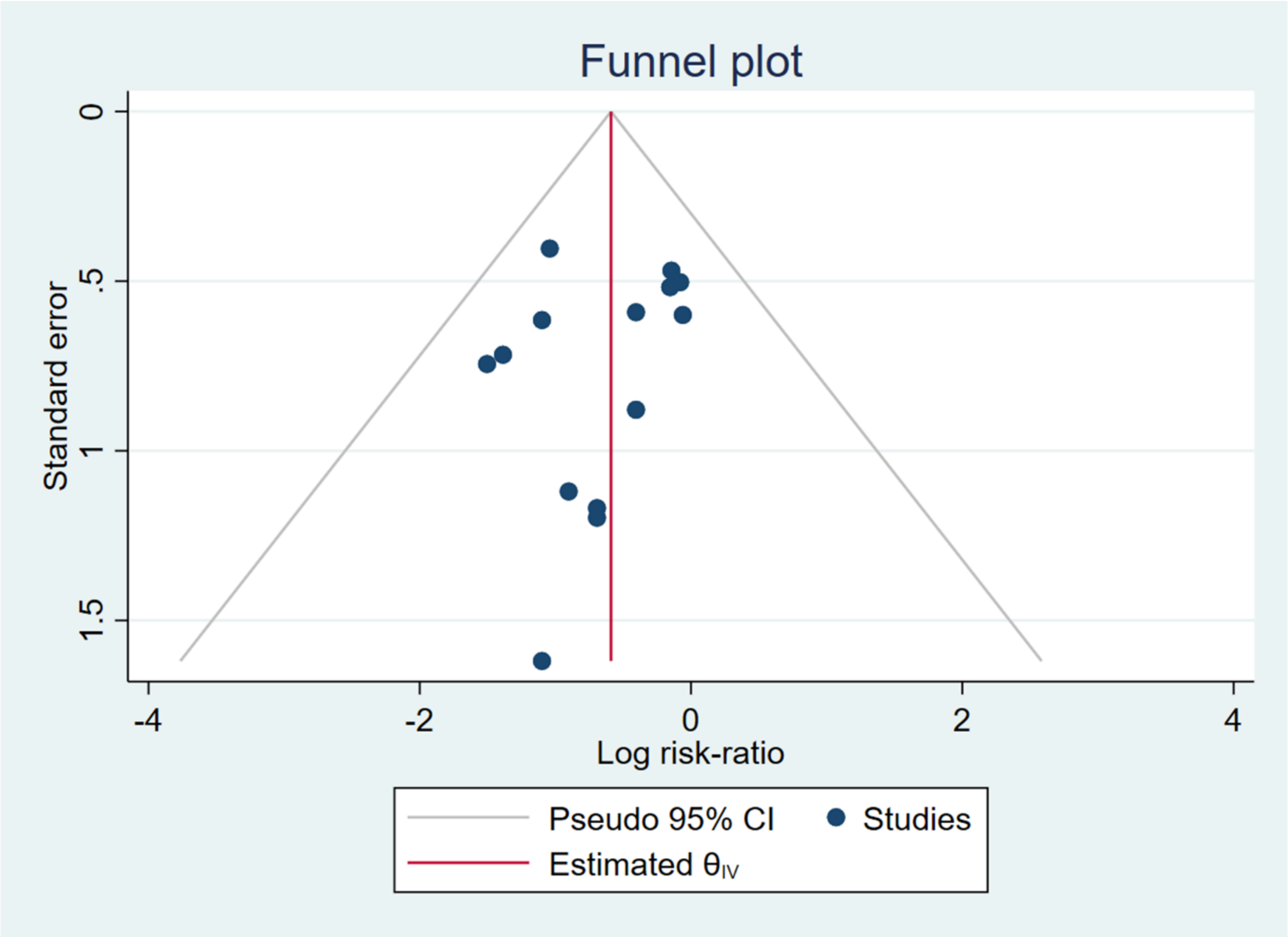

Figure S30.Hepatic Dysfunction funnel plot

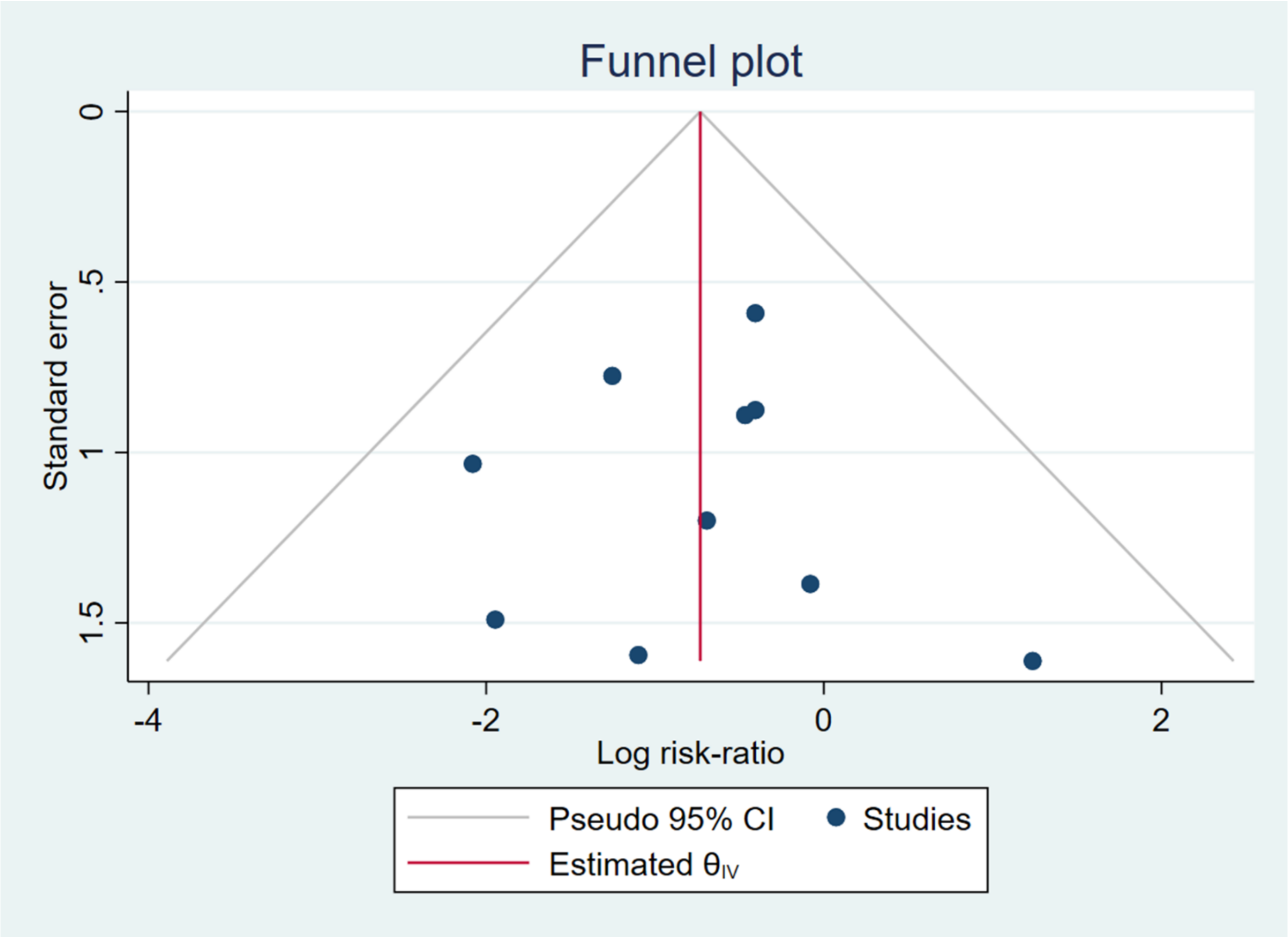

Figure S31.Renal Dysfunction funnel plot

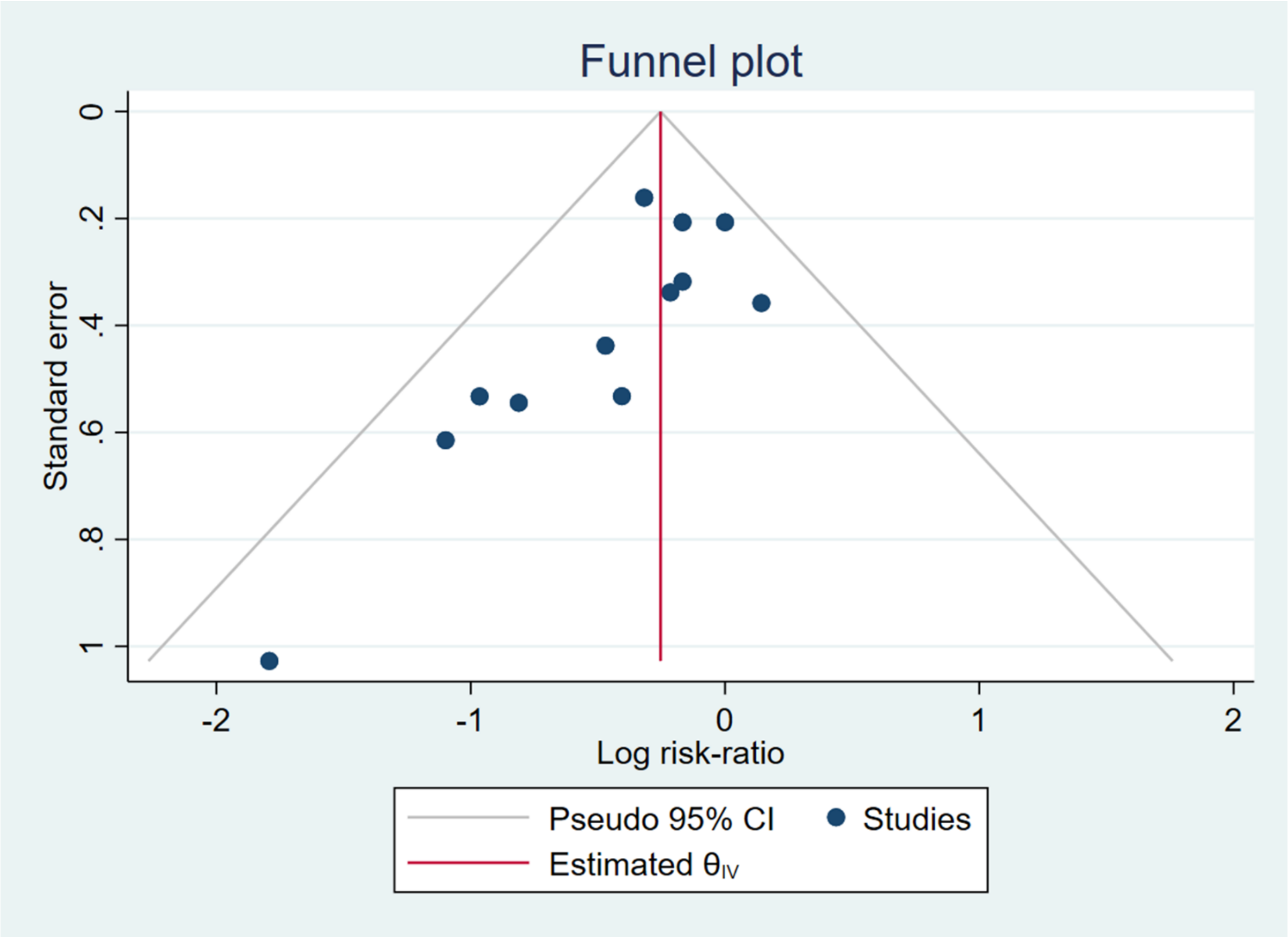

Figure S32.Neurotoxicity funnel plot

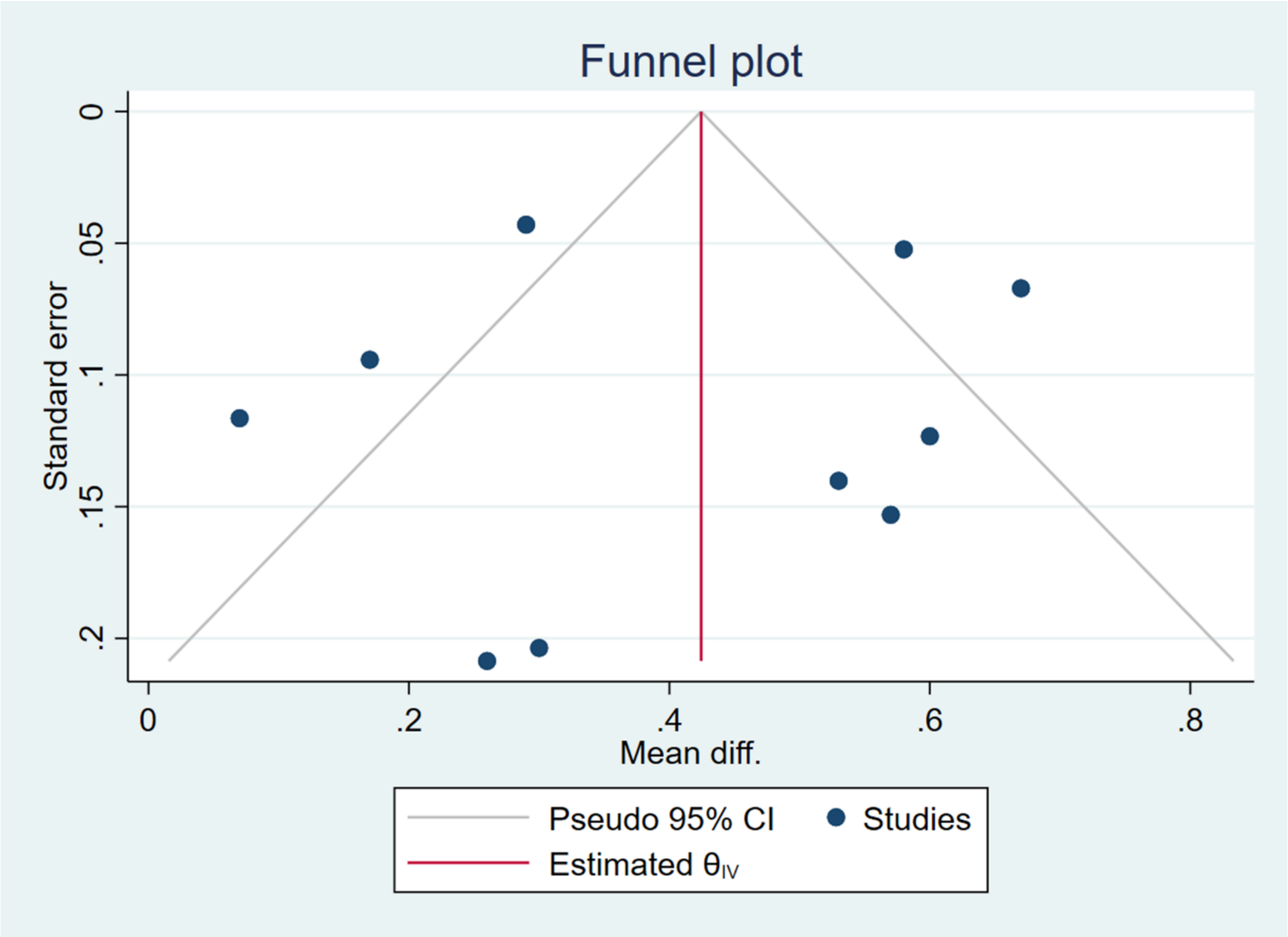

Figure S33.CD4⁺/CD8⁺ ratio funnel plot

2.2 Supplementary Tables

Table S1. Subgroup analysis of outcomes (I^2 > 50%)

Table S1a. Subgroup analysis of Dichotomous outcomes (I^2 > 50%)

Subgroups	Number of studies	RR (95% CI)	Z	P	Heterogeneity	
					I^2	P_h
**1. Half year survival rate**						
**Drug delivery**						
Orally	4	1.12 (0.84, 1.50)	0.79	0.43	91%	<0.00001
Injection	1	1.19 (0.95, 1.50)	1.53	0.13	/	/
**PBC regimens**						
DDP-based chemotherapy regimen	1	1.00 (0.97, 1.04)	0.00	1.00	/	/
OXA-based chemotherapy regimen	4	1.18 (1.05, 1.33)	2.79	0.005	0%	0.68
**Treatment duration**						
≥8 weeks	3	1.07 (0.88, 1.29)	0.67	0.50	74%	0.02
<8 weeks	2	1.24 (1.05, 1.47)	2.53	0.01	0%	0.60
**Data sources**						
from study	3	1.18 (1.03, 1.35)	2.33	0.02	0%	0.47
extracted from survival curve	2	1.08 (0.80, 1.45)	0.51	0.61	85%	0.009
**2. 1-year survival rate**						
**Drug delivery**						
Orally	5	1.62 (0.99, 2.66)	1.92	0.06	85%	<0.0001
Injection	3	1.21 (0.91, 1.60)	1.33	0.18	0%	0.65
**PBC regimens**						
DDP-based chemotherapy regimen	2	1.12 (1.02, 1.23)	2.27	0.02	0%	0.55
OXA-based chemotherapy regimen	6	1.58 (1.27, 1.96)	4.16	<0.0001	0%	0.74
**Treatment duration**						
≥8 weeks	5	1.19 (1.02, 1.38)	3.60	0.0003	0%	0.42
<8 weeks	3	1.83 (1.31, 2.55)	3.54	0.0004	0%	0.85

**Data sources**						
from study	6	1.51 (1.21, 1.90)	2.33	0.02	0%	0.47
extracted from survival curve	2	1.41 (1.09, 1.82)	2.14	0.03	6%	0.30
**3. Neutropenia**						
**Drug delivery**						
Orally	8	0.57 (0.41, 0.78)	3.51	0.0005	67%	0.003
Injection	12	0.74 (0.61, 0.89)	3.12	0.002	71%	<0.0001
**PBC regimens**						
DDP-based chemotherapy regimen	8	0.74 (0.57, 0.96)	2.31	0.02	79%	<0.0001
OXA-based chemotherapy regimen	12	0.61 (0.49, 0.77)	4.30	<0.0001	59%	0.005
**Treatment duration**						
≥8 weeks	17	0.67 (0.55, 0.82)	3.87	0.0001	79%	<0.00001
<8 weeks	3	0.51 (0.23, 1.11)	1.71	0.09	81%	0.005
**4. Anemia**						
**Drug delivery**						
Orally	6	0.72 (0.56, 0.94)	2.45	0.01	40%	0.14
Injection	7	0.63 (0.33, 1.23)	1.35	0.18	83%	<0.00001
**PBC regimens**						
DDP-based chemotherapy regimen	3	0.95 (0.84, 1.08)	0.74	0.46	0%	0.74
OXA-based chemotherapy regimen	10	0.64 (0.50, 0.82)	3.54	0.0004	42%	0.08
**Treatment duration**						
≥8 weeks	10	0.75 (0.57, 0.98)	2.12	0.03	63%	0.004
<8 weeks	3	0.51 (0.35, 0.73)	3.61	0.0003	0%	0.65
**5. Thrombocytopenia**						
**Drug delivery**						
Orally	8	0.60 (0.43, 0.82)	3.18	0.001	32%	0.17
Injection	12	0.70 (0.52, 0.95)	2.28	0.02	62%	0.002
**PBC regimens**						
DDP-based chemotherapy regimen	7	0.98 (0.86, 1.13)	0.26	0.80	0%	0.57
OXA-based chemotherapy regimen	13	0.59 (0.48, 0.73)	4.98	<0.00001	6%	0.39

**Treatment duration**						
≥8 weeks	14	0.76 (0.60, 0.97)	2.22	0.03	52%	0.01
<8 weeks	6	0.48 (0.35, 0.66)	4.61	<0.00001	0%	0.95

Table S1b. Subgroup analysis of Continuous outcomes (I^2＞50%)

Subgroups	Number of studies	Mean difference (95% CI)	Z	P	Heterogeneity	
					I^2	P_h
**1. QOL: according to mean ± SD**						
**Drug delivery**						
Orally	4	14.15 (5.94, 22.37)	3.38	0.0007	96%	<0.00001
Injection	2	6.48 (2.52, 10.44)	3.21	0.001	0%	0.42
**Treatment duration**						
≥8 weeks	4	13.17 (3.67, 22.68)	2.72	0.007	95%	<0.00001
<8 weeks	2	9.84 (7.47, 12.21)	8.13	<0.00001	0%	0.79
**2. $CD3^+$ T cells**						
**Drug delivery**						
Orally	5	14.81 (9.00, 20.61)	5.00	<0.00001	95%	<0.00001
Injection	3	5.73 (1.12, 10.34)	2.44	0.01	89%	<0.0001
**PBC regimens**						
DDP-based chemotherapy regimen	3	11.49 (-2.71, 25.69)	1.59	0.11	99%	<0.00001
OXA-based chemotherapy regimen	5	11.49 (6.76, 16.22)	4.76	<0.00001	93%	<0.00001
**Treatment duration**						
≥8 weeks	5	6.83 (3.05, 10.61)	3.54	0.0004	92%	<0.00001
<8 weeks	3	19.31 (16.03, 22.59)	11.53	<0.00001	72%	0.03
**3. $CD3^+$ $CD4^+$ T cells**						
**Drug delivery**						
Orally	5	6.08 (1.32, 10.85)	2.50	0.01	94%	<0.00001
Injection	3	6.10 (5.24, 6.97)	13.80	<0.00001	19%	0.29
**PBC regimens**						
DDP-based chemotherapy regimen	3	5.14 (1.13, 9.15)	2.51	0.01	90%	<0.0001
OXA-based chemotherapy regimen	5	7.25 (4.16, 10.34)	4.60	<0.00001	91%	<0.00001

**Treatment duration**						
≥8 weeks	5	6.98 (5.12, 8.84)	7.35	<0.00001	85%	<0.0001
<8 weeks	3	5.12 (-3.96, 14.20)	1.11	0.27	95%	<0.00001
**4. Nature Killer cells**						
**Drug delivery**						
Orally	4	3.53 (2.08, 4.99)	4.76	<0.00001	0%	0.89
Injection	2	6.46 (3.01, 9.90)	3.67	0.0002	67%	0.08
**PBC regimens**						
DDP-based chemotherapy regimen	1	4.14 (0.23, 8.05)	2.07	0.04	/	/
OXA-based chemotherapy regimen	5	4.63 (2.17, 7.09)	3.69	0.0002	80%	0.0006
**Treatment duration**						
≥8 weeks	5	4.56 (2.00, 7.12)	3.50	0.0005	80%	0.0006
<8 weeks	1	4.49 (1.39, 7.59)	2.84	0.004	/	/
**5. CD4^{+}/CD8^{+} T cells ratio**						
**Drug delivery**						
Orally	7	0.46 (0.29, 0.63)	5.27	<0.00001	83%	<0.00001
Injection	3	0.29 (0.21, 0.37)	7.12	<0.00001	0%	0.99
**PBC regimens**						
DDP-based chemotherapy regimen	3	0.46 (0.15, 0.76)	2.92	0.003	68%	0.04
OXA-based chemotherapy regimen	7	0.39 (0.24, 0.55)	4.89	<0.00001	84%	<0.00001
**Treatment duration**						
≥8 weeks	7	0.39 (0.24, 0.54)	5.06	<0.00001	78%	0.0001
<8 weeks	3	0.45 (0.09, 0.82)	2.45	0.01	90%	<0.0001

Note: ORR: objective response rate, DCR: disease control rate, QOL: quality of life, CI: confidence interval, OXA: oxaliplatin, DDP: cisplatin.
